# Insights into Long-Lasting Protection Induced by RTS,S/AS02A Malaria Vaccine: Further Results from a Phase IIb Trial in Mozambican Children

**DOI:** 10.1371/journal.pone.0005165

**Published:** 2009-04-14

**Authors:** Caterina Guinovart, John J. Aponte, Jahit Sacarlal, Pedro Aide, Amanda Leach, Quique Bassat, Eusébio Macete, Carlota Dobaño, Marc Lievens, Christian Loucq, W. Ripley Ballou, Joe Cohen, Pedro L. Alonso

**Affiliations:** 1 Barcelona Centre for International Health Research, Hospital Clínic/IDIBAPS, Universitat de Barcelona, Barcelona, Spain; 2 Centro de Investigação em Saúde da Manhiça, Manhiça, Maputo, Mozambique; 3 Faculdade de Medicina, Universidade Eduardo Mondlane, Maputo, Mozambique; 4 Instituto Nacional de Saúde, Maputo, Mozambique; 5 GlaxoSmithKline Biologicals, Rixensart, Belgium; 6 Direcçao Nacional de Saúde, Ministério de Saúde, Maputo, Mozambique; 7 PATH Malaria Vaccine Initiative, Bethesda, Maryland, United States of America; Walter and Eliza Hall Institute of Medical Research, Australia

## Abstract

**Background:**

The pre-erythrocytic malaria vaccine RTS,S/AS02A has shown to confer protection against clinical malaria for at least 21 months in a trial in Mozambican children. Efficacy varied between different endpoints, such as parasitaemia or clinical malaria; however the underlying mechanisms that determine efficacy and its duration remain unknown. We performed a new, exploratory analysis to explore differences in the duration of protection among participants to better understand the protection afforded by RTS,S.

**Methodology/Principal Findings:**

The study was a Phase IIb double-blind, randomized controlled trial in 2022 children aged 1 to 4 years. The trial was designed with two cohorts to estimate vaccine efficacy against two different endpoints: clinical malaria (cohort 1) and infection (cohort 2). Participants were randomly allocated to receive three doses of RTS,S/AS02A or control vaccines. We did a retrospective, unplanned sub-analysis of cohort 2 data using information collected for safety through the health facility-based passive case detection system. Vaccine efficacy against clinical malaria was estimated over the first six-month surveillance period (double-blind phase) and over the following 12 months (single-blind phase), and analysis was per-protocol. Adjusted vaccine efficacy against first clinical malaria episodes in cohort 2 was of 35.4% (95% CI 4.5–56.3; p = 0.029) over the double-blind phase and of 9.0% (−30.6–36.6; p = 0.609) during the single-blind phase.

**Conclusions/Significance:**

Contrary to observations in cohort 1, where efficacy against clinical malaria did not wane over time, in cohort 2 the efficacy decreases with time. We hypothesize that this reduced duration of protection is a result of the early diagnosis and treatment of infections in cohort 2 participants, preventing sufficient exposure to asexual-stage antigens. On the other hand, the long-term protection against clinical disease observed in cohort 1 may be a consequence of a prolonged exposure to low-dose blood-stage asexual parasitaemia.

**Trial Registration:**

ClinicalTrials.gov NCT00197041

## Introduction

Developing a new vaccine is a long and complex process. For example, RTS,S/AS (GSK, Rixensart, Belgium), a pre-erythrocytic vaccine based on *Plasmodium falciparum* circumsporozoite surface protein (CSP) and the candidate malaria vaccine in the most advanced development phase, has been in development for more than two decades. After having demonstrated partial protection against infection in non-immune and semi-immune adults [1]–[3], it underwent proof-of-concept trials in children and infants in Mozambique [4], [5], that were then followed by trials in Kenya and Tanzania [6], [7] prior to the planned launch of wider Phase III efficacy trials.

One of the most critical decisions when preparing a vaccine's clinical development plan is the proper selection of criteria by which the product will be advanced, re-engineered or terminated. Selection of appropriate study endpoints in the various trials that lead up to definitive Phase III efficacy studies is an important part of this process. Different endpoints can be used to estimate efficacy of a pre-erythrocytic vaccine: *P. falciparum* asexual-stage infection, clinical malaria, severe malaria or death. Selection of the endpoint depends on several factors, including the type of vaccine, the phase of the trial and the evidence needed for advocacy and policy decision, and it will determine the sample size and have implications in terms of cost and time. Infection is the endpoint closest to the biological target of the vaccine and is influenced by fewer local cofactors, such as management of malaria cases and parasite and human genetics. As we go downstream (endpoints such as severe malaria or total mortality), the clinical and public health relevance increases, providing stronger evidence for advocacy and policy decision, but the number of cofactors influencing the risk of malaria is larger, potentially decreasing the generalizability of results [8], [9].

In 2003, a randomized controlled Phase IIb proof-of-concept trial was conducted in Mozambique to provide a preliminary estimate of the efficacy, immunogenicity and safety of RTS,S/AS02A malaria vaccine in an age group (1 to 4 years) that would be close to the ultimate target population (infants) [4], [10]. The trial was designed with two cohorts so that it would be possible to estimate vaccine efficacy against two different endpoints: infection and clinical malaria. Cohort 1 was designed to examine efficacy against clinical malaria, because estimation of vaccine efficacy for an endpoint with public health relevance was sought, assessed through health facility-based passive case detection (PCD). During the first six months of follow up (double-blind phase) the vaccine efficacy for the time to first or only clinical malaria episode was 29.9% (95% CI 11.0–44.8; p = 0.004). As an exploratory analysis efficacy against severe malaria was also assessed in this cohort, with an estimate of 57.7% (16.2–80.6; p = 0.019). Anti-CSP antibodies measured one month after the third vaccine dose were not correlated with the risk of clinical malaria. Cohort 2 enrolled a separate group of children who lived in an area with higher transmission intensity and who contributed to the assessment of the efficacy for time to first asexual-stage *P. falciparum* parasitaemia infection. By enrolling this second cohort it was possible to estimate efficacy for a more upstream endpoint and to evaluate how it correlated with efficacy against clinical malaria in Cohort 1. Participants in Cohort 2 were followed up through both active detection of infection (ADI) and PCD. During the double-blind phase the vaccine efficacy for time to first infection was 45.0% (31.4–55.9; p<0.0001) [4].

After unblinding data of the first six months of follow up, participants were followed up for an additional 12 months (single-blind phase), during which vaccine efficacy for the first or only clinical malaria episode in cohort 1 was 28.9% (8.4–44.8; p = 0.008). Therefore the vaccine efficacy did not wane, showing sustained protection during at least 18 months [10]. In cohort 2 almost all children had already had a *P. falciparum* infection during the double-blind phase, therefore it was not possible to continue the ADI during the single-blind phase, in which children were only followed up for safety surveillance through health facility-based PCD.

The correlation between efficacy against clinical malaria in cohort 1 and efficacy against infection in cohort 2 showed that infection could be used as the primary endpoint for efficacy trials of pre-erythrocytic vaccines, which allows conducting smaller trials with high power, decreasing time and cost. Based on these results, a Phase I/IIb randomized controlled trial was recently conducted in infants in the same area to assess the safety, immunogenicity and efficacy of RTS,S/AS02D malaria vaccine, administered at 10, 14 and 18 weeks of age, staggered with the Expanded Program on Immunization vaccines [5]. This infant trial was designed with a single cohort, which was followed up through ADI and PCD, using the same design as for cohort 2 of the previous trial. First or only infection was the main endpoint for evaluation of vaccine efficacy, but further analyses of vaccine efficacy against clinical malaria were explored. During the first three months of follow up, the efficacy against first infection was 65.9% (42.6–79.8; p<0.0001) and that for first or only clinical episode of malaria was 65.8% (25.3–84.4; p = 0.007). In this study in young infants anti-CSP antibodies one month after the third vaccine dose were strongly associated with a reduction in the risk of infection [5].

In these two trials vaccine efficacy estimates vary for different endpoints, transmission intensities and age groups. To provide more evidence on the factors that may influence vaccine response and its duration, we performed a sub-analysis of cohort 2 data from the study in children, that was not included in the original protocol, to estimate vaccine efficacy for clinical malaria in this cohort, using information collected for safety through the health facility-based PCD system.

## Results

417 children were recruited and randomized in cohort 2 (209 received dose one of the RTS,S/AS02A vaccine and 208 received dose one of the control vaccines). Details of the trial profile, the baseline characteristics, the safety, reactogenicity, part of the immunogenicity data and the efficacy against infection in cohort 2 have been previously reported [4], [10].

### Efficacy during the double-blind phase (study months 2.5–8.5) of the ATP cohort

**Figure 1:**
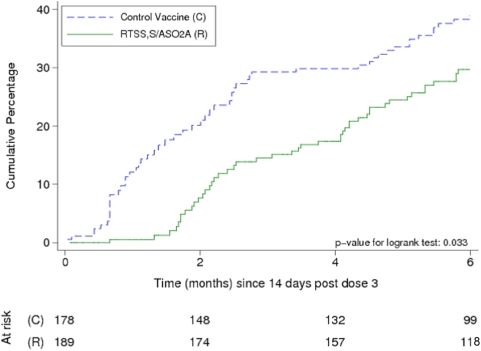
Kaplan-Meier survival curves for the cumulative proportion with at least one episode of clinical malaria during the double-blind and single-blind phases in cohort 2 respectively (ATP cohort).

In the analysis of the according-to-protocol (ATP) cohort over the double-blind phase (study months 2.5–8.5), 102 children had a first episode of clinical malaria (primary case definition) (46 per 72.8 person-years at risk (PYAR) in the RTS,S/AS02A group and 56 per 59.3 PYAR in the control group), giving a crude vaccine efficacy for the time to first or only clinical malaria episode of 34.3% (95% CI 3.0–55.6; p = 0.035) and an adjusted efficacy of 35.4% (95% CI 4.5–56.3; p = 0.029, Table 1). Figure 1 presents the Kaplan-Meier survival curves for the cumulative proportion with at least one episode of clinical malaria in cohort 2. A test for proportionality of hazards showed that these were not constant over time (Schoenfeld residuals p = 0.004), therefore suggesting waning efficacy over the double-blind phase.

**Table 1:** Vaccine efficacy against clinical malaria in cohort 2 (ATP cohort).

Endpoint and follow up period	Control vaccine			RTS,S/AS02A			Vaccine efficacy* (95% CI)	p#
	Events	PYAR§	Rate	Events	PYAR§	Rate		
**Double-blind phase (study months 2.5–8.5)**								
First or only episode of fever and parasitaemia >2500/µL	56	59.3	0.94	46	72.8	0.63	35.4% (4.5; 56.3)	0.029
First or only episode of fever and parasitaemia >15 000/µL	47	61.1	0.77	41	73.8	0.56	30.5% (−5.7; 54.4)	0.089
First or only episode of fever and parasitaemia >50 000/µL	33	64.4	0.51	23	76.4	0.30	42.7% (2.2; 66.4)	0.041
Multiple episodes of fever and parasitaemia >2500/µL	68	70.32	0.97	52	79.2	0.66	30.0% (−1.8; 51.9)	0.062
**Single-blind phase (study months 8.5–21)**								
First or only episode of fever and parasitaemia >2500/µL	59	115.4	0.51	60	123.1	0.49	9.0% (−30.6; 36.6)	0.609

§ Person-years at risk.

# p-value from Cox regression model using Wald test.

* Treatment effect adjusted by: Age at dose 1, Bednet use at baseline, Distance to health centre (Km).

A more detailed analysis of clinical cases occurring in the double-blind phase revealed that 15 children had more than one malaria episode during the double-blind phase (6 in the RTS,S/AS02A group and 9 in the control group) and the adjusted vaccine efficacy for multiple malaria episodes was 30.0% (−1.8–51.9; p = 0.062).

The primary case definition (fever and parasitaemia >2500/µL), that was chosen for cohort 1 based on baseline data from Manhiça, was estimated to be 97.4% sensitive (95% CI 88.8–100.0) and 72.3% specific (49.3–83.6) for cohort 2. Vaccine efficacies using other more specific case definitions are presented in Table 1. The case definitions using fever and parasitaemia >15000 parasites/µL or >50000 parasites/µL had a sensitivity of 82.3% (60.0–98.0) and 60.0% (33.0–85.5) and a specificity of 83.7% (72.1–92.3) and 92.3% (83.7–97.1) respectively.

### Efficacy during the single-blind phase (study months 8.5–21) of the ATP cohort

**Figure 2:**
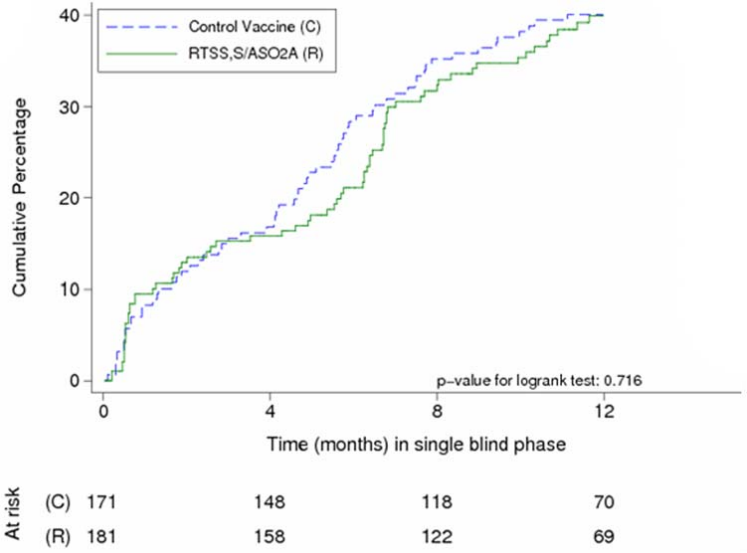
Kaplan-Meier survival curves for the cumulative proportion with at least one episode of clinical malaria during the double-blind and single-blind phases in cohort 2 respectively (ATP cohort).

During the single-blind phase (study months 8.5–21) 119 children of the ATP cohort had a first or only episode of clinical malaria, yielding a crude vaccine efficacy of 6.4% (95% CI −34.0–34.7; p = 0.717) and an adjusted efficacy of 9.0% (−30.6–36.6; p = 0.609). Figure 2 presents the Kaplan-Meier survival curves for the cumulative proportion with at least one episode of clinical malaria in cohort 2.

### Efficacy during the first 3.5 months of the double-blind phase (study months 2.5–6) of the ATP cohort

To be able to compare the vaccine efficacy during the same follow up time period in cohort 2 to that in the infant trial conducted recently in the same area, an analysis was also done for a shorter follow up period of 3.5 months (study months 2.5–6). During this period 111 children in the RTS,S/AS02A group and 147 children in the control group had first episodes of asexual *P. falciparum* parasitaemia, giving an adjusted vaccine efficacy against first infection of 51.1% (37.3–61.9; p<0.0001). Twenty-one children in the RTS,S/AS02A group and 40 children in the control group had a first episode of clinical malaria (primary case definition), yielding an adjusted efficacy for clinical malaria of 61.0% (33.7–77.0; p = 0.0005).

During the double-blind phase the geometric mean density of asexual-stage parasites at the first or only infection was significantly lower in asymptomatic children (1810 parasites/µL) than in children who presented fever (28314 parasites/µL; p<0.0001).

### Efficacy of the ITT cohort

In the analysis of the intention-to-treat (ITT) cohort over the double-blind phase, 148 children had a first episode of clinical malaria (72 in the RTS,S/AS02A group and 76 in the control group), with a crude estimate of vaccine efficacy against clinical malaria of 9.3% (−25.3–34.3; p = 0.555). The corresponding estimate during the single-blind phase was of −4.2% (132 children had a first episode of clinical malaria, −46.6–25.9%; p = 0.813).

### Relation between malaria protection and anti-CSP antibodies

The relation between anti-CSP antibody titers and the risk of infection and clinical malaria during the double-blind phase was evaluated. The hazard ratio for first or only infection per ten-fold increase in the value of anti-CSP IgG was 0.41 (95% CI 0.28–0.60; p<0.0001). When comparing children in the higher tertile against those in the lower tertile value of anti-CSP IgG, the hazard ratio was 0.54 (0.37–0.79; p = 0.002). The equivalent hazard ratios for first or only episode of clinical malaria were 0.99 (0.50–1.94; p = 0.966) and 0.90 (0.44–1.86; p = 0.780) respectively. Thus anti-CSP antibody levels correlated with protection against infection but not with protection against clinical malaria episodes.

## Discussion

In cohort 2, adjusted efficacy of the RTS,S/AS02A candidate malaria vaccine against first or only clinical malaria episodes in Mozambican children aged 1 to 4 years was of 35.4% during the first six months of follow up (ATP cohort), decreasing to 9.0% in the subsequent 12 months of follow up. The follow up of cohort 2 participants, which included both ADI and PCD, was designed to estimate vaccine efficacy against new *P. falciparum* infections. Consequently the sample size for this cohort, based on this endpoint, was much smaller than for cohort 1. Nevertheless, a high incidence of clinical malaria episodes also allowed estimating vaccine efficacy against this endpoint, although with a lower precision.

The primary case definition used (fever and a parasitaemia >2500 parasites/µL) was chosen for cohort 1 based on previous background data from the Manhiça area, where it had been estimated to be 91% specific and 95% sensitive [11]. Using the actual data from cohort 1 this case definition was 95% specific and 86% sensitive (data not shown). This definition had a lower specificity in cohort 2 (72.3%), as malaria transmission in Ilha Josina is higher and children were more immune. Using definitions with higher specificities (fever and parasitaemia >50.000/µL) yielded higher vaccine efficacy estimates (42.7%) [12], similar to the efficacy against first or only infection (45.0% (31.4–55.9; p<0.0001)), assessed for the double-blind phase [4].

Efficacy estimates for clinical malaria in cohort 2 are much lower in the ITT analysis, starting at dose 1 and including the vaccination period, than in the ATP analysis, starting post dose 3. In the ITT analysis time at risk is larger and there are smaller differences in the number of clinical episodes between the RTS,S/AS02A and control group during these first 2.5 months, possibly due to the administration of anti-malarial treatment before dose 3 and that children have not yet received the three vaccine doses, decreasing the differences between the total incidences of the double-blind phase. During the following months, maybe due to the children who have not completed the three doses vaccination course, there is a smaller difference in the number of malaria cases between the RTS,S/AS02A and control group compared to the ATP cohort, thus decreasing the vaccine efficacy.

Data show that cohort 2 children that received RTS,S/AS02A were partially protected against infection and clinical disease in the first six months of follow up post dose 3 (35.4%), at levels similar to cohort 1 (29.9% (95% CI 11.0–44.8; p = 0.004)) [4]. However, thereafter the efficacy against clinical malaria wanes (the evaluation of the proportionality of the hazard assumption reveals that the efficacy in cohort 2 changes with time) and there was no difference in the risk of malaria between RTS,S/AS02A and control recipients during the single-blind phase. This is in sharp contrast to study participants in cohort 1, where vaccine efficacy remained stable, with no evidence of waning for at least 21 months (35.3% (95% CI 21.6–46.6; p<0.0001)) [10].

What are the main differences between cohort 1 and cohort 2 that might explain the discrepancy in vaccine duration of protection? Firstly, the different study design and follow up of participants. In cohort 1 no antimalarial treatment was given to clear parasitaemia before dose 3 and only health facility-based PCD was conducted to detect malaria cases. Therefore those children who became infected with *P. falciparum* had, on average, longer periods of low density parasitaemia, as they were only treated when parasite density reached the fever threshold and the child was taken to the health facility for diagnosis and treatment. Geometric mean parasitaemias (GMPs) at the time of presentation with a clinical malaria episode in cohort 1 were 43522 for the RTS,S/AS02A and 41867 parasites/µL for the control group [4]. On the other hand, in cohort 2 antimalarials were given two weeks before dose 3 and ADI was conducted for six months, during which all children with parasitaemia were treated irrespective of symptoms, their immune system not being exposed to low-density asymptomatic parasitaemias for very long. In cohort 2 the GMPs were similar (3950 parasites/µL in the control group and 3016 in the RTS,S/AS02A group, p = 0.354) at first infection and treatment [4], but significantly lower in those that were asymptomatic than in those with fever (1810 vs. 28314 parasites/µL).

Secondly, the malaria transmission intensity was higher in Ilha Josina, the area where cohort 2 was recruited, as reflected by the geometric mean of antibodies against the whole parasite measured by indirect fluorescent antibody test (IFAT) and the percentage of splenomegaly at baseline. Therefore participants in Cohort 2 may have had a higher level of naturally acquired immunity against infection and clinical malaria when the trial started.

To understand the effect of vaccination and duration of protection, it is difficult to disentangle the effects of the study design from other potential factors such as the immunity level at the time of vaccination and the malaria exposure. RTS,S has shown to extend time to first infection, as seen in cohort 2, which results in reduced risk of clinical malaria, as seen in cohort 1 and 2 during the first 6 months of follow up. In cohort 1 sustained protection was shown for at least 21 months, and this must be a function of either sustained pre-erythrocytic immunity or induction of asexual-stage immunity. Moreover, waning efficacy in cohort 2 can be interpreted as waning pre-erythrocytic immunity. However, similar anti-CSP antibody levels between cohort 1 and 2 (data not shown) would argue against differential pre-erythrocytic immunity explaining the differences in the duration of protection. Based on this line of reasoning, we hypothesize that, as the vaccine-induced pre-erythrocytic immunity declines following the peak levels achieved after vaccination, it only partially inhibits hepatocyte invasion, liver-stage development and release of merozoites to the blood, decreasing the parasite load in the face of a new infection. A low-dose parasitaemia resulting from the partial pre-erythrocytic immunity might be critical to induce or boost the development of asexual blood-stage immune responses, which may confer long-lasting protection against clinical malaria. This low-dose parasitaemia has to be maintained for enough time to stimulate the asexual-stage immune response. Therefore the short-lived vaccine-induced pre-erythrocytic response facilitates the development of a long-lasting asexual-stage immunity in the presence of new infections that act as natural asexual-stage boosters. A vaccine inducing partial pre-erythrocytic protection, like RTS,S/AS, might allow the development of a better and more sustained asexual-stage protection than a more efficacious vaccine, by allowing this “leakage” of low-dose parasites.

This is consistent with other recent hypotheses [13] and observations in studies that assessed the capacity of low parasitaemias to induce or maintain protective immune responses [14]–[17]. Similar mechanisms have also been proposed to explain the sustained protection of intermittent preventive treatment with sulfadoxine-pyrimethamine administered in infancy (IPTi) in Tanzanian children [18].

Children in cohort 1 were probably exposed to low-density parasitaemias for a longer time than children in cohort 2, in which the development of this enhanced asexual-stage immune response may have been impaired. We propose this may explain a waning of the vaccine-specific protective response in cohort 2.

The short duration of protection in cohort 2 is not surprising and is similar to that observed in other RTS,S trials [1], [3] or irradiated sporozoites trials [19]. Despite differences in the age of participants and endemicity of malaria, results were comparable to those of cohort 2 in Mozambique. Furthermore, during the first three months of follow up after the third vaccine dose the efficacy of RTS,S was between 40 and 70% for all trials (infants [5], cohort 1 (data not shown) and cohort 2 of children aged 1 to 4 years, and adults [1]), irrespective of age or transmission intensity, when using a highly specific malaria case definition. The initial response to the RTS,S/AS02 does not seem to vary in the different trials, what appears to change is the ability to induce a long-lasting protective response.

With regard to antibody responses to the vaccine in cohort 2 children, the level of anti-CSP IgG was correlated with a lower risk of infection but not with a lower risk of clinical malaria. In cohort 1 it had not been correlated with the risk of clinical malaria either [4] and in the infant trial it had also been correlated with a lower risk of infection [5]. In addition, antibody levels decayed over the double-blind phase, but remained at the end of the single-blind phase at levels 40 fold higher than in controls [10]. This indicates that anti-CSP antibodies, probably together with other cellular immune responses, may be involved in the initial protection and are correlated with protection against infection, supporting the above-mentioned hypothesis. Nevertheless, other unknown immune mechanisms, most likely involving priming of asexual-stage humoral and cellular immunity, developed as the pre-erythrocytic immunity decays, may be responsible for the long-lasting protection against disease. This points towards the need to assess antibody and cellular asexual-stage immune responses in future Phase III RTS,S/AS vaccine trials.

When designing future pre-erythrocytic malaria vaccine trials it has to be taken into account that the study design might have a great impact on the duration of protection. ADI with rapid treatment of parasitaemias might impair the development of long-lasting protection, although initial efficacy seems to be independent of study design, age or malaria transmission intensity. If assessment of duration of protection is included in the trial objectives the design should consist only of a PCD follow up. Otherwise, if the main aim is to obtain quick efficacy results, a smaller trial with *P. falciparum* infection as the primary endpoint and ADI can be used.

In conclusion, the preponderance of data discussed leads to the following hypothesis: that the long-term protection against clinical disease observed in RTS,S/AS02A recipients is a consequence of a partially protective vaccine-induced pre-erythrocytic response that lasts several months, and limits the number of viable sporozoites and merozoites emerging from the liver to initiate the blood stage cycle of the infection. This leads to prolonged exposure to low-dose asexual blood-stage parasites that allows the acquisition of long-lasting asexual blood-stage immunity. Study designs that include prompt ascertainment and treatment of infections in the absence of symptoms may modify long-term protection. Vaccination in infancy therefore has the potential to confer important levels of protection through a time of high susceptibility in early childhood. A fuller understanding of the mechanism of vaccine action including determination of the efficacy and duration under varying conditions of malaria transmission will be reached through the conduct of properly designed Phase III trials.

## Materials and Methods

The protocol for this trial and supporting CONSORT checklist are available as supporting information; see Checklist S1 and Protocol S1.

### Study design

The trial was conducted at the Centro de Investigação em Saúde da Manhiça (CISM, Manhiça Health Research Centre), in Manhiça District (Maputo Province), southern Mozambique. The area is under demographic surveillance system (DSS) and has been described in detail elsewhere [20]. Adjacent to CISM is the Manhiça District Hospital (110 beds), the main referral hospital in the area. The climate is subtropical with a rainy season from November to April and a cool and dry season during the rest of the year. Malaria transmission, mainly caused by *P. falciparum*, is perennial with marked seasonality. The trial was conducted in two different areas: Manhiça and Maragra, where cohort 1 (n = 1605) was recruited, and Ilha Josina, 55 km north of Manhiça, where cohort 2 (n = 417) was recruited. The estimated entomological inoculation rate for the Manhiça area in 2002 was 38 infective bites/person/year, being *Anopheles funestus* the main vector. In Ilha Josina the transmission is higher than in Manhiça, as reflected by a significantly higher geometric mean of antibodies against the whole parasite as assessed by IFAT and percentage of splenomegaly at baseline in study participants recruited in that area [4].

The study was a Phase IIb double-blind, randomized controlled trial in children aged 1 to 4 years to assess the efficacy, immunogenicity and safety of RTS,S/AS02A candidate malaria vaccine according to a 0, 1, 2 month vaccination schedule [4].

**Figure 3:**
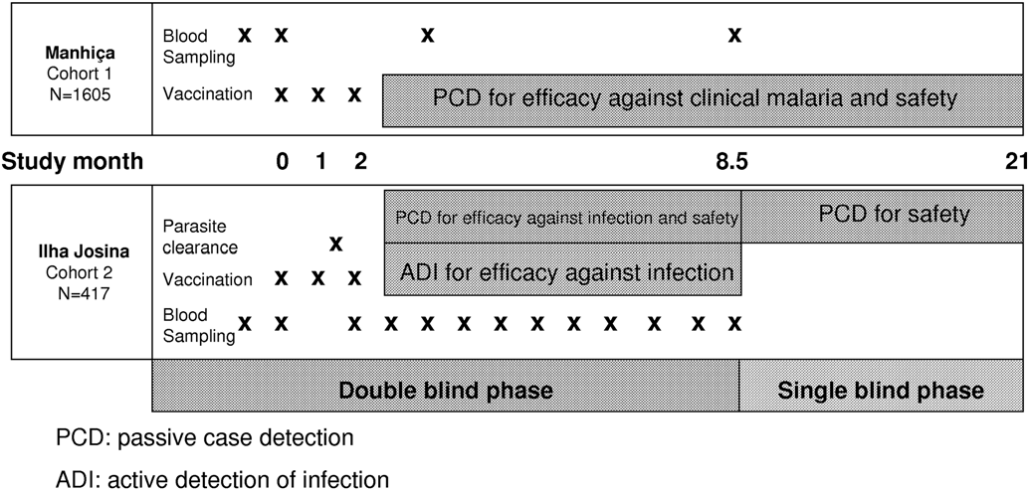
Study design.

The study design has been described in detail elsewhere [4], [10]. This paper presents a retrospective, unplanned sub-analysis of cohort 2 data. According to protocol, the surveillance period started 14 days after dose 3. Participants were followed for six months during the double-blind phase (study months 2.5–8.5), after which data were unblinded and analyzed, and were then followed up for 12 additional months during the single-blind phase (study months 8.5–21). Figure 3 presents the study design and follow up phases.

In the original protocol, the efficacy endpoint in cohort 2, assessed during the double-blind phase, was first *P. falciparum* infection, as detected by ADI or health facility-based PCD. According to protocol, information on clinical malaria episodes of cohort 2 participants was collected through PCD throughout the double and single-blind phases, with the objective of using these data for monitoring safety rather than the formal evaluation of vaccine efficacy. The rationale for that was that interpretation of efficacy results of clinical malaria disease collected at health centers would be compromised by the ADI visits and the sample size would be inadequate. However, despite the limitations of the study design and sample size, to gain further insights into the mechanism of long lasting protection, we have now performed an exploratory analysis, using these data to estimate incidence of clinical malaria and vaccine efficacy against clinical malaria in cohort 2.

The protocol was approved by the National Mozambican Ethics Review Committee, the Hospital Clínic of Barcelona Ethics Review Committee and the PATH Human Subjects Protection Committee. The trial was conducted according to the International Conference on Harmonisation good clinical practice guidelines, and was monitored by GSK Biologicals. A local safety monitor and a data and safety monitoring board closely reviewed the conduct, safety and data of the trial.

The study was registered with the ClinicalTrials.gov identifier NCT00197041.

### Procedures

Screening, informed consent, enrolment, randomization, immunization and safety assessment were done as previously described [4], [10]. Participants were randomized to receive three doses of either RTS,S/AS02A candidate malaria vaccine or a control vaccine. RTS,S is a pre-erythrocytic vaccine based on *P. falciparum* CSP, that is fused to the S antigen of the hepatitis B virus, and is formulated with the AS02A Proprietary Adjuvant System. Details of the formulation and dosing of the vaccine have been reported elsewhere [4]. The control vaccines for children younger than 24 months were two doses of the seven-valent pneumococcal conjugate vaccine (*Prevenar™* Wyeth Lederle Vaccines) and one dose of *Haemophilus influenzae type b* vaccine (GSK Biologicals) and for children aged ≥24 months the pediatric hepatitis B vaccine (GSK Biologicals).

A round-the-clock health facility-based morbidity surveillance system was operating at the Manhiça District Hospital and the Maragra and Ilha Josina health posts throughout the study. A standardized questionnaire form, which includes personal and demographic data and clinical signs and symptoms, was completed for each child seen at the outpatient clinic. The axillary temperature was measured with an electronic thermometer and recorded and a finger-prick blood sample was collected from all children who present fever (axillary temperature ≥37.5°C) or report a history of fever in the preceding 24 hours. Blood was collected into heparinized capillaries to measure the packed cell volume (PCV) and thin and thick blood smears were prepared to determine parasitaemia. The first line malaria treatment for non complicated malaria at the time of the study was sulfadoxine-pyrimethamine and amodiaquine and the second-line treatment was Coartem® (artemether-lumefantrine) (Novartis).

In cohort 2 participants were followed up by a combination of ADI and PCD during the double-blind phase. Antimalarials (a single oral dose of sulfadoxine 25 mg/kg plus pyrimethamine 1.25 mg/kg and amodiaquine 10 mg/kg for 3 days) were administered to all participants 14 days before dose 3 to presumptively clear parasites. Parasitaemia was checked two weeks later and, if positive, the child was treated with the second line antimalarial treatment and was not included in the assessment for ADI. Surveillance for malaria infection was started 14 days after dose 3 and was done throughout the double-blind phase (study months 2.5–8.5) through ADI visits performed every two weeks for 2.5 months and monthly for the following two months. During the ADI a field worker visited participants at home, completed a brief morbidity questionnaire and measured the axillary temperature. If the child was afebrile the field worker collected blood by fingerprick onto slides and filter paper. If the child had fever or a history of fever in the preceding 24 hours, the field worker accompanied the child to the Ilha Josina health post, where he or she was examined and blood slides and filter paper were collected. All children with *P. falciparum* parasitaemia received antimalarial treatment, irrespective of symptoms, and were excluded from subsequent ADI visits.

Antibodies against CSP were measured before dose one and 30 days after dose three and IFAT and spleen size (Hackett's scale) were measured at screening.

### Laboratory methods

Blood slides were Giemsa-stained and read following standard quality-controlled procedures [21]. External validation was done at the Hospital Clínic of Barcelona, Spain. The PCV was measured using a microhematocrit centrifuge and a Hawksley reader (Hawksley & Sons Ltd, Lancing, UK).

IgG antibodies specific for the CSP tandem repeat epitope were measured by a standard ELISA with plates adsorbed with the recombinant antigen R32LR that contains the sequence [NVDP(NANP)15]2LR, using a standard serum as reference [22]. For the IFAT, 25 µL of test sera (two-fold serial dilutions up to 1/81920) were incubated with *P. falciparum*-infected red blood cells fixed onto a 12-well slide. Positive reactions were revealed with fluorescein isothiocyanate-labelled secondary antibody diluted in Evans blue. The highest dilution giving positive fluorescence under an ultraviolet light microscope was scored.

### Statistical methods and case definitions

Trial results presented previously [4], [10] were analyzed following a report and analysis plan established before unblinding.

The results presented here are exploratory analyses of cohort 2 that were not described in the protocol, performed on data collected to 21 months post-study start. The endpoint of this sub-analysis was first or only clinical episode of *P. falciparum* malaria. A clinical episode was defined as a child with an axillary temperature of ≥37.5°C and a *P. falciparum* asexual parasitaemia of >2500 parasites/µL on the blood slide (primary case definition), detected through the health-facility based PCD or the ADI visits. Analyses were performed on the ATP cohort, which was defined as children who met all eligibility criteria, received the complete vaccination course and contributed to the efficacy surveillance. Time at risk started 14 days after dose 3 and the analysis was conducted for the time periods 2.5 to 8.5 (double-blind phase) and 8.5 to 21 (single-blind phase) study months. For data pertaining to the period 2.5 to 8.5 vaccine efficacy for other definitions of clinical malaria using different cut-offs for parasitaemia and assessment of vaccine efficacy for multiple malaria episodes was also calculated.

Absences from the study area of two or more weeks and a time interval after antimalarial drug use (28 days after sulfadoxine-pyrimethamine, 7 days after chloroquine alone, 7 days after quinine alone, 7 days after amodiaquine and 20 days after artemether+lumefantrine) were not included in the time at risk. If the combination of drugs was given, the longest period was used. For the analysis of multiple episodes of clinical malaria, we did not judge a child to be susceptible for 28 days after the previous episode. Vaccine efficacy for the time to first or only clinical malaria episode was assessed using Cox regression models and defined as (1−Hazard Ratio). Vaccine efficacy was adjusted for the covariates: age at dose 1, bednet use at baseline and distance from the health centre (as determined by geopositioning of every household with a handheld global positioning system with differential correction). The interaction between age and vaccine efficacy was not significant for any of the follow up periods, so an interaction term was not included. The proportional hazards assumption was investigated graphically, using a test based on the Schoenfeld residuals [23] and time-dependent Cox models [24] using interactions between the vaccine effect and one-degree fractional polynomials of the time.

For multiple episodes of clinical malaria the vaccine effect was assessed using Poisson regression models with normal random intercepts, including the time at risk as an offset variable. Vaccine efficacy was defined as (1−Rate Ratio). The difference in the geometric mean of the positive densities was assessed with the non-parametric Wilcoxon test.

The sensitivity and specificity of different case definitions were estimated for cohort 2 participants using data from the study month 8.5 visit, following the methodology described by Smith and colleagues [25].

Results of the ITT cohort are also presented. All children who received at least one vaccine dose were included and efficacy estimates were not adjusted for covariates. Time at risk started from dose 1 and was not adjusted for absences from the study area or antimalarial drug use.

The relation between anti-CSP antibody titers as measured 30 days post dose 3 and the risk of infection and clinical malaria was assessed in RTS,S/AS02A recipients. The hazard ratio of participants with anti-CSP antibodies in the higher tertile against those in the lower tertile was estimated, as well as the hazard ratio per ten-fold increase in the value of anti-CSP antibodies, using Cox regression models.

The sample size of cohort 2 was calculated at the beginning of the study to estimate vaccine efficacy against first or only infection in cohort 2, which has been previously reported [4]. Based on the incidence of clinical malaria in the control group of cohort 2 (0.94 episodes per PYAR during the double-blind phase and 0.51 during the single-blind phase), the power to detect a vaccine efficacy against clinical malaria of 40% or higher at a 5% significance level during the first six months of follow up (double-blind phase) is of 65.8% and during the following 12 months (single-blind phase) is of 68.0%.

Analyses were done using STATA version 10.0 (College Station, TX, USA).

## Supporting Information

Checklist S1 CONSORT Checklist
(0.19 MB DOC)

Protocol S1 Trial Protocol
(1.19 MB DOC)
