# Supplementary material for: Insights into Long-Lasting Protection Induced by RTS,S/AS02A Malaria Vaccine: Further Results from a Phase IIb Trial in Mozambican Children
Source: PLoS One. 2009 Apr 14;4(4):e5165. doi: 10.1371/journal.pone.0005165 (PMC2666156; doi:10.1371/journal.pone.0005165)
Supplement: Checklist S1 — CONSORT Checklist (0.19 MB DOC) [file pone.0005165.s001.doc]

# CONSORT Statement 2001 - Checklist
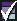


**Items to include when reporting a randomized trial**

| ***PAPER SECTION* And topic** | Item | **Descriptor** | **Reported on**  **Page #** |
| --- | --- | --- | --- |
| TITLE & ABSTRACT | 1 | Participants were randomized, but this is not specified in the title as this is an exploratory analysis performed after the results of the original trial were already published, and there is not enough space in the title to include it. It was included in the title of the two articles reporting the primary analyses (Alonso PL 2004; Alonso 2005). | Abstract, Materials and methods (Study design) |
| *INTRODUCTION* Background | 2 | Developing a new vaccine is a long and complex process. For example, RTS,S/AS02A (GSK, Rixensart, Belgium), a pre-erythrocytic vaccine based on *Plasmodium falciparum* circumsporozoite surface protein (CSP) and the candidate malaria vaccine in the most advanced development phase, has been in development for more than two decades. After having demonstrated partial protection against infection in non-immune and semi-immune adults, it underwent proof-of-concept trials in children and infants in Mozambique prior to the planned launch of wider Phase III efficacy trials.  One of the most critical decisions when preparing a vaccine’s clinical development plan is the proper selection of criteria by which the product will be advanced, re-engineered or terminated. Selection of appropriate study endpoints in the various trials that lead up to definitive Phase III efficacy studies is an important part of this process. Different endpoints can be used to estimate efficacy of a pre-erythrocytic vaccine: *P. falciparum* asexual-stage infection, clinical malaria, severe malaria or death. Selection of the endpoint depends on several factors, including the type of vaccine, the phase of the trial and the evidence needed for advocacy and policy decision, and it will determine the sample size and have implications in terms of cost and time. Infection is the endpoint closest to the biological target of the vaccine and is the least influenced by local cofactors, such as management of malaria cases and parasite and human genetics. As we go downstream, the clinical and public health relevance increases, but the number of cofactors influencing the risk of malaria is larger, potentially decreasing the generalizability of results.  In 2003, a randomized controlled phase IIb proof-of-concept trial was conducted in Mozambique to provide a preliminary estimate of the efficacy, immunogenicity and safety of RTS,S/AS02A malaria vaccine in an age group (1 to 4 years) that would be close to the ultimate target population (infants). The trial was designed with two cohorts so that it would be possible to estimate vaccine efficacy against two different endpoints: infection and clinical malaria. Cohort 1 was designed to examine efficacy against clinical malaria, because estimation of vaccine efficacy for an endpoint with public health relevance was sought, assessed through health facility-based passive case detection (PCD). During the first six months of follow up (double-blind phase) the vaccine efficacy for the first or only clinical malaria episode was 29.9% (95% CI 11.0-44.9; p=0.004). As an exploratory analysis efficacy against severe malaria was also assessed in this cohort, with an estimate of 57.5% (16.2-80.6; p=0.019). Anti-CSP antibodies were not correlated with the risk of clinical malaria. Cohort 2 enrolled a separate group of children who lived in an area with higher transmission intensity and who contributed to the assessment of the efficacy against first asexual stage *P. falciparum* parasitemia infection. By enrolling this second cohort it was possible to estimate efficacy for a more upstream endpoint and to evaluate how it correlated with efficacy against clinical malaria in Cohort 1. Participants in Cohort 2 were followed up through both active detection of infection (ADI) and PCD. During the double-blind phase the vaccine efficacy against first infection was 45.0% (31.4-55.9; p<0.0001).  After unblinding data of the first six months of follow up, participants were followed up for an additional 12 months (single-blind phase), during which vaccine efficacy for the first or only clinical malaria episode in cohort 1 was 28.9% (8.4-44.8; p=0.008). Therefore the vaccine efficacy did not wane, showing sustained protection during at least 18 months. In cohort 2 almost all children had already had a *P. falciparum* infection during the double-blind phase, therefore it was not possible to continue the ADI during the single-blind phase, in which children were only followed up for safety surveillance through health facility-based PCD.  The correlation between efficacy against clinical malaria in cohort 1 and efficacy against infection in cohort 2 showed that infection could be consistently used as the primary endpoint for efficacy trials of pre-erythrocytic vaccines, which allows conducting smaller trials with high power, decreasing time and cost. Based on these results, a phase I/IIb randomized controlled trial was recently conducted in infants in the same area to assess the safety, immunogenicity and efficacy of RTS,S/AS02D malaria vaccine, administered at 10, 14 and 18 weeks of age, staggered with the Expanded Program on Immunization vaccines. This infant trial was designed with a single cohort, which was followed up through ADI and PCD, using the same design as for cohort 2 of the previous trial. First or only infection was the main endpoint for evaluation of vaccine efficacy, but further analyses of vaccine efficacy against clinical malaria were explored. During the first three months of follow up, the efficacy against first infection was 65.9% (42.6-79.8; p<0.0001) and that for first or only clinical episode of malaria was 65.8% (25.3-84.4; p=0.007). In this study in young infants anti-CSP antibodies were strongly associated with a reduction in the risk of infection.  In these two trials vaccine efficacy estimates differ for different endpoints, transmission intensities and age groups. To provide more evidence on the factors that may influence vaccine response and its duration, we performed a sub-analysis of cohort 2 data from the study in children, that was not included in the original protocol, to estimate vaccine efficacy for clinical malaria in this cohort, using information collected for safety through the health facility-based PCD system. | Introduction |
| *METHODS* Participants | 3 | Eligibility criteria are not specified in the current manuscript and were summarized in the article presenting the primary study (Alonso PL 2004).  Inclusion criteria:   - Healthy male and female children, 1 to 4 years of age at the time of first vaccination (up to but not including 5^th^ birthday). - Written/thumbprinted and witnessed informed consent obtained from the parents or legal guardians, after they have been informed of the risks and benefits of the study in a language, which they clearly understand. - Free of obvious health problems as established by medical history and clinical examination before entering into the study. - Availability for the duration of the immunisation and follow-up period   Exclusion criteria:   - Major congenital defects or serious chronic illness. Acute or chronic, clinically significant pulmonary, cardiovascular, hepatic or renal functional abnormality, as determined by physical examination or laboratory screening tests (see below). Chronic carriers of the HBsAg will not be excluded from the clinical trial provided they are clinically asymptomatic. - Haematocrit of less than 25%. - History of allergic reactions to vaccination. - History of allergic reactions to any component of the Hiberix™, Prevnar® or Engerix-B™ vaccines. - Chronic administration (defined as more than 14 days) of immuno-suppressants or other immune modifying drugs within six months prior to the first vaccine dose (for corticosteroids, this will mean prednisone, or equivalent, ≥ 0.5 mg/kg/day. Inhaled and topical steroids are allowed.) - Previous vaccination with an experimental vaccine. - Previous vaccination with hepatitis B vaccine (as documented on health card) in children equal to or more than 24 months of age. - Simultaneous participation in any other clinical trial. - Any other findings which in the opinion of the investigators would increase the risk of having an adverse outcome from participation in the trial. - Severe malnutrition defined as weight for height ≤ -3z scores. - History of surgical splenectomy. - Use of any investigational or non-registered drug or vaccine other than the study vaccine(s) within 30 days preceding the first dose of study vaccine, or planned use during the study period. - Planned administration of a vaccine not foreseen by the study protocol within 30 days before the first dose of vaccine. An exception, is the receipt of an EPI or licensed vaccine (measles, oral polio, meningococcal and combined diphtheria/pertussis/tetanus vaccines) which may be given 14 days or more before or after vaccination. - Administration of immunoglobulins and/or any blood products within the three months preceding the first dose of study vaccine or planned administration during the study period. - A diagnosis or clinical suspicion of an immunosuppressive or immunodeficient condition of any cause based on a full clinical history and medical examination. - A family history of congenital or hereditary immunodeficiency. - Same-sex twin. - Lack of healthcard (i.e. no previous vaccination record).   Study site  The trial was conducted at the Centro de Investigação em Saúde da Manhiça (CISM, Manhiça Health Research Centre), in Manhiça District (Maputo Province), southern Mozambique. The area is under demographic surveillance system (DSS) and has been described in detail elsewhere. Adjacent to CISM is the Manhiça District Hospital (110 beds), the main referral hospital in the area. The climate is subtropical with a rainy season from November to April and a cool and dry season during the rest of the year. Malaria transmission, mainly caused by *P. falciparum*, is perennial with marked seasonality. The trial was conducted in two different areas: Manhiça and Maragra, where cohort 1 (n=1605) was recruited, and Ilha Josina, 55 km north of Manhiça, where cohort 2 (n=417) was recruited. The estimated entomological inoculation rate for the Manhiça area in 2002 was 38 infective bites/person/year, being *Anopheles funestus* the main vector. In Ilha Josina the transmission is higher than in Manhiça, as reflected by a significantly higher geometric mean of antibodies against the whole parasite as assessed by indirect fluorescent antibody test (IFAT) and percentage of splenomegaly at baseline in study participants recruited in that area.  More detail was given in the article reporting the primary analysis (Alonso PL 2004). | NA  Materials and methods (Study design) |
| Interventions | 4 | Vaccination procedures are only briefly described in the current manuscript, but were described in detail in the article reporting the primary analysis:  RTS,S consists of a hybrid molecule recombinantly expressed in yeast, in which the circumsporozoite protein, 9 central tandem repeat, and carboxyl-terminal regions are fused to the N terminal of the S antigen of hepatitis B virus (HBsAg) in a particle that also incluyes the unfused S antigen. A full adult dose of RTS,S/AS02A (GSK Biologicals, Rixensart, Belgium) contains 50 _g of lyophilised RTS,S antigen reconstituted in 500 _L of AS02A adjuvant (proprietary oil in water emulsion with the immunostimulants monophosphoryl lipid A [MPL; Corixa, Seattle, WA, USA] and *Quillaja saponaria* fraction 21 [QS21; Antigenics, New York, NY, USA]). We used half the adult dose in this trial—ie, a 250 _L dose containing 25 _g of RTS,S antigen in 250 _L AS02A adjuvant. Because routine hepatitis B vaccination was introduced into the EPI schedule of Mozambique in July, 2001, children aged 12–24 months had already received hepatitis B immunisation. Accordingly, children younger than 24 months received as control vaccines two doses of the seven-valent pneumococcal conjugate vaccine (Prevnar Wyeth Lederle Vaccines, Madison, NJ, USA) at the first and third vaccination and one dose of *Haemophilus influenzae* type b vaccine (GSK Biologicals) at the second vaccination. For children older than 24 months, the control vaccine was the paediatric hepatitis B vaccine (GSK Biologicals). We administered both RTS,S/AS02A and control vaccines intramuscularly in the deltoid region of alternating arms according to a 0, 1, 2 month vaccination Schedule (Alonso PL 2004). | Materials and methods (Study design, Procedures) |
| Objectives | 5 | To provide more evidence on the factors that may influence vaccine response and its duration, we performed a sub-analysis of cohort 2 data that was not included in the original protocol, to estimate vaccine efficacy for clinical malaria in this cohort, using information collected for safety through the health facility-based PCD system. | Abstract, Introduction |
| Outcomes | 6 | In this exploratory analyses, not included in the original protocol, the endpoints were the following:  -first or only clinical malaria episode (Axillary temperature ≥ 37.5ºC + *P. falciparum* asexual parasitaemia >2500 parasites/µl) detected by passive case detection or active detection of infection over a 6 month observation period starting 14 days after dose 3 (study months 2.5 to 8.5) in children in Cohort 2.  -first or only clinical malaria episode (Axillary temperature ≥ 37.5ºC + *P. falciparum* asexual parasitaemia >2500 parasites/µl) detected by passive case detection or active detection of infection over a 12 month observation period starting 6.5 months after dose 3 (study months 8.5 to 21) in children in Cohort 2.  -first or only clinical malaria episode (Axillary temperature ≥ 37.5ºC + *P. falciparum* asexual parasitaemia >15000 parasites/µl) detected by passive case detection or active detection of infection over a 6 month observation period starting 14 days after dose 3 (study months 2.5 to 8.5) in children in Cohort 2.  -first or only clinical malaria episode (Axillary temperature ≥ 37.5ºC + *P. falciparum* asexual parasitaemia >50000 parasites/µl) detected by passive case detection or active detection of infection over a 6 month observation period starting 14 days after dose 3 (study months 2.5 to 8.5) in children in Cohort 2.  -total number of clinical malaria episodes (Axillary temperature ≥ 37.5ºC + *P. falciparum* asexual parasitaemia >2500 parasites/µl) detected by passive case detection or active detection of infection over a 6 month observation period starting 14 days after dose 3 (study months 2.5 to 8.5) in children in Cohort 2.  -first or only clinical malaria episode (Axillary temperature ≥ 37.5ºC + *P. falciparum* asexual parasitaemia >2500 parasites/µl) detected by passive case detection or active detection of infection over a 3.5 month observation period starting 14 days after dose 3 (study months 2.5 to 6) in children in Cohort 2.  - The number of asexual stage *falciparum* parasites per µl of blood for each subject at first or only infection.  - Titres of anti-CS antibodies 30 days post-dose 3. | Materials and methods (Statistical methods and case definitions) |
| Sample size | 7 | The sample size of cohort 2 was calculated at the beginning of the study to estimate vaccine efficacy against first or only infection in cohort 2, which was reported in the primary analysis article: 116 assessable children per group were needed to provide 86% power to detect a vaccine efficacy of 50% in the prevention of new infections with a coger confidence limit of 20%, assuming a rate of new infections of 50% over the surveillance period (Alonso PL 2004).  In this exploratory analyses, based on the incidence of clinical malaria in the control group of cohort 2 (0.944 episodes per person years at risk during the first six months of follow-up and 0.511 during the following 12 months), the power to detect a vaccine efficacy against clinical malaria of 40% or higher at a 5% significance level during the first six months of follow-up is of 65.8% and during the following 12 months is of 68.0%. | Materials and methods (Statistical methods and case definitions) |
| Randomization -- Sequence generation | 8 | Randomization methods are not specified in the current manuscript. They were described in the article reporting the primary analysis:  Block randomisation was done at GSK Biologicals with SAS software version 8 (1/1 ratio, block size six) (Alonso PL 2004). | NA |
| Randomization -- Allocation concealment | 9 | Vaccines (RTS,S/AS02A, Engerix-B™, Prevnar® and Hiberix™) were numbered at GSK according to the randomization list and dispensed to the site in individual boxes labeled with unique study numbers.  The code was released to the investigators once databases had been monitored, cleaned, and locked after completion of follow-up (Alonso PL 2004). | NA |
| Randomization -- Implementation | 10 | Study numbers 0101‑0700 were provided for the children less than 24 months of age and study numbers 0701‑2300 were provided for those children older than 24 months of age. On the day of first vaccination, children were allocated by the vaccination team according to their age, sequentially in order of their arrival, to the next study number. For children of less than 24 months, the numbering started at 0101 and for children of 24 months and over, the numbering started at 0701. | NA |
| Blinding (masking) | 11 | Since the vaccines used were of distinct appearance and volume, special precautions were taken to ensure the double-blind nature of the trial. A vaccination team prepared the vaccine and masked the contents of the syringe with opaque tape before immunisation. This team was not involved in any other study procedures, including surveillance for endpoints (Alonso PL 2004).    Participants, investigators and other study team members assessing the outcomes were blinded to group assignment. | NA |
| Statistical methods | 12 | Vaccine efficacy for first or only clinical malaria episode was assessed using Cox regression models and defined as (1-Hazard Ratio). Vaccine efficacy was adjusted for the covariates: bednet use, geographical area and distance from the health centre (as determined by geopositioning of every household with a handheld global positioning system with differential correction). The proportional hazards assumption was investigated graphically, using a test based on the Schoenfeld residuals and time-dependent Cox models using interactions between the vaccine effect and one-degree fractional polynomials of the time.  For multiple episodes of clinical malaria the vaccine effect was assessed using Poisson regression models with normal random intercepts, including the time at risk as an offset variable. Vaccine efficacy was defined as (1-Rate Ratio). The difference in the geometric mean of the positive densities was assessed with the non-parametric Wilcoxon test.  Analyses were done using STATA version 10.0 (College Station, TX, USA). | Materials and methods (Statistical methods and case definitions) |
| RESULTS Participant flow | 13 | The trial profile for cohort 2 was reported in the primary analysis article (Alonso PL 2004): | NA |
| Recruitment | 14 | Dates are not specified in the current manuscript, but were indicated in the original trial article (Alonso PL 2004).  Screening of participants started in April, 2003, vaccinations took place between July and September and participants were followed-up for six-months starting 14 days after the third dose and for 12 months more months thereafter, until April, 2005. | NA |
| Baseline data | 15 | Baseline characteristics were reported in the article presenting the primary analysis (Alonso PL 2004): | NA |
| Numbers analyzed | 16 | Analyses were performed on the according-to-protocol (ATP) cohort, which was defined as children who met all eligibility criteria, received the complete vaccination course and contributed to the efficacy surveillance. Time at risk started 14 days after dose 3, absences from the study area of two or more weeks and a time interval after antimalarial drug use were not included in the time at risk.  Results of the intention-to-treat (ITT) cohort are also presented. All children who received at least one vaccine dose were included and efficacy estimates were not adjusted for covariates. Time at risk started from dose 1 and was not adjusted for absences from the study area or antimalarial drug use.  For the time at risk in the ATP cohort see table below. | 7-9, 21-22, 29 |
| Outcomes and estimation | 17 | See table below. | 7-9, 29 |
| Ancillary analyses | 18 | The analyses presented in the current manuscript is itself an exploratory analysis of the original trial that has already been published (Alonso PL 2004). | NA |
| Adverse events | 19 | Adverse events are not reported in the current manuscript. These were reported in the two articles reporting the primary analyses for the first six months and subsequent 12 months of follow-up respectively:    Local and general solicited adverse events were of short duration and were mostly mild or moderate in intensity. Grade 3 local or general adverse events were uncommon and of short duration. Local injection-site pain that limited arm motion arose after seven (0·2%) doses in the RTS,S/AS02A group and after one (0·03%) dose in the control vaccine group, and injection-site swelling of more than 20 mm happened after 224 (7·7%) and 14 (0·5%) doses, respectively. General solicited adverse events (fever, irritability, drowsiness, anorexia) that prevented normal activities arose alter 55 (1·9%) doses in the RTS,S/AS02A group and 23 (0·8%) doses in the control group. At least one unsolicited adverse event was reported by 653 (64·5%) children in the RTS,S/AS02A group and 597 (59·1%) in the control group. Safety laboratory values remained unchanged from baseline over the course of the trial. 429 serious adverse events were reported: 180 (17·8%) in the RTS,S/AS02A group and 249 (24·7%) in the control group. 15 children died during the study, five (0·6%) in the RTS,S/AS02A group and ten (1·2%) in the control group. Four of those who died had malaria as a significant contributing factor and all four were in the control group. No serious adverse event or death was judged to be related to vaccination (Alonso PL 2004).  Vaccine safety during the single-blind phase (ITT(8·5–21)) was similar to that previously reported for the double blind phase. 111 serious adverse events were reported among 5·1% (50 of 974) of the participants in the RTS,S/AS02A group versus 138 among 6·8% (66 of 965) of controls. Most of these events were common childhood diseases in Mozambique. During this time there were eight deaths; five in the RTS,S/AS02A group and three in the control group. Two of these deaths were judged to be related to malaria and both occurred in the RTS,S/AS02A group. No serious adverse event or death was judged to be related to vaccination. Assessment of biochemical and haematological variables showed no imbalance between the RTS,S/AS02A and comparator groups (data not shown) (Alonso 2005). | NA |
| *DISCUSSION* Interpretation | 20 | In cohort 2, efficacy of the RTS,S/AS02A candidate malaria vaccine against first or only clinical malaria episodes in Mozambican children aged 1 to 4 years was of 35.4% during the first six months of follow-up, decreasing to 9.0% in the subsequent 12 months of follow-up, although the estimate for the single-blind phase was not significant. The follow up of cohort 2 participants, which included both ADI and PCD, was designed to estimate vaccine efficacy against *P. falciparum* infection and the sample size calculation was based on this endpoint, yielding a size much smaller than for cohort 1. Nevertheless, a high incidence of clinical malaria episodes also allowed estimating vaccine efficacy against this endpoint.  The primary case definition used (fever and a parasitaemia > 2500 parasites/µL) was chosen for cohort 1 based on previous background data from the Manhiça area, where it had been estimated to be 91% specific and 95% sensitive. Using the actual data from cohort 1 this case definition was 95% specific and 86% sensitive (data not shown). This definition had a lower specificity in cohort 2 (72.3%), as malaria transmission in Ilha Josina is higher and children were more immune. Using definitions with higher specificities yielded higher vaccine efficacy estimates.  Efficacy estimates for clinical malaria in cohort 2 are much lower in the intention-to-treat analysis, starting at dose 1 and including the vaccination period, than in the according-to-protocol analysis, starting post dose 3. In the intention-to-treat analysis time at risk is larger and the number of clinical episodes during these first 2.5 months is small, due in part to the administration of anti-malarial treatment before dose 3, diluting the total incidences of the double-blind phase and the differences between RTS,S/AS02A recipients and controls.  The vaccine efficacy against first or only infection in cohort 2, assessed for the double-blind phase and previously published, was of 45.0% (31.4 – 55.9; p<0.0001), an estimate higher than that for the clinical malaria episodes using the primary case definition, but similar to that using fever and parasitaemia >50 000/µL .  The efficacy estimate against clinical malaria in cohort 2 is comparable to that in cohort 1, previously published, that was of 29.9% (95% CI 11.0-44.8; p=0.004) for the double-blind phase. Interestingly, in cohort 1 efficacy did not wane over time and the vaccine was still efficacious at the end of the single-blind phase, whereas in cohort 2 the evaluation of the proportionality of the hazard assumption reveals that the efficacy changes with time.  In summary, cohort 2 children that received RTS,S/AS02A were partially protected against infection and clinical disease in the first six months of follow up post dose 3, but thereafter there was no difference in the risk of malaria between RTS,S/AS02A and control recipients. This is in sharp contrast to study participants in cohort 1, where vaccine efficacy remained stable, with no evidence of waning for at least 21 months.  What are the main differences between cohort 1 and cohort 2 that might explain the discrepancy in vaccine duration of protection? Firstly, the different study design and follow up of participants. In cohort 1 no antimalarial treatment was given to clear parasitaemia before dose 3 and only health facility-based PCD was conducted to detect malaria cases. Therefore those children who became infected with *P. falciparum* had, on average, longer periods of low density parasitaemia, as they were only treated when parasite density reached the fever threshold and the child was taken to the health facility for diagnosis and treatment. Geometric mean parasitaemias (GMPs) at the time of presentation with a clinical malaria episode in cohort 1 were 43522 for the RTS,S/AS02A and 41878 parasites/µL for the control group. On the other hand, in cohort 2 antimalarials were given two weeks before dose 3 and ADI was conducted for six months, during which all children with parasitaemia were treated irrespective of symptoms. In cohort 2 the GMPs were similar (3950 parasites/µL in the RTS,S/AS02A group and 3016 in the control group) at first infection and treatment, but significantly lower in those that were asymptomatic than in those with fever (1815 vs 29402 parasites/µL).  Secondly, the malaria transmission intensity was higher in Ilha Josina, the area where cohort 2 was recruited, as reflected by the geometric mean of antibodies against the whole parasite measured by IFAT and the percentage of splenomegaly at baseline. Therefore participants in Cohort 2 may have had a higher level of naturally acquired immunity against infection and clinical malaria when the trial started.  To understand the effect of vaccination and duration of protection, it is difficult to disentangle the effects of the study design from other potential factors such as the immunity level at the time of vaccination and the malaria exposure. However one potential hypothesis is that, as the vaccine-induced pre-erythrocytic immunity declines following the peak levels achieved after vaccination, it only partially inhibits hepatocyte invasion, liver-stage development and release of merozoites to the blood, decreasing the parasite load in the face of a new infection. A low-dose parasitaemia resulting from the partial pre-erythrocytic immunity might be critical to induce or boost the development of asexual blood-stage immune responses, which may confer long-lasting protection against clinical malaria. To be able to build up this response two factors are needed. Firstly new infections have to occur during the post-vaccination period, during which pre-erythrocytic vaccine-induced immunity will decrease the parasite load. Secondly this low-dose parasitaemia has to be maintained for enough time to stimulate the asexual-stage immune response. Therefore the short-lived vaccine-induced pre-erythrocytic response facilitates the development of a long-lasting asexual-stage immunity in the presence of new infections that act as natural asexual-stage boosters.  This is consistent with other recent hypothesis and studies that assessed the capacity of low parasitaemias to induce protective immune responses. Similar mechanisms have also been proposed to explain the sustained protection during the second year of life of intermittent preventive treatment with sulphadoxine-pyrimethamine administered in infancy (IPTi) in Tanzanian children. Schellenberg *et al.* hypothesized that a drug with a greater parasitocidal effectiveness used as IPTi might induce a shorter protection than other less effective drugs. The same could be argued for pre-erythrocytic malaria vaccines. A vaccine inducing partial pre-erythrocytic protection, like RTS,S/AS, might allow the development of a better and more sustained asexual-stage protection than a more efficacious vaccine, by allowing a “leakage” of low-dose parasites into the blood.  The future deployment of malaria vaccines is envisaged in the context of integrated malaria control, especially insecticide-treated nets (ITNs) and indoor residual spraying. The administration of pre-erythrocytic vaccines together with the use of ITNs, that might decrease the infective dose of sporozoites, could have a synergistic effect, increasing the vaccine efficacy when deployed together.  Children in cohort 1 were probably exposed to low-density parasitaemias for a longer time before developing symptoms during the period after the third vaccine dose, leading to the boosting of the asexual-stage immune response. On the other hand, because children in cohort 2 were free of parasites when they received the last vaccine dose –they had all received an antimalarial treatment before dose 3- and were then rapidly treated for any new infections during the ADI, their immune system was not exposed to low-density asymptomatic parasitaemias for very long and this may have impaired the development of this enhanced asexual-stage immune response. This may explain a waning of the vaccine-specific protective response in cohort 2.  Furthermore, age at the time of vaccination and previous immunity to malaria may also play a role in the vaccine response. During the first three months of follow-up after the third vaccine dose the efficacy of RTS,S/AS02A was between 40 and 70% for all trials (infants, cohort 1 (data not shown) and cohort 2 of children aged 1 to 4 years), irrespective of age or transmission intensity, when using a highly specific malaria case definition. The initial response to the RTS,S/AS02A does not seem to vary in the different trials, what appears to change is the ability to induce a long-lasting protective response. In young children who have not yet developed a high level of acquired immunity, maintained low-dose parasitaemia can induce and boost the asexual-stage immune response. Age at the time of vaccination might be important in achieving a long-lasting protection. It remains to be seen whether the protection in the infant trial is also long-lasting, although, apart from age, other factors including the active detection of infection conducted in that trial might also influence the duration of protection.  The study design of the Phase I/IIb infant trial recently conducted in the same area, in which infants were followed up for 3.5 months, with the same ADI and health facility-based PCD system, was very similar to that of cohort 2. Vaccine efficacy estimated for the first 3.5 months of follow up in cohort 2 children was similar to the efficacy reported in the infant trial. Several differences between the two studies, including age at the time of vaccination, presence of maternally-transferred antibodies, use of ITNs by all participants, and a lower incidence of first or only episodes of parasitaemia in the infant trial (2.7 in the infant trial versus 6.4 per PYAR in cohort 2 in controls), did not seem to have a major influence on the efficacy results for the first months of follow-up.  When designing future pre-erythrocytic malaria vaccine trials it has to be taken into account that the study design might have a great impact on the duration of protection. ADI with rapid treatment of parasitaemias might impair the development of long-lasting protection, although initial efficacy seems to be independent of study design, age or malaria transmission intensity. If assessment of duration of protection is included in the trial objectives the design should consist only of a PCD follow up. Otherwise, if the main aim is to obtain quick efficacy results, a smaller trial with *P. falciparum* infection as the primary endpoint and ADI can be used.  With regard to antibody responses to the vaccine in cohort 2 children, the level of anti-CSP IgG was correlated with a lower risk of infection but not with a lower risk of clinical malaria. In cohort 1 it had not been correlated with the risk of clinical malaria either and in the infant trial it had also been correlated with a lower risk of infection. In addition, antibody levels decayed over the double-blind phase, but remained at the end of the single-blind phase levels 40 fold higher than in controls. This indicates that anti-CSP antibodies, probably together with other cellular immune responses, may be involved in the initial protection and are correlated with protection against infection, supporting the above-mentioned hypothesis. Nevertheless, other unknown immune mechanisms, most likely involving priming of asexual-stage humoral and cellular immunity, developed as the pre-erythrocytic immunity decays, may be responsible for the long-lasting protection against disease. This points towards the need to assess humoral and cellular asexual-stage immune responses in future phase III RTS,S/AS vaccine trials. | 10-16 |
| Generalizability | 21 |  |  |
| Overall evidence | 22 | In conclusion, the preponderance of data discussed leads to the following hypothesis: that the long-term protection against clinical disease observed in RTS,S/AS02A recipients is a consequence of a partially protective vaccine-induced pre-erythrocytic protection that lasts several months, and limits the number of viable sporozoites and merozoites emerging from the liver to initiate the blood stage cycle of the infection. This leads to prolonged exposure to low-dose asexual blood-stage parasites that allows the acquisition of long-lasting asexual blood-stage immunity. Vaccination in infancy therefore has the potential to confer important levels of protection through a time of high susceptibility in early childhood. A fuller understanding of the mechanism of vaccine action including determination of the efficacy and duration under varying conditions of malaria transmission will be reached through the conduct of properly designed phase 3 trials. |  |

|  | **Control vaccine** | | | **RTS,S/AS02A** | | | **Vaccine efficacy***  **(95% CI)** | **p^#^** |
| --- | --- | --- | --- | --- | --- | --- | --- | --- |
| **Endpoint and follow-up period** | **Events** | **PYAR^§^** | **Rate** | **Events** | **PYAR^§^** | **Rate** |
| **Double-blind phase (study months 2.5-8.5)** | | | | | | | | |
| First or only episode of fever and parasitaemia >2500/µL | 56 | 59.3 | 0.94 | 46 | 72.8 | 0.63 | 35.4% (4.5; 56.3) | 0.029 |
| First or only episode of fever and parasitaemia >15 000/µL | 47 | 61.1 | 0.77 | 41 | 73.8 | 0.56 | 30.5% (-5.7; 54.4) | 0.089 |
| First or only episode of fever and parasitaemia >50 000/µL | 33 | 64.4 | 0.51 | 23 | 76.4 | 0.30 | 41.8% (0.8; 65.8) | 0.046 |
| Multiple episodes of fever and parasitaemia >2500/µL | 68 | 70.32 | 0.97 | 52 | 79.2 | 0.66 | 30.0% (-1.8; 51.9) | 0.062 |
| **Single-blind phase (study months 8.5-21)** | | | | | | | | |
| First or only episode of fever and parasitaemia >2500/µL | 59 | 115.4 | 0.51 | 60 | 123.1 | 0.49 | 9.0% (-30.6; 36.6) | 0.609 |

^§^Person-years at risk

^#^p-value from Cox regression model using Wald test
*Treatment effect adjusted by: Age at dose 1, Bednet use at baseline, Distance to health centre (Km)

**References**

Alonso PL, Sacarlal J., Aponte J. J., et al. Efficacy of the RTS,S/AS02A vaccine against *Plasmodium falciparum* infection and disease in young African children: randomised controlled trial. Lancet 2004; 364(9443): 1411-20.

Alonso PL, Sacarlal J, Aponte JJ, et al. Duration of protection with RTS,S/AS02A malaria vaccine in prevention of Plasmodium falciparum disease in Mozambican children: single-blind extended follow-up of a randomised controlled trial. Lancet. 2005; 366(9502): 2012-8.
