# Supplementary material for: Insights into Long-Lasting Protection Induced by RTS,S/AS02A Malaria Vaccine: Further Results from a Phase IIb Trial in Mozambican Children
Source: PLoS One. 2009 Apr 14;4(4):e5165. doi: 10.1371/journal.pone.0005165 (PMC2666156; doi:10.1371/journal.pone.0005165)
Supplement: Protocol S1 — Trial Protocol (1.19 MB DOC) [file pone.0005165.s002.doc]

***Protocol Amendment Approval***

***Study Drug***

GlaxoSmithKline Biologicals' candidate *Plasmodium falciparum* malaria RTS,S/AS02 vaccine

***Protocol Number***

257049/026 (Malaria-026)

| Protocol Title: A double-blind randomised controlled phase IIb study to evaluate the safety, immunogenicity and efficacy of GlaxoSmithKline Biologicals' candidate malaria vaccine RTS,S/AS02A, administered IM according to a 0, 1 and 2 month vaccination schedule in toddlers and children aged 1 to 4 years in a malaria-endemic region of Mozambique. |
| --- |

**Final version of protocol: May 8, 2002 (Final version)**

**Amendment 1: August 13, 2002 (Final version)**

**Amendment 2:** **January 17, 2003 (****Final version)**

| **Coordinating Author: Nicholas Perombelon**  **Rationale/background for changes:**   1. Change in age cut-off for control vaccine regimes   In agreement with the Principal Investigator, vaccines were selected for the control group that were of benefit to the study children (the rationale for the choice of control vaccines is explained in section 1.2.2.4).  Hepatitis B vaccination (at 2, 3 and 4 months of age in combination with diphtheria-tetanus-pertussis and polio vaccines) was introduced into the EPI schedule of Mozambique in July 2001. It is estimated that 83% of children have received all immunizations by 12 months of age (personal communication from Pedro Alonso). Re-immunization with a hepatitis B vaccine would not provide any individual benefit to study children therefore two different vaccination regimes were planned. With a proposed study start in November 2002, it was previously envisaged that only children under the age of 18 months at first vaccination would have received immunisation against hepatitis B as part of their EPI. Therefore, the selected vaccination regimes for the control group were dependent on the age of the child at first vaccination:   - 3 doses of hepatitis B vaccine (Engerix‑B™) to children of 18 months and older. - 2 doses of a 7-valent pneumococcal conjugate vaccine (Prevnar®, Wyeth Lederle Vaccines) at first and third vaccination and 1 dose of Haemophilus influenzae type b vaccine (Hiberix™) at second vaccination to children less than 18 months.   There has been a delay to the start of the study. As a consequence, the proportion of children within the study population that have previously received hepatitis B vaccination will increase: it is now anticipated that children of up to 24 months of age approximately will have received hepatitis B vaccination as part of their EPI, by study start. To ensure that these children benefit from their control vaccine regime, the age range for the control vaccination regime of two doses of Prevnar® and one dose of Hiberix™ will be extended from children less than 18 months to children less than 24 months.  The following sections will be amended: Synopsis, 1.2.2.4, 3, 5.2.2, 5.2.12, 6.1.2, 6.1.3, 6.1.4, 6.4, 6.6, 10.6.2 and Appendix G.  The exclusion criterion for previous hepatitis B vaccination will now be amended to exclude children of 24 months of age and above that have previously received a hepatitis B vaccination (see section 4.4).  This amendment to the vaccination regimes will not affect the vaccination schedules for Prevnar® and Hiberix™. The vaccination schedules for both vaccines comply with the manufacturer’s instructions for children of 12 to 24 months (two doses of Prevnar® at least 2 months apart and one dose of Hiberix™). | | | | |
| --- | --- | --- | --- | --- |
| 1. The change in the cut-off age for the two control vaccination regimes will impact on the study design for Cohort 2. One of the objectives of this cohort is to compare the humoral immune response to the HBsAg of RTS,S/AS02A to Engerix-B™, using a non-inferiority approach (see section 10.6.2). For this analysis, we assumed that, of the 356 children in this cohort, 248 evaluable children of 18 months or more will have received either RTS,S/AS02A or Engerix-B™.   Changing the age range for the control vaccination regime of two doses of Prevnar® and one dose of Hiberix™ from up to 18 months to up to 24 months will result in a decrease in the sample size for the comparison of immunogenicity of RTS,S/AS02A and Engerix-B™. We therefore propose to increase the sample size in Cohort 2 to retain the power for the non-inferiority criteria for seroprotection and geometric mean titres of anti-HBs antibodies. By increasing the sample size in Cohort 2 from 356 children to 416 children (60 more children), there should be the same number of evaluable children that will have received either RTS,S/AS02A or Engerix-B™ for the comparison of the immunogenicity of the two vaccines (see table below). | | | | |
|  | | | | |
|  | Total sample size (Cohort 2) | No. of evaluable subjects* | No of evaluable subjects for non-inferiority comparison** | Sample size  per group |
| Current | 356 | 284 | 248 | 124 |
| Proposed | 416 | 332 | 248 | 124 |
| *Sample size allowing for 20% loss to follow-up  ******Number of subjects of 24 months or more receiving RTS,S/AS02A or Engerix-B™ | | | | |
| The change in the number of children in Cohort 2 is specified in the following sections: Synopsis, 1.2.2.6, 3, 5.8.4, 6.4, 6.6, 10.6.2 and Appendix G.   1. The study protocol has been reviewed and approved by the FDA under an IND application. However, the FDA made some comments on the document and the following changes are proposed in response to these comments:  - Correction of control vaccination regime in the Rationale section of the Protocol Synopsis - Inclusion of a procedure to ensure seroprotection against hepatitis B infection of children receiving RTS,S/AS02A at study end, if non-inferiority of the humoral immune response to the HBsAg of RTS,S/AS02A is not demonstrated. Children receiving RTS,S/AS02A and 24 months or more old at first vaccination will be offered, at the end of the study, the option of testing to see whether they are seroprotected against hepatitis B infection. If not, they will be offered vaccination with Engerix-B™, under the responsibility of the investigator (see section 5.2.12). - Review of blinded safety and reactogenicity data of a subset of the study population by the DSMB after each dose of vaccine to allow the DSMB to make a recommendation for the administration of the subsequent dose of vaccine (see sections 5.1.3.2 and 5.2.4). - Correction of the algorithm for sample size program included in section 10.6.1 and clarification of base used for log transformations (see sections 10.6.2 and 10.7.4). | | | | |
| - Amendment of the methodology for assessment of the non-inferiority of RTS,S/AS02A against Engerix-B™ to replace the a lower limit of a 90% two-sided confidence interval with the lower limit of a 95% two-sided confidence interval, as recommended by the FDA (see section 10.7.4). - Adjustment of the sample size for the non-inferiority comparison of RTS,S/AS02A against Engerix-B™ for a one-sided alpha of 0.025 instead of 0.05 (see section 10.6.2). - Addition of the period from day 0 (first vaccination) to month 21 to the intention to treat analysis of vaccine efficacy (sections 10.7.2.1 and 10.7.2.2.9).  1. In addition, the principal investigator (PI) has requested two changes to the study protocol:  - The screening period will be extended from the current 35 days prior to first vaccination to 90 days prior to first vaccination (Synopsis and sections 3, 5.3, 5.4, 5.5, 5.6 and 5.8.4). - The measurement of spleen size, body weight and height or length will be performed at screening (Clinic visit 1) and not on day 0 (Clinic visit 2), for both cohorts, for logistical reasons. The measurement of body weight and height or length at screening will facilitate the determination of the exclusion criterion for severe malnutrition (sections 5.3, 5.4, 5.5 and 5.6).  1. The DSMB commented, in their review of the study protocol, on the inappropriateness of the requirement for reporting congenital abnormalities in offspring after cessation of the study, in view of the characteristics of the study population (children of 1 to 4 years of age). This part of the protocol has been revised to provide clearer guidance for reporting serious adverse events after study conclusion (see section 8.5.2). 2. For parasite genotyping analysis, in Appendix F it is stated that each time a malaria smear is obtained, whether for routine collections or to evaluate clinical symptoms, a few drops of blood will be collected onto filter paper for analysis.  The collection period for blood samples for parasite genotyping analysis was omitted from the text in Appendix F. The appendix now states that filter paper should be collected for the surveillance period for primary and secondary endpoints (day 90 up to and including day 256).  To be consistent, the text in section 1.2.2.2 has been corrected to remove the specification stating which parasites will be characterised. Clinic visit 4 (day 60) in Cohort 2 does not specify that whole blood will be collected onto filter paper for parasite genotyping analysis even though a finger prick blood sample will be taken for a blood film for parasitaemia determination. This inconsistency has been corrected and whole blood will now be taken on filter paper at Clinic visit 4 in Cohort 2 (see sections 5.5, 5.6 and 5.8.3). This will not entail any additional blood sampling from subjects. There will be sufficient blood taken for the blood film to prepare the blood spot necessary for the analysis of the parasite genotype. These filter paper samples will be stored and analysed as the other samples taken for parasite genotyping in Cohort 1 and 2. 3. The contact details of the local GSK monitor and the Study Monitor have been changed to reflect current appointments (see Title pages, section 8.5.2 and Appendices D and E). 4. By omission, the Screening sampling time point was not specified in Appendix G (Vaccine Supplies, Packaging and Accountability). This has been corrected. | | | | |
| **Amended text in *bold italics* has been included in the following sections:**  Title pages (Study Monitor)  Synopsis (Rationale, Study Design and Number of Subjects)  Section 1.2.2.2 Rationale for parasite genotyping  Section 1.2.2.4 Rationale for choice of control vaccines  Section 1.2.2.6 Assessment of vaccine reactogenicity  Section 3 Study Design Overview  Section 4.2 Number of subjects  Section 4.4 Exclusion Criteria For Enrolment  Section 5.1.3.2 Role of the DSMB  Section 5.2.2 Vaccination process  Section 5.2.4 DSMB review of safety and reactogenicity data per vaccine dose  Section 5.2.12 Cross-over immunisation at study end  Section 5.3 Outline of study procedures for Cohort 1  Section 5.4 Detailed description of study stages/visits for Cohort 1  Section 5.5 Outline of study procedures for Cohort 2  Section 5.6 Detailed description of study stages/visits for Cohort 2  Section 5.8.3.2 Laboratory testing for Cohort 2  Section 5.8.4 Serology plan  Section 6.1.2 Hepatitis B vaccine (Engerix-B™)  Section 6.1.3 Haemophilus influenzae type b vaccine (Hiberix™)  Section 6.1.4 7-valent pneumococcal conjugate vaccine (Prevnar®)  Section 6.4 Treatment allocation and randomization  Section 6.6 Replacement of unusable vaccine doses  Section 8.5.2 Reporting serious adverse events  Section 10.6.1 Cohort 1  Section 10.6.2 Cohort 2  Section 10.7.2.1 Calculation of time at risk  Section 10.7.4 Analysis of immunogenicity  Appendix D Handling of Biological Samples Collected by the Investigator  Appendix E: Shipment of Biological Samples  Appendix F Laboratory Assays  Appendix G Vaccine Supplies, Packaging and Accountability | | | | |

| **Amendment 2 of Malaria-026 study protocol (January 17, 2003 Final version)** approved by: | | | |
| --- | --- | --- | --- |
|  |  |  |  |
| Clinical Development Manager |  |  |  |
|  | Amanda Leach |  | dd-mm-yyyy |
|  |  |  |  |
| Manager, Clinical Development: Biostatistics |  |  |  |
|  | Marc Fourneau |  | dd-mm-yyyy |
|  |  |  |  |
|  |  |  |  |
| Principal Investigator |  |  |  |
|  | Pedro Alonso |  | dd-mm-yyyy |
|  |  |  |  |

| **Study vaccine(s)** | GlaxoSmithKline Biologicals' candidate *Plasmodium* *falciparum* malaria vaccine RTS,S/AS02A | | |
| --- | --- | --- | --- |
| **CPMS Protocol No.** | 257049/026 (Malaria-026) | | |
| **IND No.** | Pending | | |
| **Date of approval** | May 8, 2002 (Final version) | | |
| **Amended** | August 13, 2002  January 17, 2003 | | |
| **Title** | A double-blind randomised controlled phase IIb study to evaluate the safety, immunogenicity and efficacy of GlaxoSmithKline Biologicals' candidate malaria vaccine RTS,S/AS02A, administered IM according to a 0, 1 and 2 month vaccination schedule in toddlers and children aged 1 to 4 years in a malaria-endemic region of Mozambique. | | |
| **GlaxoSmithKline Biologicals** | | | |
| **Coordinating author:** | | Nicholas Perombelon, MSc, Scientific Writer | |
| **Other contributing authors:** | | Cornelia Bevilacqua, MSc, Central Study Coordinator  Roma Chilengi, MD, WHO trainee  Steve Fitzpatrick, MSc, Statistician  Amanda Leach, MSc, MRCPCH, Clinical Development Manager  Opokua Ofori-Anyinam, PhD, Clinical Coordinator  Laurence Vigneron, PhD, Central Study Coordination | |
| **Central Medical Monitor** | | Amanda Leach, MSc, MRCPCH, Clinical Development Manager  GlaxoSmithKline Biologicals,  89, Rue de l'Institut, 1330 Rixensart, Belgium  Tel: + 32 2 656.77.88 Fax: + 32 2 656.61.60 | |
| **Centro de Investigação em Saúde de Manhiça (CISM) Hospital Clínic, Barcelona, Spain Ministerio de Saúde, Mozambique Faculdade de Medicina-Universidade Eduardo Mondlane, Mozambique** | | | |
| **Principal Investigator:** | | Pedro Alonso, MD MSc PhD | |
| **Co-investigators:** | | Ricardo Thompson, PhD  Jahit Sacarlal, MD | |
| **Other contributing authors**  (CISM, Hospital Clínic, Barcelona, Ministerio de Saúde, Mozambique, Faculdade de Medicina-Universidade Eduardo Mondlane, Mozambique): | | Clara Menéndez, MD PhD  John Aponte, MD MSc  Eusebio Macete, MD  Caterina Guinovart, MD MSc | |
| **Other contributing authors** (Malaria Vaccine Initiative at Program for Appropriate Technology in Health): | | Regina Rabinovich, MD, MPH  Jessica Milman, MPH | |
| **Study Monitor** | | ***Local GSK Study Monitor*** | |
| **~~Cornelia Bevilacqua~~**~~,~~ **~~MSc~~**  ***Sabine Corachan***  Central Clinical Study Coordinator  GlaxoSmithKline Biologicals  Rue de l'Institut 89, 1330 Rixensart  Belgium  Tel: ~~+ 32 2 656 77 70~~  ***+32 2 656 92 33***  Fax: + 32 2 656 61 60 **(Amended January 17, 2003)** | | ***Laurien Dodsworth***  ***C/o Reliable de Mozambique***  ***C. Postal 1920***  ***Maputo***  ***Mozambique***  ***Tel: +27 824 585 650***  ***Fax: +258 1 451050***  **(Amended January 17, 2003)** | |
| **Local Safety Monitor** | | **Local Safety Monitor Back-up** | |
| Esperança Sevene, MD  Faculdade de Medicina  Universidade Eduardo Mondlane  Tel: +258 8 2303506  Fax: +258 1 425255  E-mail: [esevene@health.uem.mz](mailto:esevene@health.uem.mz) | | Alda do Rosario Elias Mariano, MD  Faculdade de Medicina  Universidade Eduardo Mondlane  Tel. +258 82 328 053  E-mail: brigith@health.uem.mz | |
| **Sponsors** | | | |
| GlaxoSmithKline Biologicals  Rue de l'Institut 89  1330 Rixensart, Belgium | | | Malaria Vaccine Initiative at Program for Appropriate Technology in Health  6290 Montrose Rd, Rockville, MD 20852 USA |

| **Study vaccine(s)** | GlaxoSmithKline Biologicals' candidate *Plasmodium* *falciparum* malaria vaccine RTS,S/AS02A | | |
| --- | --- | --- | --- |
| **CPMS Protocol No.** | 257049/026 (Malaria-026) | | |
| **IND No.** | Pending | | |
| **Date of approval** | May 8, 2002 (Final version) | | |
| **Amended** | August 13, 2002  January 17, 2003 | | |
| **Title** | **A double-blind randomised controlled phase IIb study to evaluate the safety, immunogenicity and efficacy of GlaxoSmithKline Biologicals' candidate malaria vaccine RTS,S/AS02A, administered IM according to a 0, 1 and 2 month vaccination schedule in toddlers and children aged 1 to 4 years in a malaria-endemic region of Mozambique.** | | |
| **~~Approvals~~** | | | |
| **~~Approved at GlaxoSmithKline Biologicals by:~~** | | | |
| ~~Nadia Tornieporth MD, DTM&H~~  ~~Director, Clinical Development~~ | | ~~Signature~~ | ~~Date~~ |
|  | | | |
| **~~I, the undersigned, have reviewed this protocol (Malaria-026), including Appendices and I will conduct the clinical study as described and will adhere to the Ethical and Regulatory Considerations stated. I have read and understood the contents of the Investigator Brochure.~~** | | | |
| ~~Pedro Alonso, MD, MSc, PhD~~  ~~Principal Investigator~~ | | ~~Signature~~ | ~~Date~~ |
| **(Amended August 13, 2002)** | |  |  |

# Synopsis

| Title | A double-blind randomised controlled phase IIb study to evaluate the safety, immunogenicity and efficacy of GlaxoSmithKline Biologicals' candidate malaria vaccine RTS,S/AS02A, administered IM according to a 0, 1 and 2 month vaccination schedule in toddlers and children aged 1 to 4 years in a malaria-endemic region of Mozambique. | |
| --- | --- | --- |
|  |  | |
| **Indication/**  **Study Population** | Children of 1 to 4 years of age, up to but not including their 5^th^ birthday, in Manhiça District, Mozambique. | |
|  |  | |
| **Rationale** | The RTS,S/AS02A candidate malaria vaccine consists of sequences of the circumsporozoite (CS) protein of the malaria parasite and the hepatitis B surface antigen (HBsAg), adjuvanted with AS02A. Safety and immunogenicity has been demonstrated in non-immune adult and semi-immune adult populations and in children of 1-11 years of age. At time of study start, safety and immunogenicity in children of 1-5 and 6-11 years of age will have been evaluated. Protection against infection has been demonstrated first in malaria sporozoite challenge models and more recently in adults living in an endemic country.  This trial will use a two-cohort design to examine the efficacy of the vaccine at two points in the pathogenesis of malaria disease: infection and clinical disease of moderate severity. The primary endpoint of the trial, on which a decision on the future development of the vaccine is based, is clinical malaria disease.  The study is divided into two phases: a double-blind phase during which safety, reactogenicity and immunogenicity following vaccination and primary and secondary efficacy objectives will be assessed. Thereafter, the study will be unblinded, to allow preliminary evaluation of the results, and a second single-blind phase will then continue for surveillance of tertiary objectives, persistence of immune responses and safety.  A double blind randomised controlled design with control vaccines that are considered of benefit to the study population is proposed. Hepatitis B vaccine, to be used for children aged above ~~18~~ ***24*** months of age, is chosen because hepatitis B infection is acquired throughout childhood and is a major public health problem in Mozambique. Children less than ~~18~~ ***24*** months of age will have already been immunised against hepatitis B in infancy at the study start and therefore will receive two doses of a 7-valent pneumococcal conjugate vaccine ***and*** ~~followed by~~ one dose of a *Haemophilus influenzae* type b (Hib) vaccine as control vaccines. Together, the pneumococcus and Hib account for the majority of acute lower respiratory tract infections in children living in developing countries.  A range of secondary efficacy endpoints will examine malaria cases of different degrees of severity and different manifestations of malaria disease, i.e. acute febrile illness and anaemia.  The HBsAg contained in the RTS,S/AS02A vaccine is encoded by the hepatitis B virus S protein gene that is identical to the gene used to express HBsAg antigen in GSK Biologicals’ Engerix-B™ vaccine against hepatitis B. RTS,S/AS02A is being developed as a vaccine that may offer protection against malaria and hepatitis B. Therefore, this study will make a direct comparison between the immunogenicity of a licensed hepatitis B vaccine (Engerix-B™) and RTS,S/AS02A.  The evaluation of vaccine efficacy against clinical disease in this study will be a key milestone in determining the future development of this vaccine. Therefore the sample size has been calculated to ensure that there is precision around the estimate of effect and that if the true vaccine efficacy is 50%, then there is 80% power to detect a lower limit of vaccine efficacy of 15%.  **(Amended January 17, 2003)** | |
|  |  | |
| **Study design** | - Phase IIb study in one centre and two study sites (towns of Manhiça and Ilha Josina). - The necessary number of healthy male and female children aged 1 to 4 years (inclusive) will be screened in order to enrol ***1980*** ~~1920~~ subjects in the study. - Duration per subject: approximately 24 months. - Screening will be performed within ***90*** ~~35~~ days. - Enrolment will be performed within 12 weeks. - Controlled: - 3 doses of hepatitis B vaccine (Engerix‑B™) to children of ***24*** ~~18~~ months and older. - 2 doses of a 7-valent pneumococcal conjugate vaccine (Prevnar®, Wyeth Lederle Vaccines) at first and third vaccination and 1 dose of *Haemophilus influenzae* type b vaccine (Hiberix™) at second vaccination, to children less than ***24*** ~~18~~ months. - 3 doses of vaccine (RTS,S/AS02A or control) will be administered using a 0, 1 and 2 month vaccination schedule. A full dose (0.5 ml) of the control vaccine or a paediatric dose (0.25 ml dose volume) of the RTS,S/AS02A vaccine will be administered at each vaccination. - Blinding*:* - Vaccination and surveillance period (double-blind phase) from month 0 up to the data lock point for primary and secondary objectives (month 8½). - Second surveillance period (single-blind phase) from month 8½ to data lock point for analysis of tertiary objectives (month 21). - Subjects will be randomly assigned in a 1:1 fashion to receive either the RTS,S/AS02A candidate malaria vaccine or one of the control vaccine regimes (according to age). - Two cohorts will evaluate the effect of the malaria candidate vaccine on clinical malaria disease and malaria infection (Cohorts 1 and 2 respectively, with 1564 subjects in Cohort 1 and ***416*** ~~356~~ subjects in Cohort 2). In Cohort 2 where the efficacy of the vaccine against infection will be evaluated, subjects will receive 4 weeks prior to onset of surveillance one dose of sulfadoxine-pyrimethamine and amodiaquine per day for 3 days, so as to clear any parasitaemia. - Self-contained study, i.e. this study will be independent of other studies.   **(Amended January 17, 2003)** | |
|  |  | |
| **Primary Objective** | The primary objective of the study is to assess the efficacy of RTS,S/AS02A candidate malaria vaccine against clinical episodes of *P. falciparum* malaria in children aged 1 to 4 years at first vaccination, over a 6 month surveillance period starting 14 days after dose 3 (day 74), in Cohort 1. | |
|  |  | |
| **Secondary Objectives** | The secondary objectives correspond exclusively to data collected and endpoints during the double-blind phase of the study (months 0 to 8½). | |
| **Secondary Objectives: Cohort 1** | | |
|  | To assess in children (aged 1 to 4 years at first vaccination) the efficacy of RTS,S/AS02A candidate malaria vaccine against:   - Clinical episodes of *P. falciparum* malaria of differing severity over a 6 month surveillance period starting 14 days after dose 3 (day 74). - Anaemia presenting clinically over a 6 month surveillance period starting 14 days after dose 3 (day 74) or detected at cross-sectional survey at 6½ months after dose 3 (month 8½). - Asexual falciparum parasitaemia (prevalence and density) at 6½ months after dose 3 (month 8½). | |
|  | To assess in children (1 to 4 years of age at first vaccination):   - Safety of RTS,S/AS02A malaria vaccine until 6½ months after dose 3 (month 8½). - Reactogenicity of RTS,S/AS02A malaria vaccine from the time of first vaccination until one month after dose 3 (day 90). - Humoral immune response to the circumsporozoite protein (CS) antigen of the RTS,S/AS02A malaria candidate vaccine | |
| **Secondary Objectives: Cohort 2** | | |
|  | To assess in children (1 to 4 years of age at first vaccination) the efficacy of RTS, S/AS02A malaria candidate vaccine in delaying time to first asexual falciparum infection. | |
|  | To assess in children (1 to 4 years of age at first vaccination):   - Safety of RTS,S/AS02A malaria vaccine until 6½ months after dose 3 (month 8½). - Reactogenicity of RTS,S/AS02A malaria vaccine from the time of first vaccination until one month after dose 3 (day 90). - Humoral immune response to the HBs antigen of the RTS,S/AS02A malaria candidate vaccine. - Humoral immune response to the CS antigen of the RTS,S/AS02A malaria candidate vaccine. | |
|  |  | |
| **Tertiary Objectives** | All the tertiary objectives correspond to endpoints and data collected during the single-blind phase of the study (from month 8½ to month 21). | |
| **Tertiary Objectives: Cohort 1** | | |
|  | To assess in children (1 to 4 years of age at first vaccination) the efficacy of RTS,S/AS02A candidate malaria vaccine against:   - Clinical episodes of *P. falciparum* malaria of differing severity over the surveillance period. - Anaemia presenting clinically over the surveillance period or detected at cross-sectional survey at 19 months after dose 3 (month 21). - Asexual *P. falciparum* parasitaemia (prevalence and density) at 19 months after dose 3 (month 21). | |
|  | To assess in children (1 to 4 years of age at first vaccination):   - Safety of the RTS,S/AS02A malaria candidate vaccine during months 8½ to 21. - Humoral immune response to the CS antigen of the RTS,S/AS02A malaria candidate vaccine at 19 months post dose 3 (month 21) | |
| **Tertiary Objectives: Cohort 2** | | |
|  | To assess in children (1 to 4 years of age at first vaccination):   - Safety of the RTS,S/AS02A malaria candidate vaccine during months 8½ to 21. - Humoral immune response to CS antigen of the RTS,S/AS02A malaria candidate vaccine at 19 months post dose 3 (month 21). - Humoral immune response to HBs antigen of the RTS,S/AS02A malaria candidate vaccine at 19 months post dose 3 (month 21). | |
|  |  | |
| **Number of subjects** | The necessary number of children will be screened to enrol 1564 eligible children in the Cohort 1 and ***416*** ~~356~~ eligible children in the Cohort 2.  **(Amended January 17, 2003)** | |
|  |  |  |
| **Clinical case definitions of malaria** | Primary Case Definition for malaria episodes | - The presence of *P.* *falciparum* asexual parasitaemia above 2500 per µl on Giemsa stained thick blood films AND - The presence of fever (axillary temperature ≥ 37.5°C) at the time of presentation AND - Occurring in a child who is unwell and brought for treatment to a healthcare facility. |
|  | Secondary Case Definition 1 | - The presence of *P.* *falciparum* asexual parasitaemia (any level of parasitaemia) on Giemsa stained thick blood films AND - The presence of fever (axillary temperature ≥ 37.5°C) at the time of presentation AND - Occurring in a child who is unwell and brought for treatment |
|  | Secondary Case Definition 2 | - The presence of *P.* *falciparum* asexual parasitaemia (any level of parasitaemia) on Giemsa stained thick blood films AND - History of fever within 24 hours or documented fever (axillary temperature ≥ 37.5°C) at the time of presentation AND - Occurring in a child who is unwell and brought for treatment |
|  | Secondary Case Definition 3 | - The presence of *P.* *falciparum* asexual parasitaemia above 15 000 per µl on Giemsa stained thick blood films AND - The presence of fever (axillary temperature ≥ 37.5°C) at the time of presentation AND - Occurring in a child who is unwell and brought for treatment to a healthcare facility. |
|  | Anaemia Case Definition | - Haematocrit < 25% |
|  |  | |
| **Primary endpoint** | The time to the first clinical episode of symptomatic *P falciparum* malaria (Primary Case Definition for malaria episodes as defined above) detected by passive case detection over a 6 month observation period starting 14 days after dose 3 (day 74) in children in Cohort 1 (1-4 years of age at first vaccination) who have received RTS,S/AS02A malaria vaccine or one of the control vaccine regimes (either hepatitis B vaccine or *Haemophilus influenzae* type b vaccine and 7-valent pneumococcal conjugate vaccine). | |
| **Secondary endpoints: Cohort 1** | | |
|  | Efficacy:   - The time to the first clinical episode of symptomatic *P falciparum* malaria (Secondary Case Definition 1 as defined above) detected by passive case detection over a 6 month observation period starting 14 days after dose 3 (day 74) in children (1-4 years of age at first vaccination) who have received RTS,S/AS02A malaria vaccine or one of the control vaccine schedules (either hepatitis B vaccine or *Haemophilus influenzae* type b vaccine and 7-valent pneumococcal conjugate vaccine). - The time to the first clinical episode of symptomatic *P falciparum* malaria (Secondary Case Definition 2 as defined above) detected by passive case detection over a 6 month observation period starting 14 days after last vaccination (dose 3) in children (1-4 years of age at first vaccination) who have received RTS,S/AS02A malaria vaccine or one of the control vaccine schedules (either hepatitis B vaccine or *Haemophilus influenzae* type b vaccine and 7-valent pneumococcal conjugate vaccine). - The time to the first clinical episode of symptomatic *P falciparum* malaria (Secondary Case Definition 3 as defined above) detected by passive case detection over a 6 month observation period starting 14 days after last vaccination (dose 3) in children (1-4 years of age at first vaccination) who have received RTS,S/AS02A malaria vaccine or one of the control vaccine schedules (either hepatitis B vaccine or *Haemophilus influenzae* type b vaccine and 7-valent pneumococcal conjugate vaccine). - The total number of clinical episodes of symptomatic *P falciparum* malaria (according to the Primary Case Definition for malaria episodes as defined above) over the time at risk during the 6 month surveillance period (months 2½ to month 8½ inclusive). - The time to the first presentation at Manhiça District Hospital with anaemia (Anaemia Case Definition as defined above) over a 6 month observation period starting 14 days after last vaccination (dose 3) in children (1-4 years of age at first vaccination). - Presence of anaemia in children (1-4 years of age at first vaccination) (Anaemia Case Definition as defined above) at 6½ months after dose 3 (month 8½). - The number of asexual stage falciparum parasites per µl of blood for each subject at 6½ months after dose 3 (month 8½). | |
|  | Safety:   - Occurrence of solicited symptoms after each vaccination over a 4-day follow-up period (day of vaccination and 3 subsequent days). - Occurrence of unsolicited symptoms after each vaccination over a 30-day follow-up period (day of vaccination and 29 subsequent days). - Occurrence of serious adverse events from the time of first vaccination (month 0) until 6½ months post dose 3 (month 8½). | |
|  | Immunogenicity:   - Titres of anti-CS antibodies at the following time points for which serology samples are analyzed: day 0, day 90 and month 8½. | |
| **Secondary endpoints: Cohort 2** | | |
|  | Efficacy   - The time to first malaria infection (first recording of infection of asexual stage falciparum parasites detected by the active detection of infection surveillance or passive case detection) over a period starting 14 days after last vaccination and until 6½ months after last vaccination (month 8½).   Safety:   - Occurrence of solicited symptoms after each vaccination over a 4-day follow-up period (day of vaccination and 3 subsequent days). - Occurrence of unsolicited symptoms after each vaccination over a 30-day follow-up period (day of vaccination and 29 subsequent days). - Occurrence of serious adverse events from the time of first vaccination (month 0) until 6½ months post dose 3 (month 8½).   Immunogenicity   - Titres of anti-CS antibodies at the following time points for which serology samples are analyzed: days 0, and 90, month 8½. - Titres of anti-HBs antibodies at the following time points for which serology samples are analyzed: days 0 and 90, month 8½. | |
|  | | |
| **Tertiary endpoints: Cohort 1** | | |
|  | Efficacy:   - The total number of clinical episodes of symptomatic *P falciparum* malaria (according to the Primary Case Definition for malaria as defined above) over the time at risk during the single-blind surveillance period (months 8½to 21). - The total number of clinical episodes of symptomatic *P falciparum* malaria (according to the Primary Case Definition for malaria as defined above) over the time at risk during the complete surveillance period (months 2½ to 21 inclusive). - Presence of anaemia in children (1-4 years of age at first vaccination) (Anaemia Case Definition as defined above) at 19 months after dose 3 (month 21). - The number of asexual stage falciparum parasites per µl of blood for each subject at 19 months after dose 3 (month 21).   Safety:   - Occurrence of serious adverse events during the single-blind surveillance period (from month 8½ to month 21).   Immunogenicity:   - Titres of anti-CS antibodies at the following time point for which serology samples are analysed: month 21. | |
| **Tertiary endpoints: Cohort 2** | | |
|  | Safety:   - Occurrence of serious adverse events during the single-blind surveillance period (from month 8½ to month 21).   Immunogenicity:   - Titres of anti-HBs and anti-CS antibodies at the following time point for which serology samples are analyzed: month 21. | |

# Table of Contents

**Synopsis** [**10**](#__RefHeading___Toc29810408)

Table of Contents [16](#__RefHeading___Toc29810409)

List of abbreviations [21](#__RefHeading___Toc29810410)

Glossary of terms [23](#__RefHeading___Toc29810411)

1 Introduction [25](#__RefHeading___Toc29810412)

1.1 Background [25](#__RefHeading___Toc29810413)

1.1.1 The RTS,S/AS02A candidate malaria vaccine [25](#__RefHeading___Toc29810414)

1.1.2 The AS02A adjuvant [32](#__RefHeading___Toc29810415)

1.1.3 Centro de Investigação em Saude de Manhiça [33](#__RefHeading___Toc29810416)

1.1.4 Manhiça District [33](#__RefHeading___Toc29810417)

1.1.5 Morbidity in the study area [34](#__RefHeading___Toc29810418)

1.1.6 Manhiça District Hospital [35](#__RefHeading___Toc29810419)

1.1.7 Ilha Josina Health Facility [36](#__RefHeading___Toc29810420)

1.1.8 Malaria drug treatment [37](#__RefHeading___Toc29810421)

1.2 Rationale [37](#__RefHeading___Toc29810422)

1.2.1 Feasibility of the vaccine [37](#__RefHeading___Toc29810423)

1.2.1.1 Concurrent protection against Hepatitis B [39](#__RefHeading___Toc29810424)

1.2.2 Rationale for the design of the phase IIb study [39](#__RefHeading___Toc29810425)

1.2.2.1 Rationale for efficacy endpoints [39](#__RefHeading___Toc29810426)

1.2.2.1.1 Clinical disease [40](#__RefHeading___Toc29810427)

1.2.2.1.2 Infection [41](#__RefHeading___Toc29810428)

1.2.2.2 Rationale for parasite genotyping [42](#__RefHeading___Toc29810429)

1.2.2.3 Rationale for sample size for the primary endpoint [43](#__RefHeading___Toc29810430)

1.2.2.4 Rationale for choice of control vaccines [43](#__RefHeading___Toc29810431)

1.2.2.5 Rationale for conducting hepatitis B serology in Cohort 2 [45](#__RefHeading___Toc29810432)

1.2.2.6 Assessment of vaccine reactogenicity [46](#__RefHeading___Toc29810433)

2 Objectives [47](#__RefHeading___Toc29810434)

2.1 Primary objective [47](#__RefHeading___Toc29810435)

2.2 Secondary objectives [47](#__RefHeading___Toc29810436)

2.2.1 Cohort 1 [47](#__RefHeading___Toc29810437)

2.2.2 Cohort 2 [48](#__RefHeading___Toc29810438)

2.3 Tertiary objectives [49](#__RefHeading___Toc29810439)

2.3.1 Cohort 1 [49](#__RefHeading___Toc29810440)

2.3.2 Cohort 2 [50](#__RefHeading___Toc29810441)

3 Study Design Overview [51](#__RefHeading___Toc29810442)

4 Study Site and Population [53](#__RefHeading___Toc29810443)

4.1 Study site [53](#__RefHeading___Toc29810444)

4.2 Number of subjects [53](#__RefHeading___Toc29810445)

4.3 Inclusion criteria [54](#__RefHeading___Toc29810446)

4.4 Exclusion criteria for enrolment [54](#__RefHeading___Toc29810447)

4.5 Elimination criteria during the study [56](#__RefHeading___Toc29810448)

4.6 Contraindications to vaccination [56](#__RefHeading___Toc29810449)

5 Conduct of Study [57](#__RefHeading___Toc29810450)

5.1 Ethics and regulatory considerations [57](#__RefHeading___Toc29810451)

5.1.1 Institutional Review Board/Independent Ethics Committee (IRB/IEC) [57](#__RefHeading___Toc29810452)

5.1.2 Informed consent [58](#__RefHeading___Toc29810453)

5.1.3 Data Safety Monitoring Board (DSMB) [61](#__RefHeading___Toc29810454)

5.1.3.1 Composition of the DSMB [61](#__RefHeading___Toc29810455)

5.1.3.2 Role of the DSMB [61](#__RefHeading___Toc29810456)

5.1.4 Role of Local Safety Monitor [62](#__RefHeading___Toc29810457)

5.1.5 Independent Study Monitors [62](#__RefHeading___Toc29810458)

5.2 General study aspects [62](#__RefHeading___Toc29810459)

5.2.1 Screening and enrollment procedures [63](#__RefHeading___Toc29810460)

5.2.2 Vaccination process [64](#__RefHeading___Toc29810461)

5.2.3 Safety and reactogenicity assessment during 4-day post-vaccination follow-up [66](#__RefHeading___Toc29810462)

5.2.4 DSMB review of safety and reactogenicity data per vaccine dose [67](#__RefHeading___Toc29810463)

5.2.5 Morbidity surveillance (all subjects) [67](#__RefHeading___Toc29810464)

5.2.6 Passive case detection of clinical cases of malaria (all subjects) [68](#__RefHeading___Toc29810465)

5.2.7 Surveillance for malaria infection (Cohort 2) [69](#__RefHeading___Toc29810466)

5.2.8 Monthly home visits to detect SAEs (all subjects) [71](#__RefHeading___Toc29810467)

5.2.9 Cross sectional surveys (all subjects) [71](#__RefHeading___Toc29810468)

5.2.10 Medical treatment for all study subjects during the study period [72](#__RefHeading___Toc29810469)

5.2.11 Management and treatment of malaria in all subjects [72](#__RefHeading___Toc29810470)

5.2.12 Cross-over immunisation at study end [73](#__RefHeading___Toc29810471)

5.3 Outline of study procedures for Cohort 1 [75](#__RefHeading___Toc29810472)

5.4 Detailed description of study stages/visits for Cohort 1 [77](#__RefHeading___Toc29810473)

5.5 Outline of study procedures for Cohort 2 [83](#__RefHeading___Toc29810474)

5.6 Detailed description of study stages/visits for Cohort 2 [85](#__RefHeading___Toc29810475)

5.7 Clinical examination at cross-sectional surveys [91](#__RefHeading___Toc29810476)

5.8 Sample handling and analysis [92](#__RefHeading___Toc29810477)

5.8.1 Collection of blood samples [92](#__RefHeading___Toc29810478)

5.8.2 Treatment and storage of biological samples [92](#__RefHeading___Toc29810479)

5.8.3 Laboratory assays [92](#__RefHeading___Toc29810480)

5.8.3.1 Laboratory testing for Cohort 1 [92](#__RefHeading___Toc29810481)

5.8.3.2 Laboratory testing for Cohort 2 [93](#__RefHeading___Toc29810482)

5.8.4 Serology plan [96](#__RefHeading___Toc29810483)

6 Study Vaccines and Administration [98](#__RefHeading___Toc29810484)

6.1 Study vaccines [98](#__RefHeading___Toc29810485)

6.1.1 RTS,S/AS02A malaria candidate vaccine [98](#__RefHeading___Toc29810486)

6.1.2 Hepatitis B vaccine (Engerix-B™) [98](#__RefHeading___Toc29810487)

6.1.3 Haemophilus influenzae type b vaccine (Hiberix™) [99](#__RefHeading___Toc29810488)

6.1.4 7-valent pneumococcal conjugate vaccine (Prevnar®) [99](#__RefHeading___Toc29810489)

6.2 Dosage and administration [100](#__RefHeading___Toc29810490)

6.3 Storage [101](#__RefHeading___Toc29810491)

6.4 Treatment allocation and randomization [101](#__RefHeading___Toc29810492)

6.5 Method of blinding and breaking the study blind [102](#__RefHeading___Toc29810493)

6.5.1 Breaking of the study blind [103](#__RefHeading___Toc29810494)

6.6 Replacement of unusable vaccine doses [103](#__RefHeading___Toc29810495)

6.7 Packaging [103](#__RefHeading___Toc29810496)

6.8 Vaccine accountability [103](#__RefHeading___Toc29810497)

6.9 Concomitant medication/treatment [104](#__RefHeading___Toc29810498)

7 Health Economics [104](#__RefHeading___Toc29810499)

8 Adverse Events [104](#__RefHeading___Toc29810500)

8.1 Eliciting and documenting adverse events [105](#__RefHeading___Toc29810501)

8.1.1 Solicited adverse events [107](#__RefHeading___Toc29810502)

8.2 Assessment of intensity [108](#__RefHeading___Toc29810503)

8.3 Assessment of causality [109](#__RefHeading___Toc29810504)

8.4 Following-up of adverse events and assessment of outcome [110](#__RefHeading___Toc29810505)

8.5 Serious adverse events [111](#__RefHeading___Toc29810506)

8.5.1 Definition of a serious adverse event [111](#__RefHeading___Toc29810507)

8.5.2 Reporting serious adverse events [112](#__RefHeading___Toc29810508)

8.6 Pregnancy [114](#__RefHeading___Toc29810509)

8.7 Treatment of adverse events [115](#__RefHeading___Toc29810510)

9 Subject Completion and Drop-out [115](#__RefHeading___Toc29810511)

9.1 Definition [115](#__RefHeading___Toc29810512)

9.2 Procedures for handling drop-outs [115](#__RefHeading___Toc29810513)

9.3 Reasons for drop-out [115](#__RefHeading___Toc29810514)

10 Data Evaluation: criteria for evaluation of objectives [116](#__RefHeading___Toc29810515)

10.1 Clinical case definition of malaria [116](#__RefHeading___Toc29810516)

10.2 Primary endpoint [117](#__RefHeading___Toc29810517)

10.3 Secondary endpoints [118](#__RefHeading___Toc29810518)

10.3.1 Cohort 1 [118](#__RefHeading___Toc29810519)

10.3.2 Cohort 2 [119](#__RefHeading___Toc29810520)

10.4 Tertiary Endpoints [120](#__RefHeading___Toc29810521)

10.4.1 Cohort 1 [120](#__RefHeading___Toc29810522)

10.4.2 Cohort 2 [121](#__RefHeading___Toc29810523)

10.5 Study groups/data sets to be evaluated [121](#__RefHeading___Toc29810524)

10.6 Estimated sample size [122](#__RefHeading___Toc29810525)

10.6.1 Cohort 1 [122](#__RefHeading___Toc29810526)

10.6.2 Cohort 2 [125](#__RefHeading___Toc29810527)

10.7 Final analyses [127](#__RefHeading___Toc29810528)

10.7.1 Analysis of demographics [127](#__RefHeading___Toc29810529)

10.7.2 Analysis of efficacy [127](#__RefHeading___Toc29810530)

10.7.2.1 Calculation of time at risk [127](#__RefHeading___Toc29810531)

10.7.2.2 Vaccine efficacy [129](#__RefHeading___Toc29810532)

10.7.2.2.1 Cohort 1: primary and secondary endpoints [129](#__RefHeading___Toc29810533)

10.7.2.2.2 Cohort 1: secondary endpoint for estimation of vaccine efficacy given multiple episodes per subject [129](#__RefHeading___Toc29810534)

10.7.2.2.3 Cohort 1: tertiary endpoints for cumulative incidence [130](#__RefHeading___Toc29810535)

10.7.2.2.4 Parasite density in Cohort 1: secondary and tertiary endpoints [130](#__RefHeading___Toc29810536)

10.7.2.2.5 Analysis of haematocrit values in Cohort 1: secondary and tertiary endpoints [130](#__RefHeading___Toc29810537)

10.7.2.2.6 Cohort 2: secondary endpoint [131](#__RefHeading___Toc29810538)

10.7.2.2.7 Adjustment for covariates [131](#__RefHeading___Toc29810539)

10.7.2.2.8 Comparability between study sites [131](#__RefHeading___Toc29810540)

10.7.2.2.9 Exploratory analyses [132](#__RefHeading___Toc29810541)

10.7.3 Analysis of safety [132](#__RefHeading___Toc29810542)

10.7.4 Analysis of immunogenicity [133](#__RefHeading___Toc29810543)

10.8 Planned interim analysis [135](#__RefHeading___Toc29810544)

11 Administrative Matters [135](#__RefHeading___Toc29810545)

References [136](#__RefHeading___Toc29810546)

CONTENTS: Protocol TABLES

Table 1: Frequency of solicited symptoms per dose during the 4-day follow-up period for each vaccine group (ATP cohort for safety, Malaria-015). [28](#__RefHeading___Toc29810547)

Table 2: Frequency of solicited symptoms per dose during the 4-day follow-up period for each vaccine group (ATP cohort for safety, Malaria-020). [28](#__RefHeading___Toc29810548)

Table 3: Classification of spleen sizes according to Hackett [91](#__RefHeading___Toc29810549)

Table 4: Definitions of symptomatic P. falciparum malaria [117](#__RefHeading___Toc29810550)

Table 5: Sample size to establish the lower limit of the 95% CI of vaccine efficacy in Cohort 1 [124](#__RefHeading___Toc29810551)

Table 6: Sample size to establish the lower limit of the 95% CI of vaccine efficacy in Cohort 2 [125](#__RefHeading___Toc29810552)

Table 7 : Power to achieve non-inferiority criterion for seroprotection and GMT ratio given the availability of seronegative only subjects in the non-inferiority cohort (124 per group) [127](#__RefHeading___Toc29810553)

Appendix A: World Medical Association Declaration of Helsinki

Appendix B: Administrative Matters

Appendix C: Overview of the recruitment plan

Appendix D: Handling of Biological Samples Collected by the Investigator

Appendix E: Shipment of Biological Samples

Appendix F: Laboratory Assays

Appendix G: Vaccine Supplies, Packaging and Accountability

Appendix H: Amendments/Modifications to the Study Protocol

# List of abbreviations

AE Adverse Event

ALRI Acute lower respiratory tract infections

ALT Alanine aminotransferase

anti-CS antibody to the *P. falciparum* circumsporozoite (CS) repeat protein

anti-HBs antibody to the hepatitis B surface antigen

AS02A (3,4) GlaxoSmithKline adjuvant system 2 (3 or 4)

ATP According to protocol

CBC Complete Blood Count

CI Confidence Interval

CISM Centro deInvestigação Em Saude de Manhiça, Mozambique.

CMI Cell Mediated Immunity

CRA Clinical Research Associate

CS Circumsporozoite Protein

CSP Circumsporozoite Repeat Protein

CTL Cytotoxic T-lymphocyte

DSMB Data Safety Monitoring Board

EIR Entomological Inoculation Rate

ELISA Enzyme Linked Immunosorbent Assay

EIA Enzyme Immunosorbent Assay

EPI Expanded Programme on Immunization

GAVI Global Alliance for Vaccines and Immunization

GMT Geometric Mean Titre

GSK GlaxoSmithKline

GSK Biologicals GlaxoSmithKline Biologicals

HB Hepatitis B

HBsAg Hepatitis B surface antigen

HBV Hepatitis B virus

Hib *Haemophilus influenzae* type b

HIV Human Immunodeficiency Virus

HSPC Human Subjects Protection Committee

IFAT Indirect fluorescent antibody test

IM Intramuscular

INDEPTH International Network for the continuous Demographic Evaluation of Populations and their Health

IFN-γ Interferon gamma

IRB/IEC Institutional Review Board/Independent Ethics Committee

kg kilogram

LSM Local Safety Monitor

MedDRA Medical Dictionary for Regulatory Activities

mg milligram

ml millilitre

3D-MPL 3-deacylated monophosphoryl lipid A

MVI Malaria Vaccine Initiative

PATH Program for Appropriate Technology in Health

*P. falciparum* *Plasmodium falciparum*

PBS Phosphate buffered saline

PCR Polymerase Chain Reaction

PFS Pre-Filled Syringe

PRP Polyribosyl-ribitol-phosphate

QC Quality Control

QS 21 “Quillaja Saponaria 21” : a triterpene glycoside purified from the bark of Quillaja Saponaria

R32LR Circumsporozoite protein (CSP)

RAP Report Analysis Plan

RSV Respiratory syncitial virus

RTS Hybrid protein comprising S and CSP portions

RTS,S Particulate antigen, containing both RTS and S proteins

SAE Serious Adverse Event

SB62 Oil in water emulsion

SOP Standard Operating procedure

SP Sulfadoxine-pyrimethamine

SSN Subject study identification number

Th1 T-helper type 1 lymphocytes

WHO World Health Organization

WRAIR Walter Reed Army Institute of Research

# Glossary of terms

| **Subject(s):** | Term used throughout the protocol to denote the enrolled individual(s). |
| --- | --- |
| **Medical Monitor:** | An individual medically qualified to assure the responsibilities of the sponsor especially as regards the ethics, clinical safety of a study and the assessment of adverse events. |
| **Study Monitor:** | An individual assigned by and centrally located at GlaxoSmithKline Biologicals who is responsible for assuring proper conduct of a clinical study. |
| **Site Monitor:** | An individual assigned by GlaxoSmithKline Biologicals who is responsible for assuring proper conduct of a clinical study at one or more investigational sites. |
| **Local Safety Monitor:** | The overall role of the Local Safety Monitor, an experienced physician based in-country, will be to support the study investigators and to act as a link between the investigators and the Data Safety Monitoring Board (DSMB) (see Section 5.1.4 for further details). |
| **Medical Assistants  (Tecnicos de Medicina):** | Patient care personnel (paramedics) at Manhiça District Hospital and other Mozambican health facilities with schooling up to 12^th^ grade (pre-university) followed by two years of medical training. |
| **Medical Agents (Agentes de Medicina):** | Patient care personnel (medical receptionists and replacement nurses) at Manhiça District Hospital and other Mozambican health facilities with schooling up to 9^th^ grade (pre-university) followed by three years of medical training. |
| **Malaria episode:** | In this study we use the term malaria episode as a generic term to describe a clinical case of malaria that encompasses anyone of the more specific case definitions used to determine efficacy endpoints (see section 10.1 for case definitions). |
| **Malaria infection:** | Presence of parasitaemia of any level due to *Plasmodium falciparum* in a subject’s blood sample regardless of the presence or absence of symptomatology associated with clinical disease. |
| **Passive case detection:** | The detection of a malaria episode at Manhiça District Hospital or Ilha Josina Health Facility. |
| **Active detection of infection:** | The detection of malaria parasitaemia on a blood sample taken at predefined time points, i.e. regardless of whether a child has signs or symptoms of infection. |
| **Eligible:** | Qualified for enrolment into the study based upon strict adherence to inclusion/exclusion criteria. |
| **Evaluable:** | Meeting all eligibility criteria, complying with the procedures defined in the protocol, and, therefore, included in analysis (see Sections 4.5 and 10.5 for details on criteria for evaluability). |
| **Protocol amendment:** | Any change in a clinical protocol which affects the safety of subjects, the scope, design, assessments or scientific validity of the clinical investigation, e.g., dose change, duration of treatment, number of subjects, control group(s), the assessments. |
| **Protocol modification:** | Strictly, any change to a clinical protocol, which is not considered to be an amendment, e.g., changes to clarify (but not alter) design features or encourages greater compliance with the intent of the clinical trial protocol. |

# Introduction

## Background

Four species of the *Plasmodium* protozoan parasite are the aetiologic agents of malaria in humans (*P. falciparum,* *P. vivax,* *P. ovale* and *P. malariae*). Of these four parasites, *P. falciparum* is the major cause of severe morbidity and mortality. Among African children under 5 years of age living in endemic areas, there are between 400 and 900 million acute febrile episodes yearly of which about a half are due to malaria[[1]](#endnote-2). At a minimum, between 700 000 and 2.7 million persons die yearly from malaria and over 75% of them are African children. The incidence of malaria in much of Africa is increasing for a variety of reasons - changes in agricultural practices, armed conflicts, migration of refugees, increasing drug resistance to conventional anti-malarial drugs, and insecticide resistance of the Anopheline (mosquito) vectors – and it is estimated that malaria will more than double over the next 20 years without effective control. The burden of malaria at the country level correlates closely with the rate of economic development even after adjustment for confounding factors, indicating that malaria is an important constraint on economic progress[[2]](#endnote-3).

### The RTS,S/AS02A candidate malaria vaccine

GlaxoSmithKline (GSK) Biologicals has developed a candidate vaccine against *P. falciparum* malaria, the RTS,S/AS02A malaria vaccine, which has been shown to be safe and immunogenic in malaria-experienced adult populations in The Gambia and Kenya and malaria-naive adult populations in Belgium and the USA[[3]](#endnote-4),[[4]](#endnote-5). The vaccine is available in freeze-dried form (with a freeze-dried antigen pellet in a vial to be reconstituted with liquid adjuvant AS02A)

The vaccine has been shown to protect between 42% and 86% of healthy non-immune volunteers against infection in homologous sporozoite challenge studies, when given according to a 2 or 3 dose vaccination schedule[[5]](#endnote-6). Protection appears to be of short duration as only one out of five volunteers that were protected in an initial challenge study were protected on rechallenge 6 months after the primary challenge[[6]](#endnote-7).

In a double-blind, randomised, controlled phase IIb study in an endemic region of The Gambia, 250 men received 3 doses of the vaccine and were followed for 15 weeks. Vaccine efficacy was determined as the time to first detection of infection due to *P. falciparum.* Overall efficacy against infection, adjusted for confounders, was 34% (95% confidence interval (CI): 8% to 53%; p = 0.014)[[7]](#endnote-8) but the initially observed protection appeared to wane over time. The estimated efficacy during the first 9 weeks of follow-up was 71% (95% CI: 46% to 85%; p < 0.0005), decreasing to 0% in the last 6 weeks. Subsequently, 158 men received a fourth dose during the peak malaria season of the following year and were followed up for 9 weeks. Vaccine efficacy against infection was 47% (95% CI: 3.8 to 71 % p = 0.037) and a strong boosting of immune responses was induced. Future studies to evaluate vaccine efficacy will have as their primary endpoint the effect of the vaccine to prevent clinical malaria disease (clinical episodes).

Irrespective of study populations (semi-immune or malaria-naive), pain at the injection site (almost all mild to moderate in intensity) has been the most frequently reported local solicited symptom. In malaria-naive populations, the most frequently reported general solicited symptoms causally related to vaccination have been myalgia and fatigue, with a small number of transient severe cases being observed. In the semi-immune population, headache and malaise were the most frequently reported.

Only one serious adverse event was suspected of being causally related to vaccination with RTS,S/AS02A. One of the chronic asymptomatic carriers in Malaria-005 was reported to be clinically jaundiced and have an ALT level of 335 IU/l approximately 2 weeks after dose 2. This adverse event was reported by the investigator as an SAE suspected of being causally related to vaccination with RTS,S/AS02A. During this episode, his viral DNA levels ranged between less than 5 to 250 pg/ml. All symptoms resolved within a period of approximately 2 weeks without treatment and the serum ALT level was normal when tested 8 weeks later (25 IU/l). Dose 3 was not administered to this subject. Six months after the second vaccination, the same subject presented with symptoms of an acute febrile illness and the serum ALT level was 509 IU/l. A presumptive diagnosis of typhoid fever was made by the principal investigator and antibiotic treatment was initiated. All symptoms had resolved and ALT had normalised (25 IU/l) 4 weeks later. In the comparator group of the same study (rabies vaccine control group) four cases of elevated ALT were reported in chronic carriers of the hepatitis B surface antigen. The chronic carriers of the hepatitis B virus and the healthy volunteers showed similar reactogenicity profiles. All reported adverse events resolved without sequelae.

Various vaccination schedules (0,1,5,18 months/0,1,6,13 months/0,1,9 months) have been assessed and all showed the vaccine to be immunogenic with acceptable reactogenicity in adults. There has been no significant increase in reactogenicity with a fourth vaccine dose. Data from a study recently conducted in the USA to evaluate a 0,1,3-month and a 0,7,28-day vaccination schedule in malaria-naive adults indicates that the immune response and efficacy following sporozoite challenge are comparable to that after a 0,1,6-month vaccination schedule[[8]](#endnote-9). No changes in the safety or reactogenicity profile of the vaccine were identified with either schedule.

Following demonstration of the safety, immunogenicity and efficacy of the RTS,S/AS02A vaccine in adults, the candidate vaccine progressed to clinical evaluation in children. In order to ensure maximal safety on first administration of the vaccine antigen RTS,S and the novel adjuvant AS02A in younger age groups, a dose-escalation approach was adopted using 1/5 (0.1 ml), 1/2 (0.25 ml) and full (0.5 ml) doses of RTS,S/AS02A. The vaccine safety and reactogenicity was under close monitoring at each dose level by both the investigator and the local safety monitor. An independent Data Safety Monitoring Board (DSMB) was established to oversee study conduct and fully analyse and review safety and immunogenicity data prior to the start of the current study (Malaria-026). Evaluation of the candidate vaccine in a paediatric population was first conducted in children aged 6-11 years (Malaria-015[[9]](#endnote-10)) with a subsequent study in toddlers and children aged 1-5 years (Malaria-020[[10]](#endnote-11)), both in The Gambia. Each study was double-blinded, randomized and controlled (HDC rabies vaccine, Aventis Pasteur, Lyon) to allow for a more objective assessment of reactogenicity, given the potentially high rate of intercurrent infections in the study populations. The data from these two studies were to aid in the selection of the dose level to be used in the future pediatric development of the RTS,S/AS02A candidate vaccine.

In total, data have been generated following the administration of 60, 59 and 60 doses of 1/5 (0.1 ml), 1/2 (0.25 ml) and full (0.5 ml) RTS,S/AS02A dose volumes respectively to 20 children aged 6-11 years in each dose group, and 89, 89 and 89 doses of 1/5 (0.1 ml), 1/2 (0.25 ml) and full (0.5 ml) RTS,S/AS02A dose volumes respectively to 30 toddlers and children aged 1-5 years in each dose group.

Table 1: Frequency of solicited symptoms per dose during the 4-day follow-up period for each vaccine group (ATP cohort for safety, Malaria-015).

|  |  | **1/5 RTS,S/ AS02** | | **Rabies** | | **1/2 RTS,S/ AS02** | | **Rabies** | | **Full RTS,S/ AS02** | | **Rabies** | |
| --- | --- | --- | --- | --- | --- | --- | --- | --- | --- | --- | --- | --- | --- |
|  |  | **N=60** | | **N=30** | | **N=59** | | **N=30** | | **N=60** | | **N=30** | |
| **Symptom** |  | **n** | **%** | **n** | **%** | **n** | **%** | **n** | **%** | **n** | **%** | **n** | **%** |
| Limited arm | Any | 5 | 8.3 | 1 | 3.3 | 12 | 20.3 | 2 | 6.7 | 11 | 18.3 | 0 | 0.0 |
| motion | Grade 3 | 0 | 0.0 | 0 | 0.0 | 2 | 3.4 | 0 | 0.0 | 2 | 3.3 | 0 | 0.0 |
| Injection site | Any | 53 | 88.3 | 21 | 70.0 | 53 | 89.8 | 27 | 90.0 | 59 | 98.3 | 26 | 86.7 |
| pain | Grade 3 | 0 | 0.0 | 0 | 0.0 | 4 | 6.8 | 0 | 0.0 | 8 | 13.3 | 0 | 0.0 |
| Injection site | Any | 5 | 8.3 | 1 | 3.3 | 10 | 16.9 | 0 | 0.0 | 12 | 20.0 | 0 | 0.0 |
| swelling | Grade 3 | 1 | 1.7 | 0 | 0.0 | 1 | 1.7 | 0 | 0.0 | 4 | 6.7 | 0 | 0.0 |
| Headache | Any | 11 | 18.3 | 4 | 13.3 | 14 | 23.7 | 4 | 13.3 | 12 | 20.0 | 3 | 10.0 |
|  | Grade 3/Rel | 0 | 0.0 | 0 | 0.0 | 0 | 0.0 | 0.0 | 0.0 | 1 | 1.7 | 0 | 0.0 |
| Malaise | Any | 5 | 8.3 | 0 | 0.0 | 8 | 13.6 | 2 | 6.7 | 11 | 18.3 | 2 | 6.7 |
|  | Grade 3/Rel | 0 | 0.0 | 0 | 0.0 | 0 | 0.0 | 0 | 0.0 | 1 | 1.7 | 0 | 0.0 |
| Nausea | Any | 4 | 6.7 | 1 | 3.3 | 2 | 3.4 | 1 | 3.3 | 6 | 10.0 | 0 | 0.0 |
|  | Grade 3/Rel | 0 | 0.0 | 0 | 0.0 | 0 | 0.0 | 0 | 0.0 | 0 | 0.0 | 0 | 0.0 |
| Fever | Any* | 6 | 10.0 | 0 | 0.0 | 5 | 8.5 | 3 | 10.0 | 6 | 10.0 | 2 | 6.7 |
|  | Grade 3/Rel | 0 | 0.0 | 0 | 0.0 | 0 | 0.0 | 0 | 0.0 | 0 | 0.0 | 0 | 0.0 |
| N = number of documented doses, n/% = number/percentage of doses followed by a local/general symptom, *axillary body temperature ≥ 37.5°C. Grade 3: limited arm motion; abduction at the shoulder ≤ 30°, pain at the injection site; spontaneously painful, swelling; > 50 mm and persisting more than 24 hours, fever; axillary temperature ≥ 39.0 °C; for other symptoms; adverse event that prevents normal activity. Rel = related to study vaccine. Note: all local symptoms were considered related to study vaccine. | | | | | | | | | | | | | |

Table 2: Frequency of solicited symptoms per dose during the 4-day follow-up period for each vaccine group (ATP cohort for safety, Malaria-020).

|  |  | **1/5 RTS,S/ AS02** | | **Rabies** | | **1/2 RTS,S/ AS02** | | **Rabies** | | **Full RTS,S/ AS02** | | **Rabies** | |
| --- | --- | --- | --- | --- | --- | --- | --- | --- | --- | --- | --- | --- | --- |
|  |  | **N=89** | | **N=39** | | **N=89** | | **N=45** | | **N=89** | | **N=41** | |
| **Symptom** |  | **n** | **%** | **n** | **%** | **n** | **%** | **n** | **%** | **n** | **%** | **n** | **%** |
| Injection site | Any | 70 | 78.7 | 34 | 87.2 | 75 | 84.3 | 38 | 84.4 | 82 | 92.1 | 32 | 78.0 |
| Pain | Grade 3 | 1 | 1.1 | 0 | 0.0 | 5 | 5.6 | 1 | 2.2 | 13 | 14.6 | 0 | 0.0 |
| Injection site | Any | 5 | 5.6 | 2 | 5.1 | 23 | 25.8 | 2 | 4.4 | 26 | 29.2 | 3 | 7.3 |
| Swelling | Grade 3 | 5 | 5.6 | 1 | 2.6 | 16 | 18.0 | 2 | 4.4 | 24 | 27.0 | 1 | 2.4 |
| Fever | Any* | 10 | 11.2 | 10 | 25.6 | 8 | 9.0 | 9 | 20.0 | 15 | 16.9 | 5 | 12.2 |
|  | Grade 3/Rel | 0 | 0.0 | 1 | 2.6 | 1 | 1.1 | 1 | 2.2 | 0 | 0.0 | 1 | 2.4 |
| Drowsiness | Any | 8 | 9.0 | 2 | 5.1 | 7 | 7.9 | 4 | 8.9 | 5 | 5.6 | 2 | 4.9 |
|  | Grade 3/Rel | 0 | 0.0 | 0 | 0.0 | 0 | 0.0 | 0 | 0.0 | 0 | 0.0 | 0 | 0.0 |
| Loss of | Any | 7 | 7.9 | 1 | 2.6 | 11 | 12.4 | 9 | 20.0 | 13 | 14.6 | 4 | 9.8 |
| Appetite | Grade 3/Rel | 0 | 0.0 | 0 | 0.0 | 2 | 2.2 | 1 | 2.2 | 2 | 2.2 | 0 | 0.0 |
| Irritability/ | Any | 4 | 4.5 | 0 | 0.0 | 9 | 10.1 | 6 | 13.3 | 24 | 27.0 | 5 | 12.2 |
| Fussiness | Grade 3/Rel | 0 | 0.0 | 0 | 0.0 | 0 | 0.0 | 1 | 2.2 | 3 | 3.4 | 0 | 0.0 |
| N = number of documented doses, n/% = number/percentage of doses followed by a local/general symptom, *axillary body temperature ≥ 37.5°C. Grade 3: pain at the injection site; cries when limb is moved/spontaneously painful, swelling; > 20 mm, fever; axillary body temperature ≥ 39.0°C; irritability/fussiness; crying that cannot be comforted/prevents normal activity, drowsiness; drowsiness that prevents normal activity, loss of appetite; not eating at all, for other symptoms; adverse event that prevents normal activity. Rel = related to study vaccine. Note: all local symptoms were considered related to study vaccine. | | | | | | | | | | | | | |

As shown in Table 1 and Table 2, pain at the injection site was a frequent symptom across all study groups. In children aged 6-11 years, injection site pain was reported after 88%, 90% and 98% of doses in the 1/5, ½ and full dose RTS,S/AS02A groups respectively, compared to 70%, 90% and 87% in the three control groups. Similarly, in the younger children, pain was experienced after 79%, 84% and 92% of doses in the 1/5, 1/2 and full dose RTS,S/AS02A groups respectively. This compared to 93%, 100% and 100% of subjects in the three control groups. Grade 3 pain was observed more frequently in the RTS,S,S/AS02A groups than controls. In children aged 6-11 years this occurred after 0%, 7%, 13% of doses in the 1/5, ½ and full RTS,S/AS02A groups, but did not occur in the control groups. In the 1-5 year old children, grade 3 pain was experienced after 1%, 6%, 15% of doses across dose levels compared to 0% in the control groups.

The incidence of swelling increased with dose level. In the 6-11year-old children, it was observed after 8%, 17% and 20% of doses of increasing dose level, compared to 3%, 0% and 0% of doses in the three control groups. In the younger children aged 1-5 years, swelling occurred with 6%, 26% and 29% of doses compared to 5%, 4% and 7% of doses in the three control groups. Swelling of severe (grade 3) intensity (> 20 mm) was observed among the 1-5 year-olds, at a frequency of 6%, 18% and 27% of doses of increasing dose level compared to 3%, 4% and 2% in the control groups. However it should be noted that comparison to the experience in children aged 6-11 years is complicated by the difference in the criteria for grade 3 swelling (> 20 mm for 1-5 year-olds and >50 mm for 6-11 year-olds). All local events graded 3 in intensity resolved or decreased in intensity within 48 hours, with the exception of one symptom of swelling in the full RTS,S/AS02A group in the younger age group, which lasted for 5 days. Local reactogenicity was similar in the children aged 1-5 and 6-11 years.

The majority of general solicited symptoms were mild to moderate in intensity and short lasting. In children aged 6-11 years, headache was the most frequently occurring general solicited symptom in subjects receiving RTS,S/AS02A, occurring after 18%, 24% and 20% of doses of 1/5 (0.1 ml), 1/2 (0.25 ml) and full (0.5 ml) dose of RTS,S/AS02A respectively, compared to 13%, 13% and 10% of doses in the corresponding three control groups. In the younger children, the most frequently occurring general symptom in subjects receiving 1/5 (0.1 ml) dose RTS,S/AS02A was fever, reported after 11% of doses compared to 26% of doses in the rabies control group. In subjects receiving ½ (0.25 ml) dose RTS,S/AS02A, loss of appetite was the most frequent symptom, being reported after 12% of doses, which compared to 20% of doses in the control group. In subjects receiving full dose RTS,S/AS02A irritability/fussiness was most frequently reported, occurring after 27% of doses compared to 12% of doses in the control group. Grade 3 general solicited symptoms were infrequent in both studies and resolved or decreased in intensity within 24 hours.

Unsolicited symptoms were recorded with similar (6-11 year-olds) or lower frequency (1-5 year-olds) in the study vaccine compared to the control vaccine groups. The majority of unsolicited symptoms were mild to moderate in intensity and unrelated to vaccination.

Haematocrit values were generally low but comparable between study populations. Six children in the RTS,S/AS02A groups (one in 1/5 dose group, five in ½ dose group) experienced moderate anaemia, defined as a haematocrit of 15%-24% during the course of the study. There were no cases in the control groups, however, one subject in the rabies group had a low haemoglobin value (8.2 g/dl). All these children had documented malaria episodes before or at the time that anaemia was recorded.

All children were monitored for liver function. Among the 6-11 year-olds, two children in the RTS,S/AS02A groups experienced a transient rise in ALT levels judged not to be related to vaccination and not clinically relevant. In the younger children, increases in ALT levels from pre-vaccination were observed in 2 subjects receiving RTS,S/AS02A and 2 subjects in the rabies groups. Of these, only one case in the rabies control group was judged to be clinically relevant and vaccination was discontinued after the first dose.

No other clinically relevant abnormalities of haematological or biochemical laboratory parameters were observed.

Six serious adverse events (SAEs) in total occurred but all SAEs were considered by the investigator not to be related to study vaccine. Two SAEs were reported in the RTS,S/AS02A groups: acute malaria with acute upper respiratory tract infection 11 days post dose 3 (1/5 RTS,S/AS02A group), acute severe cerebral malaria 54 days post dose 2 (½ RTS,S/AS02A group). Four SAEs were reported in the rabies control groups: acute severe malaria with urinary tract infection and salmonella septicaemia, bronchopneumonia, bronchopneumonia with bronchial asthma and death due to accidental drowning. Apart from the fatal SAE, all subjects made a full recovery and were not withdrawn from the study.

With respect to the pooled humoral immunogenicity results for Malaria-015 and Malaria-020, all doses levels of RTS,S/AS02A (1/5 [0.1 ml], ½ [0.25 ml] and full [0.5 ml] doses) were highly immunogenic for anti-CS and anti-HBs antibodies, irrespective of pre-vaccination HBs serostatus.

All subjects receiving RTS,S/AS02A were seropositive for anti-CS antibodies post dose 2. The lowest anti-CS antibody geometric mean titers (GMTs) occurred in the 1/5 RTS,S/AS02A dose group. In the 1/2 and full RTS,S/AS02A dose groups, similar values were recorded. All subjects in the RTS,S/AS02A groups were seroprotected for anti-HBs antibodies post dose 2 in Malaria-015 and post dose 3 (first post-vaccination assessment) in Malaria-020; the highest GMTs were observed in the 1/2 RTS,S/AS02A dose group in Malaria-015 and in the full RTS,S/AS02A dose group in Malaria-020. Overall, the values observed in this population of children aged 1-11 years were within the ranges seen in previous studies with the RTS,S/AS02A vaccine in malaria-naïve adult subjectsError: Reference source not found.

Following a full review of the pediatric Phase I safety, reactogenicity and immunogenicity data, the DSMB judged that all dose levels of RTS,S/AS02A administered (1/5 [0.1 ml], ½ [0.25 ml] and full [0.5 ml] doses) were safe with acceptable reactogenicity. However, the pooled immunogenicity data were considered to be indicative of an inferior anti-CS and anti-HBs immune response following the administration of the 1/5 (0.1 ml) dose level.

In addition, taking into account previous albeit limited experience with the ½ (0.25 ml) and full (0.5 ml) dose levels in sporozoite challenge studies in adults, which demonstrated protective efficacy for both dose levels within a similar rangeError: Reference source not found, it is proposed to administer a ½ (0.25 ml) dose volume of RTS,S/AS02A (i.e. pediatric dose [0.25 ml dose volume]) in the planned phase I study in Mozambican children (Malaria-025) and this phase IIb study (Malaria-026).

A phase I study to assess the safety and immunogenicity of the vaccine in Mozambican children aged 1 through 4 years is planned. It is not anticipated that children in Mozambique will respond differently to the vaccine compared to West African children but this study will provide that reassurance. This study will use the 0, 1, 2 month schedule, as a step to integrate the vaccine into the Expanded Programme on Immunization regime. Blinded safety data after the first two doses will be available before proceeding to the phase IIb study.

Since the successful implementation of any malaria vaccine will depend on the existing immunization infrastructure, the target for this candidate vaccine is to evaluate its safety and efficacy within the Expanded Programme on Immunization (EPI) compatible schedule of 0,1,2 months.

Please refer to the Investigator Brochure for a review of the pre-clinical and clinical studies of the RTS,S/AS02A malaria vaccine.

### The AS02A adjuvant

The vaccine antigen, RTS,S is formulated in the GSK proprietary Adjuvant System 2 (AS02A): an oil in water emulsion containing the immunostimulants QS21 (a triterpene glycoside purified from the bark of *Quillaja Saponaria*) and 3D-MPL (3-deacylated monophosphoryl lipid A). The choice of AS02A as the formulation for a malaria pre-erythrocytic vaccine was initially based on extensive pre-clinical experimentation conducted by GSK Biologicals and collaborators. Pre-clinical immunogenicity studies in mice and in rhesus monkeys demonstrated conclusively that this formulation is a powerful inducer of both humoral and cell mediated immune (CMI) responses, including cytotoxic T lymphocytes (CTLs) and γ interferon (IFN-γ) secreting T-helper type 1 (Th1) lymphocytes.

A confirmation of the critical importance of adequate formulation and of the value of AS02A as an adjuvant system for pre-erythrocytic malaria vaccine was obtained in a clinical trial conducted in collaboration with the Walter Reed Army Institute of Research (WRAIR; WRMAL-003)Error: Reference source not found. In this Phase I/IIa trial, a vaccine consisting of the RTS,S antigen formulated in AS02A protected six out of seven volunteers (86% vaccine efficacy; relative risk = 0.14; 95% CI = 0.02, 0.88; p < 0.005) from infection following a homologous sporozoite challenge delivered by the bites of laboratory-reared infectious Anopheles mosquitoes. Two other vaccine formulations (i.e., AS04 and AS03) of the very same RTS,S antigen were also evaluated in this trial but afforded only marginal protection against challenge.

The first administration in younger age groups of the vaccine reconstituted with AS02A as novel adjuvant was in the Phase I study in 6 through 11 year-olds in The Gambia (Malaria-015) which was followed by a Phase I study in children aged 1 to 5 years, as described above. A full evaluation of safety and immunogenicity results obtained in both studies will be done prior to the start of this study.

In addition to RTS,S/AS02A, various vaccines consisting of different recombinant antigens formulated in AS02A adjuvant have already been evaluated in human subjects[[11]](#endnote-12).

The AS02A formulation to be used in malaria vaccine trials no longer contains any thimerosal as preservative and is supplied as monodose pre-filled syringes.

### Centro de Investigação em Saude de Manhiça

Centro deInvestigação em Saude de Manhiça (CISM) is the first peripheral health research centre in Mozambique, founded five years ago to undertake medical research into the priority health problems of the Mozambican population and to develop a strong capacity building component through training of Mozambican scientists. It was developed under a collaborative programme between the Ministry of Health in Mozambique, the Maputo School of Medicine (Universidade Eduardo Mondlane) and the Hospital Clinic in Barcelona (University of Barcelona), with core funding from the Spanish Agency for International Cooperation (AECI). At the centre of the study area is Manhiça District Hospital (Health Centre of Manhiça). There is close cooperation between CISM and the government services at the district hospital to provide an integrated system of morbidity surveillance.

The continuous demographic surveillance system at CISM forms part of the INDEPTH project (International Network for the continuous Demographic Evaluation of Populations and their Health) to monitor demographic indices across Africa[[12]](#endnote-13). Surveillance began in1996 in an area of approximately 10 km around the centre and covering a population of 36 000. All homes have been geo-referenced and each individual identified and given a unique number. All children have been issued with an identification card bearing this number. As part of the INDEPTH methodology, updates of the census are conducted every six months to identify in and out migrations, marriages, births and deaths. Verbal autopsies to ascertain cause of death are performed for all deaths occurring in children under 15 years of age.

### Manhiça District

CISM is located in the District of Manhiça (Maputo Province) in Southern Mozambique approximately 2 hours drive from the capital Maputo. This area consists of two distinct zones: the fertile lowlands which comprise the flood plains of the Incomati River and are sparsely inhabited and subject to intensive farming; and an escarpment of moderate height which gives rise to a flat plateau, which covers virtually the entire catchment area for Centro deInvestigação em Saude de Manhiça (CISM). There are two distinct seasons. The warm season is between November and April, during which most of the rainfall occurs (annual rainfall for 1998 was 1 100 mm and average temperatures of 24°C to 26°C) and a cool and dry season during the rest of the year (average temperatures of 18°C to 23°C).

The flood plains of the Incomati River provide ideal breeding conditions for mosquitoes throughout the year. The population living on the flat plateau suffer from the proximity of this area to the mosquito breeding grounds below in the flood plains. Entomological studies have been carried out and the vectors are known with the most common being *Anopheles funestus***.** The Entomological Inoculation Rate (EIR) is 15.1 infective bites/person/year across the study area.

With these environmental conditions, malaria in this region is transmitted all year round but with a slightly lower transmission intensity during the months from June to September, and is almost exclusively due to *P*. *falciparum*.

The population in Manhiça District, including the towns of Manhiça, the principle town, and Ilha Josina, lives in a peri-urban and rural setting. People of the area are mainly Xironga and Xichangana and their languages are often termed as Ronga and Changana. The two dominant religions are Islam and Christianity. Subsistence and crop farming and related industries are the main activities in the area.

Illiteracy is high: 24% of men and 47% of women. While 66% of men and 49% of women have had primary education, only 9% of men and 4% of women have had secondary education and less than 1% of both men and women have gone beyond their secondary education.

Villages in this area typically comprise a loose conglomeration of compounds separated by garden plots and grazing land. Houses are simple, with walls typically made of cane with thatched or corrugated roofs. In towns, houses are often grouped into family compounds and surrounded by grass fences. Towns grew substantially during the civil war in the 1980s as displaced people looked for refuge. Following the end of the civil war in 1992, few inhabitants returned to their original homes and displaced settlements have now been integrated into towns. Water source is mainly from community wells though some households have their own wells. In some areas there are community-sustained pumps. Both wells and pumps are supervised and chlorinated regularly by the District Water and Sanitation Department.

### Morbidity in the study area

The population structure is similar to that of many developing countries with 18% of the population under 5 years of age. Mortality in childhood is high; the total mortality rate in children under 5 years of age and infant mortality rates are 173 and 89 per 1000 live births respectively. Malaria is the largest single cause accounting for about a quarter of all deaths. Also important are the other common infectious diseases including acute lower respiratory tract infections (ALRI) and gastroenteritis. Malnutrition is prevalent and is given as the immediate cause of death in about a fifth of all deaths in this age group, although it may be a contributing factor in many more.

Recent data from the Manhiça area have shown that the human immunodeficiency virus 1 (HIV-1) rate in women of child-bearing age is approximately 15% (personal communication from Pedro Alonso). The rate of maternal to child transmission in Mozambique is unknown but is likely to be similar to other African countries[[13]](#endnote-14). Assuming a rate of 25%, an estimate of about 4% of the children in the study area will be positive for human immunodeficiency virus (HIV).

*Plasmodium falciparum* accounts for 90% of all malaria infections. The prevalence of asexual *P. falciparum* ranges from 13.7%‑21.7% at the end of dry season to 30.5%‑34.0% at the end of rainy season. *P. malariae* is present in all surveys with prevalence ranging between 1.4% and 2.9% while *P. ovale* shows a rather erratic prevalence pattern from survey to survey, ranging from 0% to 6.9%.

The malaria episode rate of children presenting to the hospital (defined as history or documentation of fever associated with any level of parasitaemia) is high. It ranges from 0.13 person years at risk for children aged 1 to 2 years to 0.08 person years at risk for children aged 4 to 5 years.

The prevalence of splenomegaly is 23% in children aged 1-5 years and the prevalence of haematocrit of 25% or less is 2.1% in children aged 1-5 years.

Bednet usage: 10% of households are estimated to use bednets.

### Manhiça District Hospital

The Manhiça District Hospital (Health Centre of Manhiça) is located in the centre of the study area. This 80-bed health facility includes a busy outpatient clinic, maternity and child care unit which includes EPI and nutritional services and a 24-hour Emergency Service. It is the closest referral hospital for all the study children.

Patient care at the hospital is provided by a staff of six medical doctors (four of which also work at CISM), three medical assistants (Tecnicos de medicina), 15 medical agents (Agentes de medicina), 21 nurses, 11 midwifes, one anaesthesiology assistant, one laboratory assistant, one psychiatry assistant and one surgery assistant. Outside of regular working hours, the emergency room is supervised by a medical assistant or doctor and there is always a medical doctor on call to manage emergencies.

A hospital-based surveillance system was set up at the hospital in 1996, in order to describe the epidemiology and clinical presentation of the important diseases in children living in this rural area of southern Mozambique. Records of all attendances of children up to sixteen years of age are kept, including the diagnosis, laboratory tests and treatments given. Integrating this system into the government hospital service has meant that the CISM has become centrally involved in the day to day running of the hospital, including the support of personnel, diagnostic procedures, equipment and drugs. The strengthening of the hospital over the last three years has contributed to the increased confidence of the local population in the local health services and to a very marked and sustained increase in hospital use. In brief, outpatient paediatric attendances have increased from 14 800 in 1997 to 29 800 in 2001. Similarly, the number of paediatric admissions has increased from 971 to 4577 over the same time period.

CISM has also carried out an analysis of the spatial distribution of hospital users in the study area. The increase in the use of the hospital as well as the analysis of CISM data suggest that the hospital is very widely used for important health conditions by the surrounding population and should any severe adverse events (SAEs) occur, the population will report to the hospital. Thus, the hospital surveillance system is believed to be a safe and comprehensive method to detect, treat and document any SAEs.

### Ilha Josina Health Facility

Ilha Josina Health Facility provides primary health care to the population in the north of the study area. It is currently staffed by nurses/midwives, who run EPI services, deliver maternity care and provide outpatient treatment services. There are no paediatric inpatient beds. Children needing admission are transferred to Manhiça District Hospital, a distance of 40 km along good tarmaced road.

During the study the service at the facility will be upgraded to include staffing by project clinicians (doctors or medical assistants) and dedicated vehicles for patient transfer to Manhiça Hospital (further details are given in section 4.1).

### Malaria drug treatment

Chloroquine remains the first line treatment for uncomplicated malaria, although recent studies in Manhiça suggest that the rate of adequate clinical response is only about 49%, with early treatment failure rates of 24% and late treatment failure rates of 27% (personal communication, Pedro Alonso). The Ministry of Health is currently evaluating the choice of alternative first line treatments, and it is anticipated that a decision will be taken before the end of 2002. Sulfadoxine-pyrimethamine (SP) has an adequate clinical response of 83% and amodiaquine of 93% (personal communication, Pedro Alonso). The final decision has not been reached yet but informal discussions with the Ministry of Health suggest that first line treatment will be changed to a combination of amodiaquine and SP. For this study specifically, the Principal Investigator has obtained permission from the Ministry of Health to use a combination of amodiaquine and SP as the first line treatment of malaria.

One study from Uganda reported clinical failure rates of 3% at 14 days after amodiaquine and SP treatment and parasitological failures in 10%. Preliminary results from an ongoing study in Mozambique have shown that of the 60 children between 6 and 59 months of age and treated with amodiaquine-SP for clinical disease, there were no clinical treatment failures by day 21 (parasitological failure rates have not yet been determined) personal communication, Pedro Alonso).

## Rationale

### Feasibility of the vaccine

Clinical evaluation of RTS,S/AS02A was started in 1995 under a US IND and several Phase I/II clinical trials in non-immune (malaria-naïve) and semi-immune (malaria-experienced) adults have since shown the RTS,S/AS02A vaccine to be safe with an acceptable reactogenicity profile in these study populations. Proof of concept of vaccine efficacy, first demonstrated against homologous sporozoite challenge, has been confirmed in an endemic area of seasonal malaria transmission. Although protective efficacy of this vaccine against infection appears partial and of short duration in the adult populations in which it has been evaluated thus far, these results are highly encouraging and suggest that the vaccine may be of benefit in protecting the main target population (infants and children residing in malaria endemic regions) from clinical disease.

The basic rationale for the evaluation of the RTS,S/AS02A candidate vaccine in the paediatric population takes into account that even a partially effective pre-erythrocytic vaccine may lead to a sustained reduction in the frequency and possibly the severity of clinical episodes. The vaccine would accomplish this outcome through its impact on initial sporozoite and liver stage parasite burden, leading to a significant reduction of the infectious inoculum of liver stage merozoites released into the blood stream.

This hypothesis is supported by the following indirect evidence:

1. Comparison of the mean pre-patent periods for those subjects developing malaria after homologous laboratory challenge shows that there is a highly significant statistical difference in the pre-patent periods for those subjects immunised with RTS,S/AS02A relative to concurrent controls[[14]](#endnote-15). Similarly in the study of adults in The Gambia, a delay to the first infection with falciparum malaria was shown^7^. These results are best explained by a reduction in the infectious inoculum of sporozoites, impairment of hepatic schizogony, or both, resulting in the release of significantly fewer merozoites from the liver. The delay in patency suggests that, in the setting of pre-existing immunity to asexual stage parasites, RTS,S/AS02A, apart from conferring protection against infection, may improve the clinical outcome of *P. falciparum* infection by decreasing the initial load of infected erythrocytes and allowing the immune system to respond more effectively to infection with asexual stage parasites^10^.
2. The results of several studies on the protection afforded by insecticide impregnated bed nets show that this intervention - whose mode of action is in certain ways similar to that of a partially effective pre-erythrocytic vaccine - is more effective in preventing severe malarial disease and death than infection^11^.

A vaccine that induces partial immunity against pre-erythrocytic stages of disease may provide protection to vulnerable young children from the severe forms of the disease, whilst continuing exposure allows them to build up natural immunity. Acquisition of natural immunity is believed to be critical in preventing a shift of severe disease to older age groups upon waning of vaccine-induced immunity.

#### Concurrent protection against Hepatitis B

Hepatitis B virus (HBV) infection remains an important public health problem across the developing world. It is widely recognised that vaccination is the only method of conferring long term protection against clinical disease^12^. In Sub-Saharan Africa, HBV infection is endemic with 15–20% of ***adults being hepatitis B antibody positive indicating a minimum level of previous exposure[[15]](#endnote-16). A proportion of these adults will become chronic carriers of HBV*** with an increased risk of developing cirrhosis or primary hepatocellular carcinoma of the liver. The Hepatitis B surface antigen (HBsAg) contained in the RTS,S/AS02A vaccine is encoded by the hepatitis B virus S protein gene that is identical to the gene used to express HBsAg in GSK Biologicals’ Engerix-B™ vaccine against Hepatitis B, which, despite its efficacy, has not yet been implemented universally in developing countries since its registration in 1986[[16]](#endnote-17). Results obtained with RTS,S/AS02A in adults show that seroprotective levels of anti-HBsAg titres (defined as ≥ 10 mIU/ml) are rapidly achieved^5^, indicating that the vaccine could offer concurrent protection against both diseases. A vaccine that offers dual protection would be of great benefit in the developing world and this study provides the opportunity to compare the immunogenicity of RTS,S/AS02A to that of Engerix-B™. **(Amended August 13, 2002)**

Up to 4 doses of RTS,S/AS02A have been safely administered to Hepatitis B seronegative and seropositive adults as well as chronic asymptomatic carriers of HBsAg. To date, at least 481 adult subjects (215 malaria-naïve adults and 266 malaria-experienced adults) have received 1254 full doses (471 and 782 doses respectively) of the RTS,S/AS02A malaria vaccine. In Malaria-004 (an open study with RTS,S/AS02A) and Malaria-005 (a double-blind study with RTS,S/AS02A and control rabies vaccine), both in malaria-endemic areas, 53 subjects were identified as being chronic asymptomatic carriers of HBsAg. Twenty-four of these chronic carriers were immunised with RTS,S/AS02A. In these subjects, the reactogenicity profile was comparable to that of healthy subjects (non-carriers).

### Rationale for the design of the phase IIb study

#### Rationale for efficacy endpoints

This trial proposes to examine the efficacy of the vaccine at two points in the pathogenesis of malaria disease: infection and clinical disease of moderate severity in two cohorts.

These two endpoints will be measured simultaneously in the two cohorts. The primary endpoint of protection against clinical disease will be assessed in Cohort 1 and the secondary endpoint of time to infection in Cohort 2.

The factors involved in determining the clinical manifestation of disease and the transition from exposure to infection to disease are not well understood. What is considered important for a useful malaria vaccine is that it prevents disease, particularly severe disease and death. For this reason the primary endpoint of the trial, on which the decision on the future development will be based, is clinical disease. However, it is also important to know whether the RTS,S/AS02A malaria vaccine prevents infection in the paediatric population in order to understand the mechanism of action and to determine the vaccine's future potential in single or multiple antigen vaccines. This is further explained in section 1.2.2.1.2 (Infection).

##### Clinical disease

The primary case definition for the trial was chosen with the following considerations in mind. It was important that it represents true cases of malaria at the more severe end of the disease spectrum. Therefore, cases contributing to the primary case definition are children who have been judged to be ill by their parents and brought for treatment to their local hospital or health facility. These cases are defined as febrile (≥ 37.5°C) with a parasitaemia level above a cut off value. In malaria endemic areas parasitaemia is common in the absence of symptoms. This means that sick children with a range of conditions may have coincidental parasitaemia, but that this is not the cause of the illness. Methodology proposed by T. Smith models the relationship between parasite density and fever in a population and defines a threshold above which the parasitaemia is likely to be the cause of the fever. In this population this was determined as 2500 parasites per µl and using this definition, the case definition has been estimated to be 91% specific and 95% sensitive[[17]](#endnote-18). The high specificity will ensure that the estimate of effect is not a significant underestimate of the true vaccine efficacy.

The primary endpoint is time to first clinical attack, which is a suitable method for assessing the efficacy of a vaccine inducing partial immunity to malaria. This type of vaccine, which gives partial protection to most vaccinees (rather than a vaccine that protects completely a proportion of the population) cannot be assessed using a comparison of the proportions of people affected because over time, as progressively more subjects experience their first attack, the measured efficacy will trend to zero.

Other measures of vaccine efficacy have been included as secondary endpoints. The range of clinical case definitions for malaria encompass a range of disease severity. The endpoint of history or documented fever plus any level of parasitemia may be relatively non-specific, but represents the large number of children, who attend health centres and require anti-malarial treatment, and is therefore of interest to public health. The definition incorporating high levels of parasitemia (15 000 per µl) represents disease at the more severe end of the spectrum and is highly specific (95%), but less sensitive (87%). Malaria parasite density may be a good assessor of a partially effective vaccine, which may act by controlling the level of parasitemia.

Children’s haematocrits fall in association with clinical attacks of malaria and the haematocrit is therefore a measure that reflects the cumulative frequency and severity of malaria episodes. At the cross-sectional surveys at months 8½ and 21, children will all be assessed with a blood slide for malaria parasite density and a haematocrit. Haematocrit below 25% is moderate anaemia that warrants treatment and a value of below 15% is severe anaemia and is frequently life-threatening[[18]](#endnote-19). If the vaccine showed efficacy against moderate anaemia, it is likely that it would also show efficacy against severe malaria anaemia.

##### Infection

Infection is a necessary step on the pathway to development of clinical disease and it is therefore important to the understanding of the mechanism of action of the vaccine to determine the effect on infection in the context of the trial.

Stage-specific immunity is likely to be one of the main factors determining the clinical course of malaria. However, the understanding of malaria immunity is still incomplete. Two hypotheses have been formulated with regards to pre-erythrocytic immunity. The first one proposes that pre-erythrocytic immunity needs to be 100% efficacious (in other words, induce sterilizing immunity) in order to prevent the emergence of merozoites in peripheral blood and the subsequent risk of clinical disease developing in the infected individual. This has led to the thinking that unless a pre-erythrocytic vaccine induces sterilising immunity, it will have no efficacy in preventing disease or death. The second hypothesis implies that a reduction in the number of viable sporozoites infecting hepatocytes and/or a reduction in the number of emerging merozoites may fail to reduce the incidence of infection, but may lead to a delay in reaching the threshold in parasite densities associated with clinical manifestations. This delay in time, albeit short, may be sufficient to allow immune mechanisms to operate and reduce the risk of clinical malaria without modifying the prevalence of infection. This second hypothesis would imply that a pre-erythrocytic vaccine may only be partially effective at preventing new infections, but may be more efficacious at preventing disease.

RTS,S/AS02A is a pre-erythrocytic vaccine, which has shown efficacy in reducing the risk of new infections following homologous sporozoite challenge and in adults living in malaria endemic areas. Measuring efficacy against natural infection will aid in the interpretation of the results of the efficacy against disease. If RTS,S/AS02A does not reduce the risk of clinical malaria, it may be that this is a consequence of having failed to reduce the incidence of new infections. Alternatively, it may be that the vaccine's pre-erythrocytic component has been moderately efficacious, but failed to impact on subsequent blood-stage infection, and that it has therefore not reduced the incidence of clinical malaria (hypothesis 1).

If the vaccine reduces the risk of clinical malaria, this may be a consequence of the reduction in the incidence of new infections. Alternatively, the vaccine may partially or totally fail to prevent new infections, but remain moderately (or highly) efficacious in preventing disease (hypothesis 2).

This trial offers a unique opportunity to contribute to our understanding of the factors involved in the transition from infection to disease, and to contribute data to support one of the two current hypotheses with regards to the importance of pre-erythrocytic immunity in determining the risk of malaria disease. Currently, development of a pre-erythrocytic vaccine candidate may be discontinued if it fails to reduce the incidence of new infections based on hypothesis 1. However, should hypothesis 2 be correct, and only hypothesis 1 had been tested, a number of potentially useful vaccines, especially for populations in endemic countries, may be discarded. Similarly a vaccine, which reduces the burden of infection but fails to prevent disease, although not valuable as a single antigen vaccine, may prove useful in a multiple antigen vaccine. This information will be useful to guide future vaccine development decisions.

#### Rationale for parasite genotyping

RTS protein is derived from a sporozoite surface antigen of the *P. falciparum* strain NF54. In the efficacy study conducted in Gambian men under conditions of natural challenge, protection was not limited to the NF54 parasite genotypeError: Reference source not found. In this study, ***samples for the determination of parasite strain will be collected from Cohorts 1 and 2 for all efficacy endpoints to allow for the possibility of evaluating strain-specific efficacy*** ~~protection against infection due to multiple strains will be assessed in the children in Cohort 2~~. Parasites  ~~from the first parasite-positive sample for each child~~ will be characterised at the Th2R and Th3R epitope sequences of the gene encoding the circumsporozoite protein. **(Amended August 13, 2002) (Amended January 17, 2003)**

#### Rationale for sample size for the primary endpoint

Although trials are often powered to detect differences between vaccinated and unvaccinated, the decision to proceed with the clinical development plan is conditional on the vaccine demonstrating efficacy of 50% against clinical episodes in this phase IIb study in Mozambique. Therefore the sample size has been calculated to ensure that there is precision around the estimate of effect and that if the true vaccine efficacy is 50% that there is 80% power to detect a lower limit of vaccine efficacy of 15%.

#### Rationale for choice of control vaccines

A vaccine that is of benefit to the control group is preferable to the use of placebo injections and will be more acceptable to all the ethical review bodies for this study (the local Mozambican ethical committee, the co-sponsor’s (MVI) investigational review board [IRB] and the IRB of the University of Barcelona, where the Principal Investigator is affiliated) and the study population.

***Children between the ages of 12 to24 ~~18~~ months*** Hepatitis B vaccination (at 2, 3 and 4 months of age in combination with diphtheria-tetanus-pertussis and polio vaccines) was introduced into the EPI schedule of Mozambique in July 2001. It is estimated that 83% of children have received all immunizations by 12 months of age (personal communication from Pedro Alonso). It is probable that most children under the age of ***24*** ~~18~~ months, at the time the trial is projected to start, will already have received immunization against hepatitis B. Re-immunization with a hepatitis B vaccine would not provide an individual benefit to children under the age of ***24*** ~~18~~ months in this study population. It is therefore proposed to use an alternative control regime in these children: a 7‑valent pneumococcal conjugate vaccine (Prevnar®) will be administered at doses 1 and 3 and a conjugate *Haemophilus influenzae* type b vaccine (Hiberix™) at for the second dose. These vaccines will be administered according to the licensed vaccination schedules for this age group and will thus be of benefit to these children[[19]](#endnote-20),[[20]](#endnote-21).
**(Amended January 17, 2003)**

Acute lower respiratory tract infections (ALRI) are a major cause of mortality and morbidity in children in the developing world, causing about 3 million deaths in children annually[[21]](#endnote-22). Investigation to determine the causative organism is not routine in most of Africa however, many research studies have now been conducted in African centers and have produced consistent results. The most important causes are: *Streptococcus pneumoniae* (the pneumococcus), *Haemophilus influenzae* and the respiratory syncitial virus (RSV)[[22]](#endnote-23). The pneumococcus is the largest single bacterial cause of ALRI in the developing world. Of the more than 90 serotypes of pneumococcus, the 7 serotypes contained within Prevnar® (4, 6B, 9V, 14, 18C, 19F and 23F) are estimated to cover about 67% of invasive infections in Africa[[23]](#endnote-24). Although Prevnar® (Wyeth Ledrele Vaccines) is licensed in several parts of the world, it is only the US that has incorporated pneumococcal conjugate vaccines into their national immunization schedule. Evidence to support a universal recommendation for pneumococcal immunisation by WHO is being sought. Pneumococcal vaccination is expected to substantially reduce the amount of ALRI experienced and vaccine trials are being conducted in South Africa, The Gambia and The Phillipines to confirm this.

WHO recommends Hib immunisation for children. Key evidence supporting this decision was a vaccine trial examining the effect of Hib vaccination conducted in The Gambia. As expected the vaccine was highly protective against invasive Hib disease (95% CI 67-100), but in addition had a vaccine efficacy of 21.1% (95% CI of 4.6-34.9) against all cases of radiologically defined pneumonia.

Acute bacterial meningitis is another important cause of mortality; in total between 100 000 and 500 000 deaths annually^17^. Survivors frequently are left with a severe permanent disability[[24]](#endnote-25). Of the three major causes of acute bacterial meningitis, *Streptococcus pneumonia*, *Haemophilus influenzae* and *Neisseria menigitidis*, it is the pneumococcus and *Haemophilus influenzae* that account for the cases outside the epidemic peaks 17, [[25]](#endnote-26). Vaccination against Hib is of well-established benefit for the prevention of meningitis[[26]](#endnote-27).

***Children over 24 ~~18~~ months of age***
In children over the age of ***24*** ~~18~~ months, hepatitis B vaccine is proposed as the control vaccine. In Sub-Saharan Africa, HBV infection is endemic leading to, in a proportion of infected subjects, chronic carriage and the complications of chronic liver disease and hepatoma. Thus, the control group would benefit from this vaccine.
**(Amended January 17, 2003)**

#### Rationale for conducting hepatitis B serology in Cohort 2

This study will compare the immunogenicity of a licensed hepatitis B vaccine (Engerix-B™) and RTS,S/AS02A. Immunogenicity data obtained thus far have consistently shown excellent anti-HBs antibody geometric mean titres and derived seroprotection rates, however, no comparisons with commercial hepatitis B vaccines have been performed to date.

A comparison of RTS,S/AS02A with an established hepatitis B vaccine, with respect to anti-HBs antibodies, will be important at this stage in the clinical development before proceeding to infant studies. To avoid giving infants three doses of RTS,S/AS02A and three doses of hepatitis B vaccine in the first year of life in the planned infant studies, it is proposed to assess the feasibility to replace the existing hepatitis B antigen in the EPI vaccination schedule with RTS,S/AS02A.

The quality of the immune response to vaccination with HBsAg (whether in Engerix-B™ or RTS,S/AS02A) cannot be evaluated directly by a neutralisation assay since no such assay is available due to the lack of *in vitro* replication of the hepatitis B virus. Nevertheless, the generation of monoclonal antibodies against various epitopes of HBsAg have allowed the identification of a protective epitope (RF1). The RF1 monoclonal antibody (RF1 mAb) recognises this conformational epitope on peptide 124-137 of the HBsAg. The RF1 antibody has been shown to protect chimpanzees against infection with the virus[[27]](#endnote-28). In the absence of a neutralisation assay for HBV, RF1-like antibodies in serum from vaccinees can be used as a surrogate marker of neutralising capacity and thus provide a qualitative evaluation of the immune response to vaccination. Therefore, RF1-like antibodies will be measured in all subjects in Cohort 2 at screening and then 30 days post third dose.

The rationale for performing serology testing for anti-HBs antibodies in Cohort 2 rather than Cohort 1 is based on the mistrust of venous blood sampling by the community. Whereas the community are accepting of capillary blood sampling by finger prick, they do not readily accept venous sampling of healthy children. Adding venipuncture to a study results in a higher rate of non-compliance. It has been estimated that without venipuncture the dropout rate is 10%, but it rises to 20% for studies including venipuncture. So that venous blood sampling does not jeopardize compliance in children in Cohort 1, children for Cohort 2 will be recruited from Ilha Josina, a site adjacent to but not overlapping the catchment area for Cohort 1.

The additional blood volumes needed for the hepatitis B serology will require venipuncture at some time points. Therefore, to minimise the total number of children enrolled in the this phase IIb study, this endpoint is proposed for a separate cohort (Cohort 2) and a non-compliance rate of 20% has been applied to the sample size for this cohort only.

#### Assessment of vaccine reactogenicity

Adverse event data will be collected on ***990*** ~~960~~ recipients of the paediatric dose (0.25 ml dose level) of RTS,S/AS02A in the course of this phase IIb trial in 1-4 year old children. This will allow for the reactogenicity profile of the vaccine to be defined with higher precision than previously done in the smaller paediatric phase I studies (Malaria-015 and Malaria-020).
**(Amended January 17, 2003)**

The proposed schedule in this trial is 0,1,2 months as opposed to 0,1,3 months in the phase I studies in the Gambia (Malaria-015 and 020). Based on results of a previous study in malaria-naïve adults, which evaluated vaccination at 0,1,3 months versus 0,7,28 days and showed that schedule had no effect on the reactogenicity profile, no significant impact on reactogenicity is expected after vaccination at 0,1,2 monthsError: Reference source not found. In the paediatric phase I study in 1-5 year-olds, a slight trend with increasing dose level and number of doses was only seen for the local symptom of swelling and the general symptom of irritability/fussiness (please see section 1.1.1 for a summary of the data). Although the study children are not expected to react any differently to the Gambian children, a blinded review of safety and reactogenicity data after two doses from the phase I trial (using a 0,1,2 month vaccination schedule) in 1-4 year-old children in Manhiça (Mozambique) will be performed before proceeding with this phase IIb study.

The control vaccines are all licensed vaccines with established reactogenicity profiles, thus the adverse event rate in the recipients of these vaccines is expected to be similar to previous experience with these vaccines. However, in this developing community in Mozambique, the acute febrile illnesses of childhood, particularly respiratory tract infections and malaria, occur frequently. Many of these common viral and bacterial infections will be seen with equal frequency among the recipients of hepatitis B vaccine and RTS,S/AS02A and the comparison with the control group is useful to distinguish vaccine effects from those due to intercurrent illness. However, those younger children, who receive immunization against pneumococcal and haemophilus disease, could be expected to be protected against some of the most common infections of childhood. With the small numbers enrolled in this group, it is unlikely to reach levels of statistical significance.

# Objectives

## Primary objective

The primary objective of the study is to assess the efficacy of RTS,S/AS02A candidate malaria vaccine against clinical episodes of *P. falciparum* malaria in children aged 1 to 4 years at first vaccination, over a 6 month surveillance period starting 14 days after dose 3 (day 74), in Cohort 1.

See Section 10.2 for delineation of primary endpoint and Section 10.1 for delineation of clinical case definitions.

## Secondary objectives

The secondary objectives correspond exclusively to data collected and endpoints during the double-blind phase of the study (months 0 to 8½). See Section 10.1 for delineation of clinical case definitions.

### Cohort 1

***Efficacy***

To assess, in children (1 to 4 years at first vaccination) the efficacy of RTS,S/AS02A candidate malaria vaccine against:

- clinical episodes of *P. falciparum* malaria of differing severity over a 6 month surveillance period starting 14 days after dose 3 (day 74).
- anaemia presenting clinically over a 6 month surveillance period starting 14 days after dose 3 (day 74) or detected at cross-sectional survey at 6½ months after dose 3 (month 8½).
- asexual falciparum parasitaemia (prevalence and density) at 6½ months after dose 3 (month 8½).

***Safety***

To assess in children (1 to 4 years of age at first vaccination):

- safety of RTS,S/AS02A malaria vaccine until 6½ months after dose 3 (month 8½).
- reactogenicity of RTS,S/AS02A malaria vaccine from the time of first vaccination until one month after dose 3 (day 90).

***Immunogenicity***

- To assess the humoral immune response to the circumsporozoite protein (CS) antigen of the RTS,S/AS02A malaria candidate vaccine in children (1 to 4 years of age at first vaccination).

See Section 10.3.1 for delineation of secondary endpoints corresponding to Cohort 1.

### Cohort 2

***Efficacy***

- To assess in children (1 to 4 years of age at first vaccination) the efficacy of RTS, S/AS02A malaria candidate vaccine in delaying time to first asexual falciparum infection.

***Safety***

To assess in children (1 to 4 years of age at first vaccination):

- safety of RTS,S/AS02A malaria vaccine until 6½ months after dose 3 (month 8½).
- reactogenicity of RTS,S/AS02A malaria vaccine from the time of first vaccination until one month after dose 3 (day 90).

***Immunogenicity***

To assess in children (1 to 4 years of age at first vaccination), the humoral immune response to:

- the HBs antigen of the RTS,S/AS02A malaria candidate vaccine.
- the CS antigen of the RTS,S/AS02A malaria candidate vaccine.

See Section 10.3.2 for delineation of secondary endpoints corresponding to Cohort 2.

## Tertiary objectives

All the tertiary objectives correspond to endpoints and data collected during the single-blind phase of the study (months 8½ to 21). See Section 10.1 for delineation of clinical case definitions.

### Cohort 1

***Efficacy***

To assess in children (1 to 4 years of age at first vaccination) the efficacy of RTS,S/AS02A candidate malaria vaccine against:

- clinical episodes of *P. falciparum* malaria of differing severity over the surveillance period.
- anaemia presenting clinically over the surveillance period or detected at cross-sectional survey at 19 months after dose 3 (month 21).
- asexual *P. falciparum* parasitaemia (prevalence and density) at 19 months post dose 3 (month 21).

***Safety***

- To assess in children (1 to 4 years of age at first vaccination) the safety of the RTS,S/AS02A malaria candidate vaccine during months 8½ to 21.

***Immunogenicity***

- To assess the humoral immune response to the CS antigen of the RTS,S/AS02A malaria candidate vaccine at 19 months post dose 3 (month 21) in children (1 to 4 years of age at first vaccination).

See Section 10.4.1 for delineation of tertiary endpoints for Cohort 1.

### Cohort 2

***Safety***

- To assess in children (1 to 4 years of age at first vaccination) the safety of the RTS,S/AS02A malaria candidate vaccine during months 8½ to 21.

***Immunogenicity***

To assess in children (1 to 4 years of age at first vaccination) the humoral immune response to:

- the CS antigen of the RTS,S/AS02A malaria candidate vaccine at 19 months post dose 3 (month 21).
- the HBs antigen of the RTS,S/AS02A malaria candidate vaccine at 19 months post dose 3 (month 21).

See Section 10.4.2 for delineation of tertiary endpoints for Cohort 2.

# Study Design Overview

**(Amended January 17, 2003)**

- Phase IIb study in one centre and two study sites (towns of Manhiça and Ilha Josina).
- The necessary number of healthy male and female children aged 1 to 4 years (inclusive) will be screened in order to enrol ***1980*** ~~1920~~ subjects in the study.
  **(Amended January 17, 2003)**
- Duration per subject: approximately 24 months.
- Screening will be performed within ***90*** ~~35~~ days.
  **(Amended January 17, 2003)**
- Enrolment will be performed within 12 weeks.
- Controlled:
- 3 doses of hepatitis B vaccine (Engerix‑B™) to children of ***24*** ~~18~~ months and older.
- 2 doses of a 7-valent pneumococcal conjugate vaccine (Prevnar®, Wyeth Lederle Vaccines) at first and third vaccination and 1 dose of Haemophilus influenzae type b vaccine (Hiberix™) at second vaccination to children less than ***24*** ~~18~~ months. **(Amended January 17, 2003)**
- 3 doses of vaccine (RTS,S/AS02A or control) will be administered using a 0, 1 and 2 month vaccination schedule.
  A full dose (0.5 ml) of the control vaccine or a paediatric dose (0.25 ml dose volume) of the RTS,S/AS02A vaccine will be administered at each vaccination.
- Blinding:
- Vaccination and surveillance period (double-blind phase) from month 0 to the data lock point for primary and secondary objectives (month 8½).
- Single-blind surveillance period (study subjects will remain blinded to treatment allocation until study end as will study staff responsible for endpoint evaluation at health facilities and at cross-sectional surveys) from month 8½ to data lock point for analysis of tertiary objectives (month 21).
- Subjects will be randomly assigned in a 1:1 fashion to receive either the RTS,S/AS02A candidate malaria vaccine or one of the control vaccine regimes. See Section 6.4 for a detailed description of the randomization method.
- Two cohorts will evaluate the effect of the malaria candidate vaccine on clinical malaria disease and malaria infection (Cohorts 1 and 2 respectively, with 1564 subjects in Cohort 1 and ***416*** ~~356~~ subjects in Cohort 2). In Cohort 2 where the efficacy of the vaccine against infection will be evaluated, subjects will receive 4 weeks prior to onset of surveillance one dose of sulfadoxine-pyrimethamine and amodiaquine per day for 3 days, so as to clear any parasitaemia. **(Amended January 17, 2003)**
- Self-contained study, i.e. this study will be independent of other studies.
- Data collection will be by electronic remote data entry (RDE)

# Study Site and Population

## Study site

The study will be conducted in the Manhiça District of Mozambique. Refer to Sections 1.1.3 to 1.1.7 of introduction for a detailed description of study area and the participating health facilities.

CISM is located adjacent to the District Hospital, in Manhiça the principal town of Manhiça District. CISM is connected by a tarmac road to the Ilha Josina health facility, which lies 40 km to the north of Manhiça town. Communication between the coordination centre at CISM and Ilha Josina is by means of short wave radio. Project staff including providers of clinical care, fieldworkers, laboratory staff and drivers will be resident at the two sites.

Children for Cohorts 1 and 2 will be recruited from Manhiça District, in the areas around Manhiça town and from Ilha Josina respectively. Vaccinations will be performed at both Manhiça Hospital and Ilha Josina Health Facility.

A full morbidity surveillance system for the detection of clinical cases and serious adverse events is established at Manhiça Hospital and will be established by study start at Ilha Josina Health Facility. At both sites, this will provide 24-hour provision of clinical care supervised by a clinician (doctor/medical assistant) with facilities and staff to perform blood slide examination for malaria parasites. Although at Ilha Josina Health Facility there are emergency drugs including oxygen available, there are no inpatient facilities. Children requiring inpatient care will be transferred to Manhiça Hospital by project vehicles that will be permanently stationed at Ilha Josina, to ensure 24-hour availability.

## Number of subjects

The necessary number of subjects will be screened in order to enrol ***1980*** ~~1920~~ eligible subjects; 1564 to Cohort 1 and ***416*** ~~356~~ to Cohort 2. Subjects will be recruited by non-coercive methods according to existing policies of the CISM (see Appendix C). Subjects will be male and female children of 1 to 4 years of age (up to but not including their 5^th^ birthday) residing in the Manhiça District. See Section 6.4 for a detailed description of the criteria used in the estimation of sample size.
**(Amended January 17, 2003)**

Only subjects for whom the investigator believes the requirements of the protocol will be complied with (e.g. completion of the diary cards, return for follow-up visits) should be enrolled in the study.

## Inclusion criteria

- Healthy male and female children, 1 to 4 years of age at the time of first vaccination (up to but not including 5^th^ birthday).
- Written/thumbprinted and witnessed informed consent obtained from the parents or legal guardians, after they have been informed of the risks and benefits of the study in a language, which they clearly understand.
- Free of obvious health problems as established by medical history and clinical examination before entering into the study.
- Availability for the duration of the immunisation and follow-up period.

## Exclusion criteria for enrolment

The following criteria should be checked at the time of study entry. If any apply at the time of study entry, the subject must not be included in the study:

- Major congenital defects or serious chronic illness. Acute or chronic, clinically significant pulmonary, cardiovascular, hepatic or renal functional abnormality, as determined by physical examination or laboratory screening tests (see below). Chronic carriers of the HBsAg will not be excluded from the clinical trial provided they are clinically asymptomatic.
- Haematocrit of less than 25%.
- History of allergic reactions to vaccination.
- History of allergic reactions to any component of the Hiberix™, Prevnar® or Engerix-B™ vaccines.
- Chronic administration (defined as more than 14 days) of immuno-suppressants or other immune modifying drugs within six months prior to the first vaccine dose (for corticosteroids, this will mean prednisone, or equivalent, ≥ 0.5 mg/kg/day. Inhaled and topical steroids are allowed.)
- Previous vaccination with an experimental vaccine.
- Previous vaccination with hepatitis B vaccine (as documented on health card) in children equal to or more than ***24*** ~~18~~ months of age. **(Amended January 17, 2003)**
- Simultaneous participation in any other clinical trial.
- Any other findings which in the opinion of the investigators would increase the risk of having an adverse outcome from participation in the trial.
- Severe malnutrition defined as weight for ***height*** ≤ -3z scores[[28]](#endnote-29).
  **(Amended August 13, 2002)**
- History of surgical splenectomy.
- Use of any investigational or non-registered drug or vaccine other than the study vaccine(s) within 30 days preceding the first dose of study vaccine, or planned use during the study period.
- Planned administration of a vaccine not foreseen by the study protocol within 30 days before the first dose of vaccine. An exception, is the receipt of an EPI or licensed vaccine (measles, oral polio, meningococcal and combined diphtheria/pertussis/tetanus vaccines) which may be given 14 days or more before or after vaccination.
- Administration of immunoglobulins and/or any blood products within the three months preceding the first dose of study vaccine or planned administration during the study period.
- ~~Any confirmed or suspected immunosuppressive or immunodeficient condition, including human immunodeficiency virus (HIV) infection.~~ ***A diagnosis or clinical suspicion of an immunosuppressive or immunodeficient condition of any cause based on a full clinical history and medical examination.* (Amended August 13, 2002)**
- A family history of congenital or hereditary immunodeficiency.
- Same-sex twin.
- Lack of healthcard (i.e. no previous vaccination record).

## Elimination criteria during the study

The following criteria should be checked at each visit subsequent to the first visit. If any become applicable during the study, the subject will not be required to discontinue the study but may be eliminated from primarily the immunogenicity analysis. See Section 10.5 for definition of study cohorts/datasets to be evaluated.

- Use of any investigational or non-registered drug or vaccine other than the study vaccine(s) during the study period.
- Chronic administration (defined as more than 14 days) of immunosuppressants or other immune-modifying drugs during the study period (for corticosteroids, this will mean prednisone, or equivalent, ≥ 0.5 mg/kg/day. Inhaled and topical steroids are allowed.)
- Administration of a vaccine not foreseen by the study protocol during the period starting from 30 days before each dose of vaccine(s) and ending 30 days after. An exception, is the receipt of an EPI or licensed vaccine (measles, oral polio, meningococcal and combined diphtheria/pertussis/tetanus vaccines) which may be given 14 days or more before or after vaccination.
- Administration of immunoglobulins and/or any blood products during the study period.

## Contraindications to vaccination

The following adverse events constitute absolute contraindications to further administration of vaccine; if any of these adverse events occur during the study, the subject must not receive additional doses of vaccine but may continue other study procedures at the discretion of the investigator. The subject must be followed until resolution of the event, as with any adverse event (see Section 8.4):

- Anaphylactic reaction following the administration of vaccine(s).
- Any confirmed or suspected immunosuppressive or immunodeficient condition, including human immunodeficiency virus (HIV) infection.

The following adverse events constitute contraindications to administration of vaccine at that point in time; if any one of these adverse events occurs at the time scheduled for vaccination, the subject may be vaccinated at a later date, within the time window specified in the protocol (see Sections 5.3, 5.5 and 5.2.11), or withdrawn at the discretion of the investigator. The subject must be followed until resolution of the event, as with any adverse event (see Section 8.4).

- Acute disease at the time of vaccination. (Acute disease is defined as the presence of a moderate or severe illness with or without fever. All vaccines can be administered , at the investigator’s discretion, to persons with a minor illness such as diarrhea, mild upper respiratory infection with or without low-grade febrile illness, i.e., axillary temperature < 37.5°C
- Axillary temperature ≥ 37.5°C.

# Conduct of Study

## Ethics and regulatory considerations

The study will be conducted according to Good Clinical Practice, the Declaration of Helsinki (as found in Protocol Appendix A), and local rules and regulations of the country. The study protocol will be submitted for ethical review to the Joint Mozambican Government Ethics Review Committee, to the IRB at Barcelona University Hospital in Spain for the collaborating institution and to the IRB of Malaria Vaccine Initiative at Program for Appropriate Technology in Health (MVI at PATH): Human Subjects Protection Committee (HSPC) for the co-sponsor.

### Institutional Review Board/Independent Ethics Committee (IRB/IEC)

The IRB/IEC must be constituted according to the local laws/customs of each participating country. It is recommended that it should include:

1. At least five members.
2. At least one member whose primary area of interest is in a non-scientific area.
3. At least one member who is independent of the institution/ study site.

Only those IRB/IEC members who are independent of the investigator and the sponsor of the study should vote/ provide opinion {delete as appropriate} on a study-related matter.

A list of IRB/IEC members and their qualifications should be obtained by the investigator.

This protocol and any other documents that the IRB/IEC may need to fulfil its responsibilities, including subject recruitment procedures, and information about payments and compensation available to subjects, will be submitted to an appropriate Committee or Board by GlaxoSmithKline Biologicals’ Study Monitor i.e., Central Study Coordinator and their written unconditional approval should be in the possession of the investigator and the sponsor before commencement of the study. Relevant GlaxoSmithKline Biologicals’ data will be supplied by GlaxoSmithKline Biologicals’ Study Monitor, i.e., Central Study Coordinator to the investigator for submission to the independent IRB/IECs for the protocol's review and approval. Verification of IRB/IEC unconditional approval of the protocol and the written informed consent statement will be transmitted by the investigator to the Central Study Coordinator using the standard notification form, generally prior to shipment of vaccine supplies and CRFs to the site. This approval must refer to the study by exact protocol title and number, and should identify the documents reviewed and state the date of review.

No deviations from, or changes to, the protocol should be initiated without prior written IRB/IEC approval/ favorable opinion of an appropriate amendment, except when necessary to eliminate immediate hazards to the subjects or when the change(s) involves only logistical or administrative aspects of the study (e.g., change of monitor(s), telephone number(s)). Modifications are submitted to the IRB/IEC for information only. However, written verification that the modification was submitted should be obtained from each IRB/IEC. Approvals/ verifications must be transmitted in writing to the investigator by GlaxoSmithKline Biologicals’ Study Monitor, i.e. Clinical Study Coordinator.

The IRB/IEC must be informed by GlaxoSmithKline Biologicals’ Study Monitor, i.e., Central Study Coordinator of

- all subsequent protocol amendments, informed consent changes or revisions of other documents originally submitted for review
- serious and/or unexpected adverse events occurring during the study, where required
- all subsequent protocol modifications (for information)
- new information that may affect adversely the safety of the subjects or the conduct of the study
- an annual update and/or request for re-approval, where required
- when the study has been completed, where required.

### Informed consent

The principles of informed consent in the 1996 edition of the Declaration of Helsinki (Protocol Appendix A) should be implemented in each clinical study before any protocol-specified procedures or interventions are carried out.

Information should be given in both oral and written form whenever possible and deemed appropriate by the IRB/IEC.

The written consent document will: embody the elements of informed consent as described in the 1996 edition of the Declaration of Helsinki, outlined below, adhere to the ICH Harmonized Tripartite Guideline for Good Clinical Practice and also comply with local regulations.

An investigator or designate will describe the protocol to potential subjects’ parents/guardians face to face. The Subject Information and Consent Form may be read to the subjects’ parents/guardians, but, in any event, the investigator shall give the subjects’ parents/guardians ample opportunity to inquire about details of the study and ask any questions before dating and signing the Consent Form. All illiterate individuals will have the study, the Subject Information and Consent Form explained to them point by point by the interviewer in the presence of an impartial witness. The witness should personally sign/thumbprint and personally date the consent form.

Subjects’ parents/guardians must be informed that the trial involves research, purpose of the trial, those aspects of the trial that are experimental, expected benefits, possible risks (including a statement that the particular treatment or procedure may involve risks to the subject which are currently unforeseeable), the expected duration of the subject’s participation in the trial, the subject’s responsibilities, the approximate number of subjects involved in the trial, procedures of the research study including all invasive procedures and the probability for random assignment to treatment groups. Subjects’ parents/guardians will be informed that they will be notified in a timely manner if information becomes available that may be relevant to their willingness to continue participation in the trial. They must also be informed of alternative procedures that may be available and the important potential benefits and risks of these available alternative procedures. Subjects’ parents/guardians must receive an explanation as to whether any compensation and any medical treatments are available if injury occurs and, if so, what they consist of, or where further information may be obtained. Subjects’ parents/guardians must be informed of the anticipated financial expenses, if any, to the subject for participating in the trial as well as any anticipated prorated payments, if any, to the subject for participating in the trial. They must be informed whom to contact (e.g. the investigator) for answers to any questions relating to the research project. Information will also include the foreseeable circumstances and/or reasons under which the subject’s participation in the trial may be terminated. The subjects’ parents/guardians must be informed that the subject’s participation is voluntary and that they are free to withdraw the subject from the study for any reason at any time, without penalty or loss of benefits to which the subject is otherwise entitled. Neither the investigator, nor the trial staff, should coerce or unduly influence a subject to participate or to continue to participate in the trial. The extent of the confidentiality of subject records must be defined, and the subjects’ parents/guardians must be informed that applicable data protection legislation will be complied with. Subjects’ parents/guardians must be informed that the monitor(s), auditor(s), IRB/IEC, and the regulatory authority(ies) will be granted direct access to the subject's original medical records for verification of clinical trial procedures and/or data, without violating the confidentiality of the subject, to the extent permitted by the applicable laws and regulations and that, by signing a written informed consent form, the parent/guardian is authorizing such access. Subjects’ parents/guardians must be informed that records identifying the subject will be kept confidential, and to the extent permitted by the applicable laws and/or regulations, will not be made publicly available and, if the results of the trial are published, the subject’s identity will remain confidential.

The consent form generated by the investigator with the assistance of GlaxoSmithKline Biologicals, must be approved (along with the protocol, and any other necessary documentation) by the IRB/IEC and be acceptable to GlaxoSmithKline Biologicals. Consent forms must be in a language fully comprehensible to the prospective subjects’ parents/guardians. Informed consent shall be documented by the use of a written consent form approved by the IRB/IEC and signed/thumb-printed and dated by the parent/guardian, and by the person who conducted the informed consent discussion. The signature/thumb-print confirms the consent is based on information that has been understood. Each subject's signed/thumb-printed informed consent form must be kept on file by the investigator for possible inspection by Regulatory Authorities and/or GlaxoSmithKline Biologicals’ professional and Regulatory Compliance persons. The parent/guardian should receive a copy of the signed/thumb-printed and dated written informed consent form and any other written information provided to the subjects’ parents/guardians, and should receive copies of any signed/thumb-printed and dated consent form updates and any amendments to the written information provided to subjects’ parents/guardians. Oral witnessed consent will replace written consent only in countries where the local custom is contrary or if the subject’s incapacity precludes this and provided that the local legal obligations are fulfilled.

### Data Safety Monitoring Board (DSMB)

Details of the terms of reference (operating procedures) for the Data Safety Monitoring Board (DSMB) for studies with GlaxoSmithKline Biologicals’ candidate malaria vaccine are on file at GlaxoSmithKline Biologicals[[29]](#endnote-30).

#### Composition of the DSMB

An independent committee consisting of up to five experts in malaria, paediatrics, statistics and other appropriate disciplines will be appointed to oversee ethical and safety aspects of the study conduct.

#### Role of the DSMB

The role of the DSMB includes the review of the implementation and progress of the study. It provides initial, regular, and closing advice on safety-related issues to GSK Biologicals. Its advice is based on the interpretation of study data with reference to the study protocol.

The DSMB will confer before the initiation of the study (pre-initiation review), during the study and at the close out of the study to review any relevant safety data. ***The DSMB will review safety and reactogenicity data from a subset of the study population after each dose of vaccine to provide a recommendation for the administration of the subsequent dose (see section 5.2.4).*** When appropriate, the DSMB will also be asked to comment on data collection and efficacy endpoints corresponding to these data. They will review and approve the Report and Analysis Plan (RAP). Other unscheduled meetings may be required. Meetings must be documented and minutes made available for the study files on site and to the sponsors. The DSMB may, if deemed necessary, convene a meeting with or request further information from the Principal Investigators, the Medical Monitor/Local Safety Monitor, GSK Biologicals’ and MVI at PATH’s designated project representatives at any stage of the study.
**(Amended January 17, 2003)**

The DSMB must be informed by the investigator of:

- all subsequent protocol amendments, informed consent changes or revisions of other documents originally submitted for review
- serious and/or grade 3 adverse events (AEs) occurring during the study (transmitted through the Medical Monitor/Local Safety Monitor)
- all subsequent protocol modifications (for information)
- new information that may affect adversely the safety of the subjects or the conduct of the study.

The DSMB will be empowered to put the study on hold pending review of potential safety issues. All serious adverse events (SAEs), including death, will be reported to the DSMB, the Manager of Clinical Safety Vaccines at GSK Bio and the MVI by the Principal Investigator. All SAEs will be provided on an “as received” basis to the DSMB.

### Role of Local Safety Monitor

The overall role of the Local Safety Monitor (LSM), who will be an experienced clinician based in-country, will be to support the clinical investigators and to act as a link between the investigators and the DSMB. All SAEs will be reported to him/her in his/her capacity as a liaison to the DSMB. His/her involvement will be particularly important when decisions have to be made quickly. In exceptional circumstances, for example a death possibly related to vaccination, he/she will have the authority to suspend the whole or any specific aspect of the trial pending discussion with the DSMB.

The LSM’s role will include:

- acting as the study subject's advocate
- ongoing review of safety data and discussion with DSMB if necessary
- promptly communicating relevant safety information to the DSMB
- providing advice to the investigators on whether a set of clinical circumstances in a study warrants formal notification to the DSMB
- unblinding a subject if deemed necessary to allow for adequate treatment.
- performing and documenting routine site visits during the study.

The LSM will liaise closely with the chair of the DSMB throughout the course of the trial.

### Independent Study Monitors

Independent WHO monitors will provide additional oversight of the study.

## General study aspects

The DSMB (described in section 5.1.3) will be monitoring this study with regard to vaccine safety. The DSMB will receive the protocol prior to the initiation of the study and will liaise closely with the LSM throughout the course of this study.

### Screening and enrollment procedures

An overview of the recruitment plan is given in Appendix C.

Comprehension of the information contained within the Subject Information and Consent Form will be checked prior to screening by an oral interview with the subject’s parents/guardians, according to SOPs at CISM.

Only subjects with a written informed consent signed/thumb-printed and dated by parents or guardians will be screened. If consent is not available from both parents, the reason for the unavailability of one of the parents should be specified on the consent form. When appropriate, the informed consent should also be signed and dated by the oral witness.

Clinic forms will be prepared and filled in by the investigator at screening. Each form will contain the subject’s initials, permanent study area ID number, information about the subject’s date of birth, household, date of screening visit, whether the subject was enrolled or not and, where applicable, reasons for exclusion from the study based on medical history or physical or laboratory screening examination. All screening tests (see section 5.8.3) will be performed after informed consent has been obtained and before inclusion in the study. The investigator, as part of his routine source documentation will prepare a screening list based on the above information.

Children without healthcards will not be enrolled because their previous vaccination record is unknown. Neither will children with a twin be enrolled, to minimise the possibility of misidentification and misclassification during the study.

Blood samples will be taken by trained CISM laboratory workers according to CISM SOPs.

After reviewing the medical history, physical examination and laboratory results, any reasons for non-eligibility will be documented on the screening list. This list will be kept at the investigator’s site. Any clinically relevant finding will be treated appropriately by a physician. An overview of the laboratory assays can be found in section 5.8.3 and further details in CISM SOPs.

After reviewing the screening results, invitations will be delivered by fieldworkers to the parents/guardians of the children considered to be eligible for the study. The parents/guardians will be requested to bring their child to a designated area for vaccinations of the Manhiça District Hospital or Ilha Josina Health Facility on a specified day for the child to receive the first vaccine dose.

Each enrolled subject will receive an identification card with a photograph of the child and parent/guardian (photo ID card) and a Case Report Form (CRF) will be initiated. Inclusion of a parent/guardian in the photograph of each young child enrolled in this study will aid in recognition of this child. This photo ID card, specific for the study, and the subject’s CISM demographic surveillance system ID card will be used to identify subjects whenever they present via the morbidity surveillance system at Manhiça District Hospital or Ilha Josina Health Facility.

### Vaccination process

All vaccinations will take place at Manhiça District Hospital for Cohort 1 and at Ilha Josina Health Facility for Cohort 2. All vaccinations will be given by nurses or doctors. A physician experienced in the resuscitation of children will be available at all vaccination sessions. Facilities and equipment will be available to give emergency treatment in case of a rare anaphylactic reaction following administration of vaccines.

At each vaccination, the identity of the child will be confirmed using the photo ID card. On the day of the first vaccination the inclusion/exclusion criteria and consent form will be checked prior to vaccination. On the days of the subsequent vaccinations, elimination criteria and contraindications to vaccination will be checked prior to vaccination. A vaccine clinic form will be initiated, which will give the child’s identifiers and study number. This will be passed to the syringe fillers.

Since the vaccines used in this study are of distinct appearance, special precautions will be taken to ensure blinding. These procedures have been applied successfully in previous field studies, e.g. in The Gambia^7^. For each vaccination during the course of the study, a Syringe Filler and Vaccinator will work as a team: the Syringe Filler will prepare the vaccine for administration to a specific subject and the Vaccinator will administer the vaccine to each subject. To ensure blinding, the vaccinating teams will perform no other function in the study. The vaccine recipient and their parent/guardian as well as those responsible for the evaluation of safety or immunogenicity study parameters will all be unaware which vaccine preparation was administered to a particular subject.

The Syringe Filler will select a sealed box, labelled with the study subject number and containing a numbered vial of the appropriate vaccine, and will fill a syringe according to SOP. The Syringe Filler will then place an opaque label with the subject's study number on the syringe. Only then will he/she pass the filled masked syringe and the vaccine clinic form to the Vaccinator.

After the Vaccinator receives the filled masked syringe and the vaccine clinic form, he/she will again check the subject’s identity with the vaccine clinic form.

Each subject will be administered only the vaccine corresponding to his/her study number.

The RTS,S/AS02A candidate malaria vaccine will be administered according to 0, 1, 2-month vaccination schedule, intramuscularly in the deltoid region of alternating arms. The hepatitis B vaccine (Engerix-B™) will be administered to control group children of ***24*** ~~18~~ months and above, according to 0, 1, 2-month vaccination schedule, intramuscularly in the deltoid region of alternating arms. Children of less than ***24*** ~~18~~ months in the control group will be vaccinated according to 0, 1, 2-month vaccination schedule, intramuscularly in the deltoid region of alternating arms. These children will receive two doses of the 7-valent pneumococcal conjugate vaccine (Prevnar®) at the first and third vaccination and one dose of or *Haemophilus influenzae* type b vaccine (Hiberix™) at the second vaccination. Should any local impairment prevent administration of the vaccine dose into the upper arm foreseen for that particular dose, the vaccine may be administered into the opposite arm (deltoid region).
**(Amended January 17, 2003)**

Paediatric dose (0.25 ml dose volume) of the RTS,S/AS02A candidate malaria vaccine will be administered at each vaccination. Full doses (0.5 ml dose volume) of hepatitis B vaccine (Engerix-B™), the 7-valent pneumococcal conjugate vaccine (Prevnar®) and *Haemophilus influenzae* type b vaccine (Hiberix™) will be given to the control group.

Subjects who cannot be vaccinated on the originally scheduled date due to an acute illness (on the judgement of the investigator or designate) or scheduling conflicts will be vaccinated within 7 days and undergo all study procedures for this visit on the same day as vaccination. In the particular case of any child found to be febrile (axillary temperature ≥  37.5°C), a blood slide will be taken to investigate malaria parasitaemia. Children will be treated as appropriate for their condition and will be followed up until resolution of any symptoms and be vaccinated if their clinical symptoms resolve within 7 days.

Those who cannot be re-vaccinated within 7 days of their scheduled date will continue all study procedures apart from receiving further study vaccinations.

### Safety and reactogenicity assessment during 4-day post-vaccination follow-up

On the day of vaccination, all subjects will remain at the vaccination site after vaccination under the supervision Principal Investigator or other designated staff, who will record any adverse events that should occur, according to SOPs. Each vaccinee will be closely observed for one hour or longer if necessary with appropriate medical treatment readily available in case of a rare anaphylactic reaction following the administration of the vaccine. After the observation period, axillary body temperature will be recorded again and, together with any local and general signs and symptoms that may occur, will be recorded in the CISM clinic form by the investigator. Trained staff, drugs and equipment are available in the event of a child collapsing. Trained field workers, under the supervision of the Principal Investigator, will visit each subject at daily intervals for three subsequent days following each vaccine dose (detailed in CISM SOPs). At each visit, the axillary temperature will be taken and recorded on diary cards together with any solicited adverse events. Analgesics are provided to field workers for the treatment of children with injection site pain. Parents will not routinely be provided with these medications. In the event that the field worker finds any severe general solicited or unsolicited sign or symptom or serious adverse event, the subject will be brought to Manhiça District Hospital or Ilha Josina Health Facility for examination by the Principal Investigator or other designated staff. The corresponding clinical data, including treatment provided, will be documented on diary cards and CISM clinic forms and transcribed onto the CRF. Any SAEs will be reported to the DSMB (through the LSM), GSK Biologicals and MVI (see section 8.5.2). Unresolved AEs will be followed-up by field workers until resolution under the supervision of the Principal Investigator and data will be entered onto the diary cards. Similarly, a child with a fever or history of fever will be referred to Manhiça Hospital or Ilha Josina Health Facility for assessment via the morbidity surveillance system.

Diary cards will be checked and verified by the Principal Investigator before transcription onto CRFs after the 3-day field worker follow-up. The Principal Investigator or Co-investigators will check all records on a daily basis and any recorded general adverse event of intensity grade 3 will be checked by one of the CISM physicians. For this purpose, a field worker will be sent to the parent/guardian of the child and ask the parent/guardian to bring the child to Manhiça Hospital or Ilha Josina Health Facility. If necessary, CISM will arrange transport for this visit. Any local adverse event of intensity grade 3 will be notified to the Principal Investigator or Co-investigators if this event has not resolved or diminished in intensity over the 3-day field worker follow-up.

The Principal Investigator has a primary responsibility for the data transcribed onto the CRFs. The Principal Investigator, together with the field worker supervisor, will perform checks to determine that visits are made as required and findings are correctly recorded (as specified in CISM SOPs ) and this will be documented.

### *DSMB review of safety and reactogenicity data per vaccine dose*

***The DSMB will review the reactogenicity and safety data in a subset of the study population after each vaccination, according to procedures specified in CISM SOPs. Briefly, the DSMB will review the blinded safety and reactogenicity data pertaining to the first week post-vaccination of the first 200 children vaccinated. Following the evaluation of these data, the DSMB will make their recommendation for the administration of the subsequent dose of vaccine to proceed. This review will be repeated after each dose.***
**(Amended January 17, 2003)**

### Morbidity surveillance (all subjects)

Morbidity surveillance has been in place at Manhiça District Hospital since 1996 and a similar system will be put in place in Ilha Josina Health Facility for the purposes of this trial (a detailed review of the level of health care service is given in sections 1.1.6 and 1.1.7). These two health facilities are the source of primary care for this area. The surveillance system will provide a comprehensive recording of all outpatient attendances, the investigational results, diagnosis and management. Prior to the start of the study, all parents/guardians of study children will be educated on the appropriate action they should take if their child becomes unwell at any time during the study period. They will be asked not to medicate their child at home, but to seek medical care at either Manhiça District Hospital or Ilha Josina Health Facility.

Study staff (a nurse, medical agent or field worker), will be available 24 hours per day at both health facilities to receive and identify study participants when they present and to ensure complete investigation and documentation of the attendance. For all children attending outpatients within the age range of the trial, they will ask the carer if the child is in this study and request to see the child’s vaccine study identity card and CISM demographic surveillance system ID card (see section 5.2.1 for details of identity cards). If the identity cards are not available, the identity of the child will be confirmed against details held at the health facilities.

The child will then be referred for evaluation by the medical assistant or medical doctor, who will follow CISM SOPs for the examination, investigation and documentation of each presentation. This information will be recorded on a CISM clinic morbidity surveillance questionnaire. This questionnaire will provide a record of the child’s name and study number, key symptoms and signs, axillary temperature, results of the laboratory tests and imaging examinations available at the time of form completion and the diagnosis, the treatment prescribed and will state whether hospital admission was required.

These forms will be retained by the study personnel in the health facility until they are sent to CISM at the end of each working day. At CISM, the questionnaires will be reviewed by the Principal Investigator or delegate. If it is necessary to notify any of these consultations as a serious adverse event, the Principal Investigator or delegate will review the child as necessary and complete an SAE form to notify the event (as specified in section 8.5).

### Passive case detection of clinical cases of malaria (all subjects)

If a child presenting to Manhiça District Hospital or Ilha Josina Health Facility reports fever within the preceding 24 hours or has a documented fever (defined as axillary temperature ≥ 37.5ºC) the same procedure described above (section 5.2.4) will be followed. In addition, blood will be collected for determination of malaria parasites and haematocrit by the CISM laboratory staff. Duplicate blood slides will be taken and labeled with the same unique laboratory ID number, which will also be recorded on the CISM clinic morbidity surveillance questionnaire. Both slides will be taken to the CISM Laboratory and stained with Giemsa. One will be read immediately (within 2 hours) and the result reported to guide diagnosis and management. The second slide will be kept for a later reading, according to CISM SOPs, and will determine parasite density for data analysis. Both slides will be stored at CISM. In addition, blood samples will be taken and stored for parasite genotyping.

Passive case detection of clinical cases of malaria in both cohorts will start from the day of first vaccination (day 0) and will continue until study end (month 21). For the purposes of analysis, data obtained from passive case detection from day 0 onwards will be used for an intention-to-treat analysis for vaccine efficacy while the surveillance period with respect to the primary, secondary and tertiary endpoints will start from 14 days post dose 3 (day 74).

The clinical case definition for malaria currently used at Manhiça District Hospital and Ilha Josina Health Facility is a history of fever within the previous 24 hours or documented fever (temperature ≥ 37.5°C) at the time of presentation plus any level of asexual *Plasmodium falciparum* parasitaemia. This will not be changed for the trial and all cases meeting this case definition will receive treatment for malaria.

Axillary temperature measurements will be taken throughout this study. It is widely used in the health care provision system because it does not require sterilization of equipment between use. In addition, rectal temperature measurement is not acceptable in many African cultures. Axillary temperature measurement has long been used in malaria research in Africa: consequently, the disease rates and case definitions on which this trial has been designed have used axillary temperatures. It is therefore appropriate that this method continues to be used in this trial.

### Surveillance for malaria infection (Cohort 2)

Four weeks prior to the start of surveillance for malaria infection (i.e. 2 weeks before the third vaccination), asymptomatic parasitaemia will be cleared in children enrolled into Cohort 2 with amodiaquine-SP (sulfadoxine and pyrimethamine : 25 mg/kg body weight p.o. and 1.25 mg/kg body weight p.o. single dose; amodiaquine: 10 mg/kg body weight p.o. once daily for three days). The prevalence of asymptomatic parasitaemia in the study area is high, therefore all children will receive a drug for clearance of malaria parasites without prior blood sampling to determine parasitaemia. The first dose of amodiaquine-SP will be administered at a field worker visit 2 weeks before the third dose of vaccine (day 46). The absence of parasitaemia will be checked by taking blood for a blood slide at clinic visit 4 (day 60). Any child with any level of parasitaemia at this point will be treated with a second-line treatment for malaria and will not undergo the surveillance period for active detection of infection. All such children will not contribute to the endpoint for vaccine efficacy in Cohort 2.

The surveillance period for malaria infection will begin 14 days after the third dose of vaccine (day 74). Surveillance will continue with the clinic visit on day 90 and then field worker visits every two weeks (one visit every 14 days) for the following 2½ months (six visits) and then monthly for 2 months (two visits at months 7 and 8), and ending with the clinic visit at month 8½. Blood samples will be taken for examination of malaria parasitaemia and parasite genotyping at each visit. The first blood slide examination will be made on the first day of surveillance, i.e. 14 days after the third dose (day 74). At first detection of malaria infection with asexual forms of *P. falciparum* in a child, either by field worker surveillance or on clinical presentation with malaria disease at a health facility, the child will be considered to have met the endpoint for vaccine efficacy in Cohort 2 and no further visits for surveillance of infection will be undertaken. The child will still be under morbidity surveillance and will receive monthly visits by field workers for SAE surveillance and will also take part in cross-sectional surveys at month 8½ and month 21.

For the surveillance period for malaria infection, a field worker visit will consist of visiting the child at home and completing a brief surveillance for infection morbidity questionnaire, which will include the reporting of malaria symptoms and a record of axillary temperature. If the child is well and afebrile, blood will be taken by finger prick and blood will be taken for examination of malaria parasitaemia and parasite genotyping. These blood slides will be stained with Giemsa and read to determine the presence of parasites at Ilha Josina Health Facility. Treatment for children not considered to be clinically ill will be sent by a field worker to all blood-slide positive children within 36 hours of the original blood slide being taken.

In the event that a child is reported as having a history of fever within the preceding 24 hours or has a documented axillary temperature of 37.5°C or greater, no blood slides will be taken by the field worker but transport will be arranged by a project vehicle to Ilha Josina Health Facility. The surveillance for infection morbidity questionnaire will record that the child was transferred to Ilha Josina Health Facility. There the child will be reviewed by a clinician or medical agent or assistant, who will complete the CISM clinic morbidity surveillance questionnaire and the child will be managed according to the procedures detailed above (see section 5.2.5) and a finger blood sample will be taken for determination of parasitaemia and parasite genotyping. If parasitaemia is detected in the blood slides taken on arrival at Ilha Josina Health Facility, the child will be treated as appropriate.

For data analysis purposes related to the study, all slides will be transferred to CISM for parasite density reading and storage.

### Monthly home visits to detect SAEs (all subjects)

Field workers will visit each child once every month for 18 months starting from 60 days after last vaccination (day 120) to maintain contact with the subject and his family and to ensure the complete detection of SAEs. The information will also be used for subjects in Cohort 1 to determine period of residence within the study region and hence the period at risk. Absences of 2 weeks or more in duration will be recorded in multiples of 1 week.

These home visits are not designed to monitor the morbidity in the study population. However, if any child is reported to be unwell at the time of a visit, the field worker will advise the parents/guardians to seek care at either Manhiça District Hospital or Ilha Josina Health Facility. In the event that a child is seriously ill the field worker will inform the Principal Investigator or designate and transport arranged to Manhiça District Hospital or Ilha Josina Health Facility, if judged appropriate by the responsible clinician.

During the field worker visits, the children’s parents/guardians will be asked retrospectively if any SAEs occurred since the last visit and this information will be recorded. Unreported SAEs detected in this way will be investigated and reported by the Principal Investigator or delegate on the corresponding SAE forms (see Section 8.5.2). In the case of a death, supplementary information will be gained using the verbal autopsy technique. This will be conducted according to previously published methods[[30]](#endnote-31) and detailed in the SOPs on file with the investigators.

### Cross sectional surveys (all subjects)

All study children will be reviewed at two surveys (months 8½ and 21) to determine safety, immunogenicity and efficacy endpoints. An additional survey will be performed at day 90 (30 days post-dose 3) to determine safety and immunogenicity endpoints only. Parents/guardians will be asked to bring their child to either Manhiça Hospital or Ilha Josina Health Facility on a given day for each cross-sectional survey.

Observations at cross-sectional surveys at months 8½ and 21 will include axillary temperature, spleen size (classified according to Hackett’s Scale, see section 5.7) and body weight. A history will be taken of bednet usage, which will include use of impregnation with insecticides. At all cross-sectional surveys, all subjects will have blood samples taken for:

- Day 90: haematology (blood count) and biochemistry and anti-CS antibodies (anti-HBs antibodies, parasite genotyping and blood slide examination for malaria parasites, if Cohort 2).
- Month 8½: haematology (haematocrit) and biochemistry, blood slide examination for malaria parasites, ***parasite genotyping*** and anti-CS antibodies (~~parasite genotyping and~~ anti-HBs antibodies if Cohort 2). **(Amended August 13, 2002)**
- Month 21: haematology (haematocrit) and biochemistry, blood slide examination for malaria parasites and anti-CS antibodies (anti-HBs antibodies if Cohort 2).

### Medical treatment for all study subjects during the study period

A detailed description of the facilities available at Manhiça Hospital and Ilha Josina Health Facility are detailed in the sections1.1.6 and 1.1.7 and section 5.2.4 for morbidity surveillance. The treatment for medical conditions will be given according to the standard treatment regimes of Mozambique.

The Mozambican health services ask that the parents/guardians contribute to the costs of the child’s care, this is a standard fee of 500 Mozambican Meticais (about 0.001 US$) for outpatient medication. However, those families who cannot afford to pay will receive treatment free of charge. Care and medication given during hospitalization are free.

Manhiça Hospital is able to provide a wide range of services and thus the need for referral to the specialist paediatric hospital in Maputo is rare. If this is considered necessary by the Principal Investigator or designate, CISM will facilitate the transfer of study subjects to Maputo Paediatric Hospital.

### Management and treatment of malaria in all subjects

Children presenting with uncomplicated malaria not requiring parental treatment will receive treatment with combination therapy of amodiaquine and sulfadoxine-pyrimethamine (SP) under supervision at the health care facility and will be managed according to standard medical practice.

The dosage of amodiaquine-SP to be used will be:

- single dose of sulfadoxine (25 mg/kg body weight p.o.) and pyrimethamine (1.25 mg/kg body weight p.o.)
- amodiaquine: 10 mg/kg body weight p.o. once daily for three days.

All dosages will be rounded to the nearest quarter tablet.

All children requiring parenteral treatment or with complicated malaria will be admitted to Manhiça Hospital and will receive quinine. Full details of malaria case management are contained in an SOP at the investigator’s site.

### Cross-over immunisation at study end

***One of the aims of Cohort 2 is a formal comparison of the immune response to the HBsAg of RTS,S/AS02A to Engerix-B™ (see sections 10.6.2 and 10.7.4). If non-inferiority of the seroprotective anti-HBs response induced by RTS,S/AS02A, as compared to that afforded by Engerix-B™, is demonstrated in the statistical comparison, then no cross-over immunisation of the malaria vaccine group with the Engerix-B™ control vaccine is planned. However, if non-inferiority is not demonstrated, the following rescue plan for all recipients of RTS,S/AS02A will be initiated at study end (month 21) but will not be considered as a study procedure and therefore will not be documented in the CRF. All children of 24 months or more at first vaccination in the RTS,S/AS02A group will be offered, at study end, a blood test to determine their anti-HBs antibody titre. Those children with a titre below the protective titre of 10 mIU/mL will be offered by the investigator one or more doses of Engerix-B™ (commercial presentation) to assure seroprotective levels are attained.*** ~~as the RTS,S/AS02A malaria vaccine contains the same antigen that is in Engerix‑B™. However,~~ In addition, any subject of ***24*** ~~18~~ months or more at first vaccination that cannot be vaccinated within 7 days of the originally scheduled date for vaccination, will be offered at the end of the study additional doses of Engerix-B™ (commercial presentation) under the responsibility of the investigator.
**(Amended January 17, 2003)**

Cross-over immunisation of the malaria vaccine group with Prevnar® and Hiberix™ will be performed at the end of the study (month 21). All subjects in the RTS,S/AS02A group and of less than ***24*** ~~18~~ months old at first vaccination will be offered immunisation with Prevnar® and Hiberix™ (commercial presentations). Children will be aged 33‑***45*** ~~39~~ months at this time and a single dose of both vaccines will be given in line with the standard recommendations for children of this age.
**(Amended January 17, 2003)**

Cross-over immunisation will offered by the investigator at the end of the study and will not be considered as a study procedure and therefore will not be documented in the CRF.

## Outline of study procedures for Cohort 1

| **Study phase** | **Screening (Amended January 17, 2003)** | **Double-blind phase** | | | | | | | | | **Single-blind phase** | |
| --- | --- | --- | --- | --- | --- | --- | --- | --- | --- | --- | --- | --- |
|  |  | **Vaccination** | | | | | | **Efficacy Surveillance** | | |  |  |
| **Months** |  | **0** | | **1** | | **2** | | **3** | **4-8** | **8½** | **9-20** | **21** |
| **Days** | ***–90 to –7*** *~~35 to -7~~* | ***0*** | *1-3* | *30* | *31-33* | *60* | *61-63* | *90* |  | *256* |  | *630* |
| **Clinic Visit** | **1** | **2** |  | **3** |  | **4** |  | **5** |  | **6** |  | **7** |
| ***Field worker visit*** |  |  | *1-3* |  | *4-6* |  | *7-9* |  | *10-14* |  | *15-26* | *27* |
| **Study Procedures** |  |  | | | | | | | | |  | |
| Informed consent | ● |  |  |  |  |  |  |  |  |  |  |  |
| Check of inclusion/exclusion criteria |  | ● |  |  |  |  |  |  |  |  |  |  |
| Medical history |  | ● |  |  |  |  |  |  |  |  |  |  |
| Physical examination |  | ● |  |  |  |  |  |  |  |  |  |  |
| Measurement of ***anthropometry**** ~~body weight~~ **(Amended August 13, 2002) (Amended January 17, 2003)** | ***●*** | ~~●~~ |  |  |  |  |  |  |  | ● |  | ● |
| Check of contraindications to vaccination |  |  |  | ● |  | ● |  |  |  |  |  |  |
| Body temperature |  | ● |  | ● |  | ● |  |  |  | ● |  | ● |
| Randomization |  | ● |  |  |  |  |  |  |  |  |  |  |
| Vaccination |  | ● |  | ● |  | ● |  |  |  |  |  |  |
| Check of elimination criteria |  |  |  | ● |  | ● |  | ● |  | ● |  | ● |
| Recording of medication |  | ● | ● | ● | ● | ● | ● | ● | ● | ● | ● | ● |
| **Safety Data Collection** |  |  | | | | | | | | |  | |
| Post-vaccination recording of solicited symptoms by investigator |  | ● |  | ● |  | ● |  |  |  |  |  |  |
| Daily post-vaccination recording of solicited symptoms by field workers |  |  | ● |  | ● |  | ● |  |  |  |  |  |
| Recording of unsolicited AEs up to 1 month (min. 30 days) post-vaccination by investigator |  |  |  | ● |  | ● |  | ● |  |  |  |  |
| Morbidity surveillance/Recording of SAEs |  | ● | ● | ● | ● | ● | ● | ● | ● | ● | ● | ● |
| Monthly home visits |  |  |  |  |  |  |  |  | ● | ● | ● | ● |
| **Efficacy data collection** |  |  | | | | | | | | |  | |
| Passive case detection** |  | ● | ● | ● | ● | ● | ● | ● | ● | ● | ● | ● |
| Measurement of spleen size (cross-sectional surveys) **(Amended January 17, 2003)** | ***●*** | ~~●~~ |  |  |  |  |  |  |  | ● |  | ● |
| Recording of bednet use |  | ● |  |  |  |  |  |  |  | ● |  |  |
| **Laboratory analyses** |  |  | | | | | | | | |  | |
| Finger prick sampling^1^ | ● | ● |  |  |  |  |  | ● |  | ● |  | ● |
| Complete blood count^2^ | ● |  |  |  |  |  |  | ● |  |  |  |  |
| Haematocrit | ● |  |  |  |  |  |  | ● |  | ● |  | ● |
| Creatinine, ALT, bilirubin | ● |  |  |  |  |  |  | ● |  | ● |  | ● |
| Blood film for parasitaemia determination |  |  |  |  |  |  |  |  |  | ● |  | ● |
| HBsAg |  | ● |  |  |  |  |  |  |  |  |  |  |
| Anti-CS antibodies |  | ● |  |  |  |  |  | ● |  | ● |  | ● |
| IFAT (Indirect fluorescent antibody test) | ● |  |  |  |  |  |  |  |  |  |  |  |
| ***Parasite genotyping* (Amended August 13, 2002)** |  |  |  |  |  |  |  |  |  | ***●*** |  |  |

● indicates a study procedure that requires documentation in the CRF and  indicates a study procedure that does not require documentation.

****: Measurement of body weight and height or length, as appropriate for age, at screening (Clinic visit 1) ~~on day 0 (Clinic visit 2~~). Measurement of body weight on Month 8½ and Month 21 (Clinic visits 6 and 7)* (Amended August 13, 2002) (Amended January 17, 2003)**

**: If a child presenting to Manhiça District Hospital reports fever within the preceding 24 hours or has a documented fever (axillary temperature ≥ 37.5°C) then blood will be taken for parasitaemia determination, haematocrit and a blood sample will be kept for possible PCR analysis of parasite genotype.

Double line after Month 8½ indicates the end of the surveillance period for primary objective (double-blind phase) and Study Conclusion. Thereafter, safety follow-up field worker visits at monthly intervals and passive surveillance will continue and one remaining cross-sectional survey will be performed at Month 21.

It is the investigator’s responsibility to ensure that the intervals between visits/contacts are strictly followed. These intervals determine a subject’s evaluability in the according to protocol analyses (see Sections 4.5 and 10.5 for details of criteria for evaluability and cohorts to be analyzed).

Intervals between study visits in Passive Case Detection Cohort

| **Interval for clinic visits** | **Duration of interval** |
| --- | --- |
| 1 (Visit 1 →Visit 2) | Screening ***90*** ~~35~~ to 7 days before first vaccination visit |
| 2 (Visit 2 →Visit 3) | 27-37 days |
| 3 (Visit 3 →Visit 4) | 27-37 days |
| 4 (Visit 4 →Visit 5) | 30-37 days |
| 5 (Visit 5 →Visit 6) | 146-186 days |
| 6 (Visit 5→Visit 7) | 512-568 days |

**(Amended January 17, 2003)**

## Detailed description of study stages/visits for Cohort 1

**When materials are provided by GlaxoSmithKline Biologicals, it is mandatory that all clinical samples (including serum samples) be collected using exclusively those materials in the appropriate manner. The use of other materials could result in the exclusion of the subject from analysis. The investigator must ensure that his/her personnel and the laboratory(ies) under his/her supervision comply with this requirement.**

The parents/guardians will be instructed to contact the investigator immediately should the child manifest any signs or symptoms any time throughout the study period that the parents/guardians perceive as serious.

| **Clinic Visit 1: Screening** | | |
| --- | --- | --- |
| **Days *–90 to -7* ~~35 to -7~~: Screening (Amended January 17, 2003)** | | |
| · | | Obtain written/thumb-printed informed consent from the parents/guardians. |
| · | | History taking and physical examination ***– baseline measurement of spleen size, of body weight and height or length, as appropriate for age* (Amended January 17, 2003)** |
| · | | Checking of inclusion/ exclusion criteria. |
| · | | Finger prick blood sample collected (1-2 ml, providing a minimum of 0.5 ml of serum) for baseline analysis of:   - Haematology : Blood Count (BC) which includes platelets, white blood cells, haematocrit and haemoglobin - Biochemistry (creatinine, bilirubin and ALT) - Indirect fluorescent antibody test (IFAT) for anti-parasite antibodies |
| **Clinic Visit 2: First vaccination (Day 0)** | | |
| **Day 0: Pre vaccination 1** | | |
| · | | Provision of photo ID |
| · | | Checking of inclusion/ exclusion criteria. |
| · | | History-related physical examination – pre-vaccination axillary body temperature.~~, baseline measurement of spleen size., body weight~~ ***~~and height or length, as appropriate for age~~* (Amended August 13, 2002) (Amended January 17, 2003)** |
| · | | Randomization |
| · | | Recording of concomitant medication and bednet use for the previous night. |
| · | | Finger prick blood sample collected pre-vaccination (1-2 ml, providing a minimum of 0.5 ml of serum) for baseline analysis of:   - Serology (HBsAg and anti-CS antibodies) |
| · | | Intra-muscular (IM) administration of the first vaccine dose in the deltoid region of the left arm. |
| · | | Each vaccinee will be closely observed for one hour or longer if necessary with appropriate medical treatment readily available in case of a rare anaphylactic reaction following the administration of the vaccine. After the observation period, axillary body temperature will be recorded again, and together with any local and general signs and symptoms that may occur, will be recorded in the CRF by the investigator. |
| ***Field worker vaccination follow-up visits 1-3 after first vaccination*** | | |
| ***Days 1, 2 and 3:*** | | |
| · | | Recording of concomitant medication. |
| · | Reporting of any Serious Adverse Events (SAEs) which the vaccinee has experienced since last visit. | |
| · | | Recording of axillary body temperature and any local or general solicited symptoms experienced at time of field worker visit, and within the previous 24 hours, onto diary cards by field workers, on the 3 subsequent days after vaccination. |
| **Clinic Visit 3: Second vaccination (27 to 37 days after Visit 2)** | | |
| **Day 30: Post vaccination 1** | | |
| · | Verification of photo ID and subject number | |
| · | The investigator must record all non-serious adverse events that occur 1 month (minimum 30 days) post-vaccination 1. | |
| · | Reporting of any Serious Adverse Events (SAEs) which the vaccinee has experienced since the first vaccine dose. | |
| · | Physical examination: recording of pre-vaccination axillary body temperature. | |
| · | Checking of elimination criteria and contraindications to vaccination. | |
| · | Recording of concomitant medication. | |
| · | Intra-muscular (IM) administration of the second vaccine dose in the deltoid region of the right arm. | |
| · | Each vaccinee will be closely observed for one hour or longer if necessary with appropriate medical treatment readily available in case of a rare anaphylactic reaction following the administration of the vaccine. After the observation period, axillary body temperature will be retaken, and together with local and general signs and symptoms that may occur, will be recorded in the CRF by the investigator. | |
| ***Field worker vaccination follow-up visits 4-6 after second vaccination*** | | |
| ***Days 31, 32 and 33:*** | | |
| · | Recording of concomitant medication. | |
| · | Reporting of any Serious Adverse Events (SAEs) which the vaccinee has experienced since last visit. | |
| · | | Recording of axillary body temperature and any local or general solicited symptoms experienced at time of field worker visit, and within the previous 24 hours, onto diary cards by field workers, on the 3 subsequent days after vaccination. |
| **Clinic Visit 4: Third vaccination (27 to 37 days after Visit 3)** | | |
| **Day 60: Post vaccination 2** | | |
| · | Verification of photo ID and subject number | |
| · | The investigator must record all non-serious adverse events that occur 1 month (minimum 30 days) post-vaccination 2. | |
| · | Reporting of any Serious Adverse Events (SAEs) which the vaccinee has experienced since the previous vaccine dose. | |
| · | Physical examination: recording of pre-vaccination axillary body temperature . | |
| · | Checking of elimination criteria and contraindications to vaccination. | |
| · | Recording of concomitant medication. | |
| · | Intra-muscular (IM) administration of the third vaccine dose in the deltoid region of the left arm. | |
| · | Each vaccinee will be closely observed for one hour or longer if necessary with appropriate medical treatment readily available in case of a rare anaphylactic reaction following the administration of the vaccine. After the observation period, axillary body temperature will be retaken, and together with local and general signs and symptoms that may occur, will be recorded in the CRF by the investigator. | |
| ***Field worker vaccination follow-up visits 7-9 after third vaccination*** | | |
| ***Days 61, 62 and 63:*** | | |
| · | Recording of concomitant medication. | |
| · | Reporting of any Serious Adverse Events (SAEs) which the vaccinee has experienced since last visit. | |
| · | | Recording of axillary body temperature and any local or general solicited symptoms experienced at time of field worker visit, and within the previous 24 hours, onto diary cards by field workers, on the 3 subsequent days after vaccination. |
| **Clinic Visit 5: Follow-up visit after third vaccination and Cross-sectional Survey (30 to 37 days after Visit 4)** | | |
| **Day 90: Post vaccination 3** | | |
| · | Verification of photo ID and subject number | |
| · | Checking of elimination criteria. | |
| · | Recording of concomitant medication. | |
| · | The investigator must record all non-serious adverse events that occur 1 month (minimum 30 days) post-vaccination 3. | |
| · | Reporting of any Serious Adverse Events (SAEs) which the vaccinee has experienced since the previous vaccine dose. | |
| · | | Physical examination |
| · | | Finger prick blood sample collected (1-2 ml, providing a minimum of 0.5 ml of serum) for analysis of:   - Serology (anti-CS antibodies) - Haematology : Blood Count (BC) which includes platelets, white blood cells, haematocrit and haemoglobin - Biochemistry (creatinine, bilirubin and ALT) |
| ***Field worker monthly follow-up visits 10-14*** | | |
| ***Months 4 to 8:*** | | |
| · | Reporting of any Serious Adverse Events (SAEs) which the vaccinee has experienced since last visit. | |
| · | Recording of concomitant medication and demographic data collection. | |
| **Clinic Visit 6: Cross-sectional Survey at Month 8½ (146 to 186 days after Visit 5)** | | |
| **Month 8½:** | | |
| · | Verification of photo ID and subject number | |
| · | Checking of elimination criteria. | |
| · | Reporting of any Serious Adverse Events (SAEs) which the vaccinee has experienced since the last visit. | |
| · | Recording of concomitant medication. | |
| · | | Evaluation of spleen size (classification) and body weight |
| · | Recording of axillary body temperature. | |
| · | | Recording of bednet use for the previous night |
| · | | Finger prick blood sample collected (1-2 ml, providing a minimum of 0.5 ml of serum) for analysis of:   - Serology (anti-CS antibodies) - Haematology : haematocrit - Biochemistry (creatinine, bilirubin and ALT) - Blood film for parasitaemia determination - ***Parasite genotyping***   **(Amended August 13, 2002)** |
| · | | End of surveillance period for primary objective (double-blind phase) and Study Conclusion |
| ***Field worker monthly follow-up visits 15-27*** | | |
| ***Months 9 to 21:*** | | |
| · | Reporting of any Serious Adverse Events (SAEs) which the vaccinee has experienced since last visit. | |
| · | Recording of concomitant medication and demographic data collection. | |
| **Clinic Visit 7: Cross-sectional Survey at Month 21 (512 to 568 days after Visit 5)** | | |
| **Month 21:** | | |
| · | Verification of photo ID and subject number | |
| · | Checking of elimination criteria. | |
| · | Reporting of any Serious Adverse Events (SAEs) which the vaccinee has experienced since the last visit. | |
| · | Recording of concomitant medication. | |
| · | | Evaluation of spleen size (classification) and body weight |
| · | Recording of axillary body temperature. | |
| · | | Finger prick blood sample collected (1-2 ml, providing a minimum of 0.5 ml of serum) for analysis of:   - Serology (anti-CS antibodies) - Haematology : haematocrit - Biochemistry (creatinine, bilirubin and ALT) - Blood film for parasitaemia determination |
| · | | End of follow-up period for safety and passive surveillance and Study Conclusion of single-blind surveillance phase. |

## Outline of study procedures for Cohort 2

| **Study phase** | | **Screening (Amended January 17, 2003)** | | **Double-blind phase** | | | | | | | | | | | | | | | | | | | | | | | **Single-blind phase** | |
| --- | --- | --- | --- | --- | --- | --- | --- | --- | --- | --- | --- | --- | --- | --- | --- | --- | --- | --- | --- | --- | --- | --- | --- | --- | --- | --- | --- | --- |
|  |  |  |  | **Vaccination** | | | | | | | | | | | | | | **Efficacy Surveillance** | | | | | | | | |  |  |
| **Months** | |  | | **0** | | | | **1** | | | | | | **2** | | | | | | **3** | | **3-6** | | **7-8** | | **8½** | **9-20** | **21** |
| **Days** | | ***-90 to –7***  *~~-35 to -7~~* | | ***0*** | | *1-3* | | *30* | | *31-33* | | *46* | | *60* | | *61-63* | | *74* | | *90* | |  | |  | | *256* |  | *630* |
| **Clinic Visit** | | **1** | | **2** | |  | | **3** | |  | |  | | **4** | |  | |  | | **5** | |  | |  | | **6** |  | **7** |
| ***Field worker visit*** | |  | |  | | *1-3* | |  | | *4-6* | | *7* | |  | | *8-10* | | *11* | |  | | *12-17* | | *18-19* | |  | *20-31* | *32* |
| **Study Procedures** | |  | | | | | | | | | | | | | | | | | | | | | | | | | | |
| Informed consent | | ● | |  | |  | |  | |  | |  | |  | |  | |  | |  | |  | |  | |  |  |  |
| Check of inclusion/exclusion criteria | |  | | ● | |  | |  | |  | |  | |  | |  | |  | |  | |  | |  | |  |  |  |
| Medical history | |  | | ● | |  | |  | |  | |  | |  | |  | |  | |  | |  | |  | |  |  |  |
| Physical examination | |  | | ● | |  | |  | |  | |  | |  | |  | |  | |  | |  | |  | |  |  |  |
| Measurement of ***anthropometry*** ~~body weight~~ **(Amended August 13, 2002) (Amended January 17, 2003)** | | ***●*** | | ~~●~~ | |  | |  | |  | |  | |  | |  | |  | |  | |  | |  | | ● |  | ● |
| Check of contraindications to vaccination | |  | |  | |  | | ● | |  | |  | | ● | |  | |  | |  | |  | |  | |  |  |  |
| Body temperature | |  | | ● | |  | | ● | |  | |  | | ● | |  | | ● | | ● | | ● | | ● | | ● |  | ● |
| Randomisation | |  | | ● | |  | |  | |  | |  | |  | |  | |  | |  | |  | |  | |  |  |  |
| Vaccination | |  | | ● | |  | | ● | |  | |  | | ● | |  | |  | |  | |  | |  | |  |  |  |
| Check of elimination criteria | |  | |  | |  | | ● | |  | |  | | ● | |  | |  | | ● | |  | |  | | ● |  | ● |
| Recording of medication | |  | | ● | | ● | | ● | | ● | |  | | ● | | ● | |  | | ● | |  | |  | | ● | ● | ● |
| **Safety Data Collection** |  | |  | |  | |  | |  | |  | |  | |  | |  | |  | |  | |  | |  | |  |  |
| Post-vaccination recording of solicited symptoms by investigator | |  | | ● | |  | | ● | |  | |  | | ● | |  | |  | |  | |  | |  | |  |  |  |
| Daily post-vaccination recording of solicited symptoms by field workers | |  | |  | | ● | |  | | ● | |  | |  | | ● | |  | |  | |  | |  | |  |  |  |
| Recording of unsolicited AEs up to 1 month (min. 30 days) post-vaccination by investigator | |  | |  | |  | | ● | |  | |  | | ● | |  | |  | | ● | |  | |  | |  |  |  |
| Morbidity surveillance/Recording of SAEs | |  | | ● | | ● | | ● | | ● | |  | | ● | | ● | | ● | | ● | | ● | | ● | | ● | ● | ● |
| Monthly home visits for SAEs | |  | |  | |  | |  | |  | |  | |  | |  | |  | |  | | ● | | ● | | ● | ● | ● |
| Cross-sectional surveys | |  | |  | |  | |  | |  | |  | |  | |  | |  | | ● | |  | | ● | | ● |  | ● |
| **Efficacy Data Collection** ^1^ |  | |  | |  | |  | |  | |  | |  | |  | |  | |  | |  | |  | |  | |  |  |
| Treatment with antimalarial | |  | |  | |  | |  | |  | | ● | |  | |  | |  | |  | |  | |  | |  |  |  |
| Two-weekly field worker visits | |  | |  | |  | |  | |  | |  | |  | |  | | ● | |  | | ● | |  | |  |  |  |
| Monthly field worker visits | |  | |  | |  | |  | |  | |  | |  | |  | |  | |  | |  | | ● | |  |  |  |
| Passive Case Detection^2^ | |  | | ● | | ● | | ● | | ● | |  | | ● | | ● | | ● | | ● | | ● | | ● | | ● | ● | ● |
| Measurement of spleen size (cross-sectional surveys) **(Amended January 17, 2003)** | | ***●*** | | ~~●~~ | |  | |  | |  | |  | |  | |  | |  | |  | |  | |  | | ● |  | ● |
| Recording of bednet use | |  | | ● | |  | |  | |  | |  | |  | |  | |  | |  | |  | |  | | ● |  |  |
| **Laboratory Analyses** |  | |  | |  | |  | |  | |  | |  | |  | |  | |  | |  | |  | |  | |  |  |
| Blood sample: finger prick | | ● | | ● | |  | |  | |  | |  | | ● | |  | | ● | |  | | ● | | ● | | ● |  | ● |
| Blood sample: venous (3 ml) | |  | |  | |  | |  | |  | |  | |  | |  | |  | | ● | |  | |  | |  |  |  |
| Complete blood count | | ● | |  | |  | |  | |  | |  | |  | |  | |  | | ● | |  | |  | |  |  |  |
| Haematocrit | | ● | |  | |  | |  | |  | |  | |  | |  | |  | | ● | |  | |  | | ● |  | ● |
| Creatinine, ALT, bilirubin | | ● | |  | |  | |  | |  | |  | |  | |  | |  | | ● | |  | |  | | ● |  | ● |
| Blood film (parasitaemia) | |  | |  | |  | |  | |  | |  | | ● | |  | | ● | | ● | | ● | | ● | | ● |  | ● |
| HBsAg | |  | | ● | |  | |  | |  | |  | |  | |  | |  | |  | |  | |  | |  |  |  |
| Anti-CS antibodies | |  | | ● | |  | |  | |  | |  | |  | |  | |  | | ● | |  | |  | | ● |  | ● |
| Anti-HBs antibodies | |  | | ● | |  | |  | |  | |  | |  | |  | |  | | ● | |  | |  | | ● |  | ● |
| RF1-like antibodies | | ● | |  | |  | |  | |  | |  | |  | |  | |  | | ● | |  | |  | |  |  |  |
| IFAT | | ● | |  | |  | |  | |  | |  | |  | |  | |  | |  | |  | |  | |  |  |  |
| Parasite genotyping **(Amended January 17, 2003)** | |  | |  | |  | |  | |  | |  | | ● | |  | | ● | | ● | | ● | | ● | | ● |  |  |

● indicates a study procedure that requires documentation in the CRF,  indicates a procedure that does not require documentation

****: Measurement of body weight and height or length, as appropriate for age, at screening (Clinic visit 1) ~~on day 0 (Clinic visit 2~~). Measurement of bodyweight on Month 8½ and Month 21 (Clinic visits 6 and 7)* (Amended August 13, 2002) (Amended January 17, 2003)**

^1^Active detection of infection (ADI) for efficacy data collection: all subjects will be treated with antimalarial two weeks before dose 3 (day 46). Absence of parasitaemia will be determined at clinic visit 4 on day 60. Surveillance for ADI will start on day 74 with the first fieldworker home visit. The cross-sectional survey on day 90 (clinic visit 3) will provide the second occasion for surveillance. Then, if parasitaemia has not been reported at either of these two initial visits, fieldworkers will make visits every two weeks to each subject, starting 6 weeks after dose 3 (day 104) and continuing for 2½ months or until first report of parasitaemia. Fieldworkers will continue surveillance with monthly visits from month 7 to month 8 inclusive or until first report of parasitaemia. If parasitaemia has not been reported previously, surveillance will stop at the cross-sectional survey at month 8½ (clinic visit 6). Finger prick blood samples will be taken at each of the two-weekly or monthly visits for determination of parasitaemia and parasite genotyping.

^2^If a child presenting to Ilha Josina Health Facility reports fever within the preceding 24 hours or has a documented fever (axillary temperature ≥ 37.5°C) then blood will be taken for parasitaemia determination, parasite genotyping and haematocrit.

Double line after month 8½ indicates the end of the surveillance period for ADI (double-blind phase) and Study Conclusion. Thereafter, safety follow-up fieldworker visits at monthly intervals and passive surveillance will continue and one remaining cross-sectional survey will be performed at Month 21 (open phase).

It is the investigator’s responsibility to ensure that the intervals between visits/contacts are strictly followed. These intervals determine a subject’s evaluability in the according to protocol analyses (see Sections 4.5 and 10.5 for details of criteria for evaluability and cohorts to be analyzed).

Intervals between study visits in Active Detection of Infection Cohort

| **Interval for clinic visits** | **Duration of interval** |
| --- | --- |
| 1 (Visit 1 →Visit 2) | Screening ***90*** ~~35~~ to 7 days before first vaccination visit |
| 2 (Visit 2 →Visit 3) | 27-37 days |
| 3 (Visit 3 →Visit 4) | 27-37 days |
| 4 (Visit 4 →Visit 5) | 30-37 days |
| 5 (Visit 5 →Visit 6) | 146-186 days |
| 6 (Visit 5→Visit 7) | 512-568 days |

**(Amended January 17, 2003)**

## Detailed description of study stages/visits for Cohort 2

**When materials are provided by GlaxoSmithKline Biologicals, it is mandatory that all clinical samples (including serum samples) be collected using exclusively those materials in the appropriate manner. The use of other materials could result in the exclusion of the subject from analysis. The investigator must ensure that his/her personnel and the laboratory(ies) under his/her supervision comply with this requirement.**

The parents/guardians will be instructed to contact the investigator immediately should the child manifest any signs or symptoms any time throughout the study period that the parents/guardians perceive as serious.

| **Clinic Visit 1: Screening** | | |
| --- | --- | --- |
| **Days *–90 to* *-7*~~35 to -7~~: Screening (Amended January 17, 2003)** | | |
| · | | Obtain written/thumb-printed informed consent from the parents/guardians. |
| · | | History taking and physical examination - ***baseline measurement of spleen size, body weight and height or length, as appropriate for age* (Amended January 17, 2003)** |
| · | | Checking of inclusion/ exclusion criteria. |
| · | | Finger prick blood sample collected (2 ml, providing a minimum of 1 ml of serum) for baseline analysis of:   - Haematology : Blood Count (BC) which includes platelets, white blood cells, haematocrit and haemoglobin - Biochemistry (creatinine, bilirubin and ALT) - RF1-like antibodies - Indirect fluorescent antibody test (IFAT) for anti-parasite antibodies |
| **Clinic Visit 2: First vaccination (Day 0)** | | |
| **Day 0: Pre vaccination 1** | | |
| · | | Provision of photo ID |
| · | | Checking of inclusion/ exclusion criteria. |
| · | | History-related physical examination – pre-vaccination axillary body temperature, ~~baseline measurement of spleen size, body weight and height or length, as appropriate for age~~ **(Amended August 13, 2002) (Amended January 17, 2003)** |
| · | | Randomization |
| · | | Recording of concomitant medication and bednet use for the previous night. |
| · | | Finger prick blood sample collected (1-2 ml, providing a minimum of 0.5 ml of serum) pre-vaccination for baseline analysis of:   - Serology (anti-HBs and anti-CS antibodies and HBsAg) |
| · | | Intra-muscular (IM) administration of the first vaccine dose in the deltoid region of the left arm. |
| · | | Each vaccinee will be closely observed for one hour or longer if necessary with appropriate medical treatment readily available in case of a rare anaphylactic reaction following the administration of the vaccine. After the observation period, axillary body temperature will be recorded again, and together with any local and general signs and symptoms that may occur, will be recorded in the CRF by the investigator. |
| ***Field worker vaccination follow-up visits 1-3 after first vaccination*** | | |
| ***Days 1, 2 and 3:*** | | |
| · | | Recording of concomitant medication. |
| · | | Reporting of any Serious Adverse Events (SAEs) which the vaccinee has experienced since last visit. |
| · | | Recording of axillary body temperature and any local or general solicited symptoms experienced at time of field worker visit, and within the previous 24 hours, onto diary cards by field workers, on the 3 subsequent days after vaccination. |
| **Clinic Visit 3: Second vaccination (27 to 37 days after Visit 2)** | | |
| **Day 30: Post vaccination 1** | | |
| · | Verification of photo ID and subject number | |
| · | The investigator must record all non-serious adverse events that occurr 1 month (minimum 30 days) post-vaccination 1. | |
| · | Reporting of any Serious Adverse Events (SAEs) which the vaccinee has experienced since the first vaccine dose. | |
| · | Physical examination –pre-vaccination axillary body temperature | |
| · | Checking of elimination criteria and contraindications to vaccination. | |
| · | Recording of concomitant medication. | |
| · | Intra-muscular (IM) administration of the second vaccine dose in the deltoid region of the right arm. | |
| · | Each vaccinee will be closely observed for one hour or longer if necessary with appropriate medical treatment readily available in case of a rare anaphylactic reaction following the administration of the vaccine. After the observation period, axillary body temperature will be retaken, and together with local and general signs and symptoms that may occur, will be recorded in the CRF by the investigator. | |
| ***Field worker vaccination follow-up visits 4-6 after second vaccination*** | | |
| ***Days 31, 32 and 33:*** | | |
| · | Recording of concomitant medication. | |
| · | | Reporting of any Serious Adverse Events (SAEs) which the vaccinee has experienced since last visit. |
| · | | Recording of axillary body temperature and any local or general solicited symptoms experienced at time of field worker visit, and within the previous 24 hours, onto diary cards by field workers, on the 3 subsequent days after vaccination. |
| ***Field worker visit 7: for Active Detection of Infection (14 days pre dose 3)*** | | |
| ***Day 46:*** | | |
| · | Treatment of all subjects with antimalarial (administration of first dose of amodiaquine and SP and provision of following two doses) without checking for parasitaemia | |
| · | | Reporting of any Serious Adverse Events (SAEs) which the vaccinee has experienced since last visit. |
| **Clinic Visit 4: Third vaccination (27 to 37 days after Visit 3)** | | |
| **Day 60: Post vaccination 2** | | |
| · | Verification of photo ID and subject number | |
| · | The investigator must record all non-serious adverse events that occur 1 month (minimum 30 days) post-vaccination 2. | |
| · | Reporting of any Serious Adverse Events (SAEs) which the vaccinee has experienced since the previous vaccine dose. | |
| · | Physical examination –pre-vaccination axillary body temperature . | |
| · | Checking of elimination criteria and contraindications to vaccination. | |
| · | Recording of concomitant medication. | |
| · | Finger prick blood sample collected (0.5-1 ml) for:   - Blood film for parasitaemia determination - ***Parasite genotyping***   **(Amended January 17, 2003)** | |
| · | Intra-muscular (IM) administration of the third vaccine dose in the deltoid region of the left arm. | |
| · | Each vaccinee will be closely observed for one hour or longer if necessary with appropriate medical treatment readily available in case of a rare anaphylactic reaction following the administration of the vaccine. After the observation period, axillary body temperature will be retaken, and together with local and general signs and symptoms that may occur, will be recorded in the CRF by the investigator. | |
| ***Field worker vaccination follow-up visits 7-9 after third vaccination*** | | |
| ***Days 61, 62 and 63:*** | | |
| · | Recording of concomitant medication. | |
| · | | Reporting of any Serious Adverse Events (SAEs) which the vaccinee has experienced since last visit. |
| · | | Recording of axillary body temperature and any local or general solicited symptoms experienced at time of field worker visit, and within the previous 24 hours, onto diary cards by field workers, on the 3 subsequent days after vaccination. |
| ***Field worker visit 10 for Active Detection of Infection (14 days post dose 3)*** | | |
| ***Day 74:*** | | |
| · | Recording of axillary body temperature: if temperature ≥ 37.5°C or history of fever, the child will be brought to Ilha Josina Health Facility | |
| · | Surveillance for infection morbidity questionnaire for Cohort 2 completed | |
| · | Finger prick blood sample collected (0.5-1 ml) for:   - Blood film for parasitaemia determination - Parasite genotypingby PCR | |
| · | | Reporting of any Serious Adverse Events (SAEs) which the vaccinee has experienced since last visit. |
| **Clinic Visit 5: Follow-up visit after third vaccination and Cross-sectional Survey (30 to 37 days after Visit 4)** | | |
| **Day 90: Post vaccination 3** | | |
| · | Verification of photo ID and subject number | |
| · | Checking of elimination criteria. | |
| · | Recording of concomitant medication. | |
| · | | Physical examination |
| · | The investigator must record all non-serious adverse events that will have occurred 1 month (minimum 30 days) post-vaccination 3. | |
| · | Reporting of any Serious Adverse Events (SAEs) which the vaccinee has experienced since the previous vaccine dose. | |
| · | | Venous blood sample collected (3 ml, providing a minimum of 1 ml of serum) for analysis of:   - Serology (anti-HBs, RF1-like and anti-CS antibodies) - Haematology : Blood Count (BC) which includes platelets, white blood cells, haematocrit and haemoglobin - Biochemistry (creatinine, bilirubin and ALT) - Blood film for parasitaemia determination - Parasite genotyping by PCR |
| ***Field worker visits 12-17 for Active Detection of Infection (visits every two weeks)*** | | |
| ***Days 104, 118, 132, 146, 160 and 174:*** | | |
| · | Recording of axillary body temperature: if temperature ≥ 37.5°C or history of fever, the child will be brought to Ilha Josina Health Facility | |
| · | Surveillance for infection morbidity questionnaire for Cohort 2 completed | |
| · | Finger prick blood sample collected (0.5-1ml) for analysis of:   - Blood film for parasitaemia determination - Parasite genotypingby PCR | |
| · | | Reporting of any Serious Adverse Events (SAEs) which the vaccinee has experienced since last visit. |
| ***Field worker visits 18-19 for Active Detection of Infection  (monthly visits)*** | | |
| ***Months 7 and 8:*** | | |
| · | Recording of axillary body temperature: if temperature ≥ 37.5°C or history of fever, the child will be brought to Ilha Josina Health Facility) | |
| · | Surveillance for infection morbidity questionnaire for Cohort 2 completed | |
| · | Finger prick blood sample collected (0.5-1 ml) for analysis of:   - Parasite genotyping by PCR - Blood film for parasitaemia determination | |
| · | | Reporting of any Serious Adverse Events (SAEs) which the vaccinee has experienced since last visit. |
| **Clinic Visit 6: Cross-sectional Survey at Month** 8½ **(146 to 186 days after Visit 5)** | | |
| **Month** 8½**:** | | |
| · | Verification of photo ID and subject number | |
| · | Checking of elimination criteria. | |
| · | Reporting of any Serious Adverse Events (SAEs) which the vaccinee has experienced since the last visit. | |
| · | Recording of concomitant medication. | |
| · | | Recording of bednet use for the previous night. |
| · | Recording of axillary body temperature. | |
| · | | Evaluation of spleen size (classification) and body weight |
| · | | Finger prick blood sample collected (1-2 ml, providing a minimum of 0.5 ml of serum) for analysis of:   - Serology (anti-HBs and anti-CS antibodies) - Haematology : haematocrit - Biochemistry (creatinine, bilirubin and ALT) - Blood film for parasitaemia determination - Parasite genotyping by PCR |
| · | | End of surveillance period for Cohort 2 (double-blind phase) and Study Conclusion |
| ***Field worker monthly follow-up visits 20-32*** | | |
| ***Months 9 to 21:*** | | |
| · | Reporting of any Serious Adverse Events (SAEs) which the vaccinee has experienced last visit. | |
| · | Recording of concomitant medication. | |
| **Clinic Visit 7: Cross-sectional Survey at Month 21 (512 to 568 days after Visit 5)** | | |
| **Month 21:** | | |
| · | Verification of photo ID and subject number | |
| · | Checking of elimination criteria. | |
| · | Reporting of any Serious Adverse Events (SAEs) which the vaccinee has experienced since the last visit. | |
| · | Recording of concomitant medication. | |
| · | Recording of axillary body temperature. | |
| · | | Evaluation of spleen size (classification) and body weight |
| · | | Finger prick blood sample collected (1-2 ml, providing a minimum of 0.5 ml of serum) for analysis of:   - Serology (anti-HBs and anti-CS antibodies) - Haematology : haematocrit - Biochemistry (creatinine, bilirubin and ALT) - Blood film for parasitaemia determination |
| · | | End of follow-up period for safety surveillance and Study Conclusion of single-blind surveillance phase . |

## Clinical examination at cross-sectional surveys

All subjects will be examined at the cross-sectional surveys at months 8½ and 21 to determine their body weights, axillary temperatures and spleen sizes, according to Hackett’s method[[31]](#endnote-32) (see Table 3)

Table 3: Classification of spleen sizes according to Hackett

| Class | Description |
| --- | --- |
| 0 | Normal spleen not palpable even on deep inspiration. |
| 1 | Spleen palpable below the costal margin, usually on deep inspiration. |
| 2 | Spleen palpable below the costal margin, but not projected beyond a horizontal line half way between the costal margin and the umbilicus, measured along a line dropped vertically down from the left nipple. |
| 3 | Spleen with lowest palpable point projected more than half way to the umbilicus but not below a line drawn horizontally through it. |
| 4 | Spleen with lowest palpable point projected below the umbilical level but not projected beyond a horizontal line situated half way between the umbilicus and the symphysis pubis. |
| 5 | Spleen with lowest palpable point beyond the lower limit of class 4. |

## Sample handling and analysis

### Collection of blood samples

Due to the mistrust of the local population to blood sampling by venipuncture, finger prick samples will be obtained whenever possible. Finger pricking will be repeated up to a maximum of three times for each sampling time point (see SOPs at investigator’s site).

### Treatment and storage of biological samples

See Appendix D of the protocol for details of treatment and storage of biological samples.

See Appendix E for instructions for shipment of biological samples.

### Laboratory assays

Additional tests may be performed if deemed necessary by the investigator or GSK Biologicals for the further investigation of the toxicity/immunogenicity of the vaccine. Detailed SOPs governing the assays at the CISM laboratory will be maintained by the Principal Investigator and respective staff team members who will observe the specific SOP requirements. Details of the serological assays can be found in Appendix F.

#### Laboratory testing for Cohort 1

***Laboratory tests at screening: clinic visit 1***

1-2 ml of blood will be obtained from finger prick samples to provide whole blood and a minimum of 0.5 ml of serum for:

- haematology (blood count [BC], which includes platelets, white blood cells, haematocrit and haemoglobin)
- biochemistry (creatinine, ALT, bilirubin)
- indirect fluorescent antibody test for anti-parasite antibodies

***Laboratory tests during the study: clinic visit 2 (day 0)***

1-2 ml of blood will be obtained from finger prick samples to provide whole blood and a minimum of 0.5 ml of serum for:

- serology (HBsAg and anti-CS antibodies)

***Laboratory tests during the study: clinic visit 5 (day 90)***

1-2 ml of blood will be obtained from finger prick samples to provide whole blood and a minimum of 0.5 ml of serum for:

- haematology (blood count [BC], which includes platelets, white blood cells, haematocrit and haemoglobin)
- biochemistry (creatinine, ALT, bilirubin)
- serology (anti-CS antibodies)

***Laboratory tests during the study: clinic visits 6 (month*** 8½***, day 256) and 7 (month 21, day 630)***

1-2 ml of blood will be obtained from finger prick samples to provide whole blood and a minimum of 0.5 ml of serum for:

- haematology (haematocrit)
- biochemistry (creatinine, ALT, bilirubin)
- serology (anti-CS antibodies)
- determination of parasitaemia and parasite density
- ***parasite genotyping by PCR***

**(Amended August 13, 2002)**

#### Laboratory testing for Cohort 2

***Laboratory tests at screening: clinic visit 1***

2 ml of blood will be obtained from finger prick samples to provide a minimum of 1 ml of serum for:

- haematology (blood count [BC], which includes platelets, white blood cells, haematocrit and haemoglobin)
- biochemistry (creatinine, ALT, bilirubin)
- RF1-like antibodies
- indirect fluorescent antibody test for anti-parasite antibodies

***Laboratory tests during the study: clinic visit 2 (day 0)***

1-2 ml of blood will be obtained from finger prick samples to provide a minimum of 0.5 ml of serum for:

- serology (anti-CS and anti-HBs antibodies and HBsAg)

***Laboratory tests during the study: clinic visit 4 (day 60)***

0.5-1 ml of blood will be obtained from finger prick samples to provide whole blood for:

- determination of parasitaemia and parasite density
- ***parasite genotyping by PCR***

**(Amended January 17, 2003)**

***Laboratory tests during the study: field visits 11-19 (days 74, 104, 118, 132, 146, 160, 174 and months 7 and 8)***

0.5-1 ml of blood will be obtained from finger prick samples to provide whole blood for:

- determination of parasitaemia and parasite density
- parasite genotyping by PCR

***Laboratory tests during the study: clinic visit 5 (day 90)***

3 ml of venous blood will be obtained to provide whole blood and a minimum of 1 ml of serum for:

- haematology (blood count [BC], which includes platelets, white blood cells, haematocrit and haemoglobin)
- biochemistry (creatinine, ALT, bilirubin)
- serology (anti-HBs, RF1-like and anti-CS antibodies)
- determination of parasitaemia and parasite density
- parasite genotyping by PCR

***Laboratory tests during the study: clinic visit 6 (month 8½, day* *256*)**

1-2 ml of blood will be obtained from finger prick samples to provide whole blood and a minimum of 0.5 ml of serum for:

- haematology (haematocrit)
- biochemistry (creatinine, ALT, bilirubin)
- serology (anti-CS and anti-HBs antibodies)
- determination of parasitaemia and parasite density
- parasite genotyping by PCR

***Laboratory tests during the study: clinic visit 7 (month 21, day 630)***

1-2 ml of blood will be obtained from finger prick samples to provide whole blood and a minimum of 0.5 ml of serum for:

- haematology (haematocrit)
- biochemistry (creatinine, ALT, bilirubin)
- serology (anti-CS and anti-HBs antibodies)
- determination of parasitaemia and parasite density

Separation of serum from the finger prick or venous blood samples will be performed at the investigator’s centre.

Determination of parasitaemia and parasite density, haematology and biochemistry will be performed at the CISM laboratories. The acceptable reference ranges will be specified in an SOP at the investigator site and in the GSK central study file.

For anti-CS (anti-R32LR) antibody determination, serum samples (minimum 100 µl) will be sent to GSK Biologicals. Aliquots will be sent to Walter Reed Army Institute of Research (WRAIR) USA, for anti-CS antibody testing.

For HBsAg testing, 100 µl serum samples will be sent to GSK Biologicals. Aliquots will be sent to the Microbiology Service at Hospital Clinic, Barcelona, Spain.

For anti-HBs antibody determination, serum samples (minimum 400 µl) will be sent to GSK Biologicals.

For RF1-like antibodies, serum samples (minimum 250 µl) will be sent to GSK Biologicals.

Indirect fluorescent antibody test for anti-parasite antibodies will be performed at CISM according to previously published methods[[32]](#endnote-33),[[33]](#endnote-34). The anti-parasite antibody levels at screening will be assessed to determine the comparability between study areas in terms of malaria transmission intensity.

For falciparum parasite genotyping by polymerase chain reaction, whole blood samples are collected on filter paper and stored at the investigator’s site until analysis.

***Summary of laboratory assays to be performed***

| **Assay** | **Marker** | **Assay method** | **Test Kit/ Manufacturer** | **Assay unit** | **Assay cut-off** | **Laboratory*** |
| --- | --- | --- | --- | --- | --- | --- |
| Anti-CS antibodies | R32LR | ELISA | In-house ELISA | μg/ml | 1 μg/ml | WRAIR, US |
| Anti-HBs antibodies |  | EIA | AUSAB EIA  ABBOTT^**^ | mIU/ml | 10 mIU/ml | GSK Bio*, Rixensart |
| RF1-like antibodies |  | Competitive ELISA | In house ELISA | EL.U/ml | TBD | GSK Bio, Rixensart |
| Anti-parasite antibodies |  | IFAT | In-house  IFAT | Titre dilution | TBD | CISM |
| Parasite genotyping |  | PCR | In house PCR | N/A |  |  |
| HBsAg |  | ELISA | ETI-MAK-4  DIASORIN^®**^ | Qualitative | +/- | Hospital Clinic, Barcelona ^*^ |
| * or designated validated laboratory. ** or equivalent  ELISA: Enzyme-Linked Immunosorbent Assay EIA: Enzyme immunoassay. TBD: to be determined. IFAT: Indirect Fluorescent Antibody Test. PCR: polymerase chain reaction. | | | | | | |

### Serology plan

Serological responses in Cohort 1 will be primarily measured by evaluating antibody responses to CS (anti-CS also referred to as R32LR antibodies). Antibody levels will be measured by standard ELISA methodologies using plate adsorbed R32LR antigen with a standard reference antibody as a control. Results will be reported in μg/ml. Anti-HBs antibody levels will be measured in samples from Cohort 2 only by ELISA with a commercial AUSAB EIA kit from Abbott or equivalent. In addition, in Cohort 2, RF1-like antibody levels will be measured in all subjects as a qualitative measurement of an immune response to the HBsAg using an in-house competitive ELISA. HBsAg levels will be measured in both cohorts by ELISA using the ETI-MAK-4 commercial kit from Diasorin®.

Serum for antibody determination will be collected by phlebotomy or finger prick (as specified at each visit in sections 5.4 and 5.6). Samples for safety will be analysed at the time they are collected.

Serum samples (minimum of 0.5 ml for finger prick samples and 1 ml for venous samples) from each child will be shipped to GSK Biologicals, Rixensart, Belgium. Serology testing at screening is different in both cohorts. Therefore, a list of enrolled subjects for each cohort will also be sent to GSK Biologicals to enable the identification of samples into cohorts where necessary.

HBsAg will be measured by the Microbiology Service of Hospital Clinic in Barcelona, according to the manufacturer’s instructions and laboratory SOPs.

Any serum not immediately used in antibody assays will be stored at -20°C or less and may be used in additional *in vitro* tests which may be useful in evaluating the immune response to the test vaccine preparation in these subjects, or to evaluate potential toxicity of the vaccine. Assay details are provided in Appendix F.

Blood sampling timepoints of Cohort 1

| **Blood sampling timepoint** | | | **Marker** | **No. subjects** | **Laboratory** |
| --- | --- | --- | --- | --- | --- |
| **Timing** | **Timepoint** | **Clinic Visit no.** |  |  |  |
| Pre | Day 0 | 2 | - HBsAg | 1564 | Hospital Clinic, Barcelona |
|  |  |  | - Anti-CS | 1564 | WRAIR, US |
| Post III | Day 90 | 5 | - Anti-CS | 1564 | WRAIR, US |
| Post III | Month 9 | 6 | - Anti-CS | 1564 | WRAIR, US |
| Post III | Month 21 | 7 | - Anti-CS | 1564 | WRAIR, US |

Blood sampling timepoints of Cohort 2

| **Blood sampling timepoint** | | | **Marker** | **No. subjects** | **Laboratory** |
| --- | --- | --- | --- | --- | --- |
| **Timing** | **Timepoint** | **Clinic Visit no.** |  |  |  |
| Screening | ***-90***-~~35~~ to ‑7 | 1 | - RF1-like antibodies | ***416*** ~~356~~ | GSK Bio |
| Pre | Day 0 | 2 | - HBsAg | ***416*** ~~356~~ | Hospital Clinic, Barcelona |
|  |  |  | - Anti-CS |  | WRAIR, US |
|  |  |  | - Anti-HBs |  | GSK Bio |
| Post III | Day 90 | 5 | - Anti-CS | ***416*** ~~356~~ | WRAIR, US |
|  |  |  | - RF1-like antibodies |  | GSK Bio |
|  |  |  | - Anti-HBs |  | GSK Bio |
| Post III | Month 9 | 6 | - Anti-CS | ***416*** ~~356~~ | WRAIR, US |
|  |  |  | - Anti-HBs |  | GSK Bio |
| Post III | Month 21 | 7 | - Anti-CS | ***416*** ~~356~~ | WRAIR, US |
|  |  |  | - Anti-HBs |  | GSK Bio |

**(Amended January 17, 2003)**

In case of insufficient blood sample volume to perform serology assays in Cohort 2, the samples will be analyzed with first priority given to HBsAg testing (where applicable) then anti-HBs testing RF1-like antibody testing and finally anti-CS testing.

# Study Vaccines and Administration

## Study vaccines

The candidate vaccine to be used has been developed and manufactured by GlaxoSmithKline Biologicals.

The Quality Control Standards and Requirements for the candidate vaccine are described in separate release protocols and the required approvals have been obtained.

Commercial vaccines (Engerix-B™, Prevnar® and Hiberix™) are assumed to comply with the specifications given in the manufacturer's Summary of Product Characteristics.

See Appendix G for details of vaccine supplies, packaging and vaccine accountability.

### RTS,S/AS02A malaria candidate vaccine

The malaria vaccine used in the present study contains:

RTS,S antigen:

- The lyophilized antigen pellet contains 50 μg of RTS,S per 0.5 mL of reconstituted vaccine. 12.6 mg of sucrose is used as a cryopreservative. The antigen is provided in 3 ml vials.

AS02A adjuvant:

- This adjuvant contains 50 μg of 3-deacylated monophosphoryl lipid A (3D-MPL), 50 μg of QS21 (QS21 is a triterpene glycoside purified from the bark of Quillaja Saponaria) and 250 µl of an oil/water emulsion (SB62) per 0.5 ml dose in phosphate buffered saline (PBS). The adjuvant is provided in prefilled syringes.

The presentation of the reconstituted RTS,S/AS02A malaria vaccine is an opaque milky liquid.

### Hepatitis B vaccine (Engerix-B™)

Hepatitis B vaccine (Engerix-B™) is the control vaccine to be given to children of ***24*** ~~18~~ months old or more. The paediatric dosage vial contains 10 µg of purified major hepatitis B surface antigen. It is prepared as a suspension of 0.5 ml volume per vial. The content, upon storage may present a fine white deposit with a clear colourless supernatant. Once shaken the vaccine is slightly opaque.
**(Amended January 17, 2003)**

### *Haemophilus influenzae* type b vaccine (Hiberix™)

*Haemophilus influenzae* type b vaccine (Hiberix™) is the control vaccine to be given to children under ***24*** ~~18~~ months of age at the second dose. Each single dose of vaccine contains:
**(Amended January 17, 2003)**

- 10 µg of purified polyribosyl-ribitol-phosphate (PRP) capsular polysaccharide of *Haemophilus influenzae* type b
- 20-40 µg of tetanus toxoid
- 12.6 mg of lactose.

It is presented as a lyophilised white pellet in a vial with a diluent (0.5 ml of sterile saline for injection), a clear colourless liquid, in a prefilled syringe.

### 7-valent pneumococcal conjugate vaccine (Prevnar®)

The 7-valent pneumococcal conjugate vaccine (Prevnar®) is the control vaccine to be given to children under ***24*** ~~18~~ months of age at the first and third doses. Prevar® is licensed by Wyeth Lederle Vaccines. Each single dose of vaccine contains in a volume of 0.5 ml:
**(Amended January 17, 2003)**

- Mixture of 7 pneumococcal CRM_197_ conjugate serotypes 4, 6B, 9V, 14, 18C, 19F, 23F. Each dose contains 2 µg of saccharide of serotypes 4, 9V, 14, 18C, 19F and 23F, and 4 μg of serotype 6B.
- 0.125 mg of aluminium as aluminium phosphate.

The presentation of Prevnar® is a whitish liquid suspension (0.5 ml) in a monodose vial.

## Dosage and administration

*Reconstitution of the malaria vaccine with diluent and administration:*

RTS,S/AS02A will be supplied such that the reconstituted vaccine volume will be 0.5 ml per vial. One paediatric dose (0.25 ml dose volume) will be aspirated from each vial and used.

Disinfect top of vaccine vial (pellet) with alcohol swabs and let dry. Inject complete contents of one PFS of diluent into vial of lyophilised vaccine. Remove and discard the syringe and needle under appropriate safety precautions. The pellet is then dissolved by gently shaking the vial. Wait for 1 minute to ensure complete dissolution of vial contents before withdrawing 0.25 ml (volume required for RTS,S/AS02A paediatric dose) of the reconstituted vaccine solution using a fresh needle and syringe for injection. The reconstituted vaccine should be administered by slow IM injection, using ***a 25G needle with length of 1 inch (25 mm),*** in the deltoid muscle within 4 hours of reconstitution (storage at 2°C-8°C). **(Amended August 13, 2002)**

*Preparation of the hepatitis B vaccine and administration:*

Disinfect the top of the vial with alcohol swabs and shake gently while letting the alcohol dry. Then withdraw the contents of the vial into a syringe for injection. The vaccine should be administered soon afterwards intramuscularly in the deltoid region, ***using a 25G needle with length of 1 inch (25 mm)***. **(Amended August 13, 2002)**

*Reconstitution of the* *Haemophilus influenzae type b vaccine (Hiberix™) with diluent and administration:*

Disinfect top of vaccine vial (pellet) with alcohol swabs and let dry. Inject complete contents of one PFS of diluent into vial of lyophilised vaccine. Remove and discard the syringe and needle under appropriate safety precautions. The pellet is then completely dissolved by shaking the vial. Wait for one minute to ensure complete dissolution of vial contents before withdrawing into a new syringe for injection. The reconstituted vaccine should be administered soon afterwards by slow IM injection in the deltoid muscle, ***using a 25G needle with length of 1 inch (25 mm)***. **(Amended August 13, 2002)**.

All vaccinees will be observed closely for at least one hour with appropriate medical treatment readily available in case of a rare anaphylactic reaction following the administration of vaccines.

*Preparation of the 7-valent pneumococcal conjugate vaccine (Prevnar®) and administration:*

Shake gently before use to obtain a homogeneous whitish suspension. Disinfect the top of the vial with alcohol swabs and then withdraw the contents of the vial (0.5 ml) into a syringe for injection. The vaccine should be administered soon afterwards intramuscularly in the deltoid region, ***using a 25G needle with length of 1 inch (25 mm)***. **(Amended August 13, 2002)**.

## Storage

ALL VACCINE VIALS (MALARIA, HAEMOPHILUS INFLUENZAE TYPE B [HIBERIX™], 7-VALENT PNEUMOCOCCAL CONJUGATE VACCINE [PREVNAR®] AND HEPATITIS B [ENGERIX-B™]), THE AS02A AND HIBERIX™ DILUENTS MUST BE STORED IN THE REFRIGERATOR (+2°C to +8°C) AND MUST NOT BE FROZEN

All vaccines must be stored in a safe and locked place with no access for unauthorised personnel. Storage temperature should be monitored daily, according to SOPs at the investigator’s site. It is advisable to have an alarm system and a back-up refrigerator/ freezer in case of power failure/ breakdown.

## Treatment allocation and randomization

Study subjects will not be randomly assigned to Cohorts 1 and 2 instead the required number of subjects from Manhiça town will enrolled for Cohort 1 and from Ilha Josina area for Cohort 2.

A randomization list will be created at GSK Biologicals to allocate subjects between the two groups (malaria vaccine group or control vaccine group) by block randomization. Vaccines (RTS,S/AS02A, Engerix-B™, Prevnar® and Hiberix™) will be numbered accordingly and dispensed to the site in individual boxes labeled with unique study numbers.

Study numbers ***0101‑0700 ~~400~~*** will be provided for the children less than ***24*** ~~18~~ months of age and study numbers ***0701‑2300 ~~0401‑2200~~*** will be provided for those children older than ***24*** ~~18~~ months of age. On the day of first vaccination, children will be allocated according to their age, sequentially in order of their arrival, to the next study number. For children of less than ***24*** ~~18~~ months, the numbering will start at ***0101*** and for children of ***24*** ~~18~~ months and over, the numbering will start at ***0701 ~~0401~~***. A total of ***1980*** ~~1920~~ children will be enrolled. When this number is reached, enrollment will cease. Because ***495*** ~~240~~ children are predicted to be less than ***24*** ~~18~~ months of age but this number is not known precisely until after screening results are available, over-randomization is necessary and ***2200*** ~~2100~~ numbers will be prepared for ***1980*** ~~1920~~ children. Thus, over-randomization will allow some flexibility at the time of enrolment.
**(Amended August 13, 2002) (Amended January 17, 2003)**

## Method of blinding and breaking the study blind

The malaria vaccine RTS,S/AS02A and control vaccines (Engerix-B ™, Prevnar® or Hiberix™) are different in physical appearance. The dissimilar packaging (vials or vials and prefilled syringes) and distinctive labelling of the vaccines require specific steps to ensure that vaccines are administered and evaluated in a double-blinded manner.

'Double-blinded' means that for the vaccination phase of the study (see section 6.5.1) the vaccine recipient, their parents and those responsible for the evaluation of safety, efficacy and immunogenicity study parameters, will all be unaware which vaccine preparation was administered to a particular subject.

'Single-blinded' means that the vaccine recipient and their parents will be unaware of the treatment allocation. In addition, staff responsible for endpoint evaluation at the site, working in the health facilities and taking part in the cross-sectional surveys will not be unblinded.

Specific tasks of this study will be carried out by separate teams with specified functions. To ensure blinding, an individual may serve on only one team.

Before shipping the test vaccine (RTS,S/AS02A), the 7-valent pneumococcal conjugate vaccine (Prevnar®), *Haemophilus influenzae* type b vaccine (Hiberix™) and hepatitis B vaccine (Engerix-B™) to Mozambique , GSK Biologicals will be responsible for ensuring that individual test and control vaccine doses are packaged in identical generic boxes which are then labelled with subject study numbers (SSNs). GSK Biologicals will generate and hold the computer code that associates each study number with a specific vaccine. The code will not be made available to anyone on the team working on the project within GSK, during the study.

### Breaking of the study blind

Code break envelopes, for each study subject and associating each study number with a specific vaccine, will be kept in a safe and locked place with no access for unauthorised personnel by the Local Safety Monitor in Mozambique as well as by Central Safety at GlaxoSmithKline Biologicals, Rixensart.

If deemed necessary for reasons such as safety, the Local Safety Monitor in Mozambique as well as GSK Biologicals Central Safety will unblind the specific subject without revealing the study blind to the investigators.

A formal reporting and analysis plan (RAP) will be developed jointly by CISM investigators, MVI and GSK and submitted to the DSMB for consultation prior to breaking the study blind. Once the double-blind primary phase of the study is completed and the GSK Biologicals reference database locked, GlaxoSmithKline Biologicals will be responsible for initiating the execution of the analytic plan in collaboration with CISM and breaking the blind.

## Replacement of unusable vaccine doses

In addition to study vaccines, additional malaria and control vaccines will be provided to replace unusable doses (see Appendix G for details of supplies). This additional supply of vaccines will be labeled with code letters (A, B, C, D, E and F). Each letter will designate either Engerix-B™, Hiberix™, Prevnar® or RTS,S/AS02A. The investigator will receive three sets of sealed envelopes for subjects ***0101 to 0700*** ~~0001 to 0300~~, each set corresponding to each dose of vaccine (doses at months 0, 1, 2), and one set of envelopes for subjects ***0701 to 2300*** ~~0301 to 2100~~. If a vaccine dose needs replacement, the envelope with the corresponding subject number will designate the replacement without unblinding the study.
**(Amended January 17, 2003)**

## Packaging

See Appendix G.

## Vaccine accountability

See Appendix G.

## Concomitant medication/treatment

Any immunosuppressants or other immune-modifying drugs or treatments, any vaccine other than the study vaccine(s) and any antipyretics administered at ANY time during the period starting 30 days prior to the first dose of study vaccine(s) and ending one month (minimum 30 days) after the last dose of study vaccine(s) must be recorded in the CRF with trade name and/or generic name of the medication, medical indication, total daily dose, route of administration, start and end dates of treatment. No follow-up will be done for concomitant medication unless it is related to an SAE or a solicited/unsolicited symptom ongoing at the end of the 30 days after the last vaccine dose.

Any other concomitant medication administered prophylactically in anticipation of reaction to the vaccination must also be recorded in the CRF with trade name and/or generic name of the medication, total daily dose, route of administration, start and end dates of treatment and coded as ‘Prophylactic’. If associated with an adverse experience, the adverse experience code or number should be indicated.

During the efficacy surveillance periods for both cohorts(day 90 to month 21 for Cohort 1 and day 90 to month 9 for Cohort 2), all antimalarial drug usage will be documented to determine the period at risk. All prescribed medication will be captured by the morbidity surveillance system. In addition, non-prescribed medication will be solicited at the monthly field worker visits. All additional usage of antimalarial will be documented on the subject’s CRF.

# Health Economics

Not applicable.

# Adverse Events

The recording of adverse events is an important aspect of study documentation. It is the responsibility of the investigator to document all adverse events according to the detailed guidelines set out below.

The subjects’ parents/guardians will be instructed to contact the investigator immediately should the subject manifest any signs or symptoms they perceive as serious.

## Eliciting and documenting adverse events

***Adverse event definition***

An adverse event includes any noxious, pathological or unintended change in anatomical, physiological or metabolic functions as indicated by physical signs, symptoms and/or laboratory detected changes occurring in any phase of the clinical study whether associated with the study vaccine, active comparator or placebo and whether or not considered vaccination related. This includes an exacerbation of pre-existing conditions or events, intercurrent illnesses, or vaccine or drug interaction. Anticipated day-to-day fluctuations of pre-existing conditions that do not represent a clinically significant exacerbation need not be considered adverse events. Discrete episodes of chronic conditions occurring during a study period should be reported as adverse events in order to assess changes in frequency or severity.

Adverse events should be documented in terms of a medical diagnosis(es). When this is not possible, the adverse event should be documented in terms of signs and/or symptoms observed by the investigator or reported by the subject at each study visit.

Once documented, all unsolicited adverse events will be classified according to the Medical Dictionary for Regulatory Activities (MedDRA) to enable analysis of the data.

Pre-existing conditions or signs and/or symptoms (including any which are not recognized at study entry but are recognized during the study period) present in a subject prior to the start of the study should be recorded on the Medical History form within the subject's CRF.

Adverse events which occur after informed consent is obtained, but prior to vaccination, will be documented on the Medical History form within the subject's CRF as instructed by the GlaxoSmithKline Biologicals’ Study Monitor.

Although not considered as an adverse event, hospitalization for either elective surgery related to a pre-existing condition which did not increase in severity or frequency following initiation of the study, or for routine clinical procedures (including hospitalization for “social” reasons) that are not the result of an adverse event, must be recorded in the CRF. If the hospitalization arises from a pre-existing condition, or was planned prior to the first vaccination, it should be recorded in the Medical History form of the CRF. If it was planned after the first vaccination, it should be recorded in the adverse event page of the CRF. In both cases, it should be recorded as ‘Hospitalization (Not an adverse event)’, and the relationship to vaccination will be checked “No”.

N.B. Adverse events to be recorded as endpoints are described in Section 8.1.1. All other adverse events will be recorded as unsolicited adverse events.

***Surveillance period for occurrence of adverse events***

All adverse events occurring within one month (minimum 30 days) following administration of each dose of vaccine/comparator must be recorded on the Adverse Event form in the subject's CRF, irrespective of severity or whether or not they are considered vaccination-related.

Additionally, all serious adverse events (see Section 8.5) occurring during the period starting from the day of administration of the first dose of study vaccine/comparator to each subject and ending within 19 months following administration of the last dose of study vaccine/comparator for that subject must be recorded. See Section 8.5.2 for instructions for reporting and recording of serious adverse events.

Instances of death, cancer or congenital abnormality in offspring if brought to the attention of the investigator AT ANY TIME after cessation of study AND suspected by the investigator to be related to study vaccine, should be reported to the Study Contact for Reporting Serious Adverse Events (see Section 8.5.2).

***Recording adverse events***

At each visit/assessment, all adverse events either observed by the investigator or one of his clinical collaborators or reported by the subject’s parent/guardian spontaneously or in response to a direct question will be evaluated by the investigator. Adverse events not previously documented in the study will be recorded in the Adverse Event form within the subject's CRF. The nature of each event, date and time (where appropriate) of onset, outcome, intensity and relationship to vaccination should be established. Details of any corrective treatment should be recorded on the appropriate page of the CRF. See Section 8.5.2 for instructions for reporting and recording of serious adverse events.

As a consistent method of soliciting adverse events, the subject or their legal representative should be asked a non-leading question such as:

"Has your child acted differently or felt different in any way since receiving the vaccine or since the last visit?"

N.B. The investigator should record only those adverse events having occurred within the time frames defined above.

Adverse events already documented in the CRF, i.e. at a previous assessment, and designated as ‘ongoing’ should be reviewed at subsequent visits, as necessary. If these have resolved, the documentation in the CRF should be completed. N.B. If an adverse event changes in frequency or intensity during a study period, a new record of the event will be started.

### Solicited adverse events

**Local (injection site) adverse events**

- Pain at injection site
- Swelling at injection site

**General adverse events**

- Fever (defined as axillary temperature ≥ 37.5°C)
- Drowsiness
- Loss of appetite
- Irritability / fussiness

## Assessment of intensity

Intensity of the following adverse events should be assessed as described:

| **Adverse Event** | **Intensity grade** | | **Parameter** | |
| --- | --- | --- | --- | --- |
| Pain at injection site | 0 | | Absent | |
|  | 1 | | Minor reaction to touch | |
|  | 2 | | Cries/protests on touch | |
|  | 3 | | Cries when limb is moved/spontaneously painful | |
| Swelling at injection site | |  | Record greatest surface diameter in mm | |
| Fever* | |  | | Record temperature in °C |
| Irritability/Fussiness | 0 | | Behavior as usual | |
|  | 1 | | Crying more than usual/ no effect on normal activity | |
|  | 2 | | Crying more than usual/ interferes with normal activity | |
|  | 3 | | Crying that cannot be comforted/ prevents normal activity | |
| Drowsiness | 0 | | Behavior as usual | |
|  | 1 | | Drowsiness easily tolerated | |
|  | 2 | | Drowsiness that interferes with normal activity | |
|  | 3 | | Drowsiness that prevents normal activity | |
| Loss of appetite | 0 | | Normal | |
|  | 1 | | Eating less than usual/ no effect on normal activity | |
|  | 2 | | Eating less than usual/ interferes with normal activity | |
|  | 3 | | Not eating at all | |
| *Fever is defined as axillary temperature ≥ 37.5°C. | | | | |

The maximum intensity of local injection site swelling will be scored at GSK Biologicals as follows:

| 0 | : | none |
| --- | --- | --- |
| 1 | : | < 5 mm |
| 2 | : | 5 - 20 mm |
| 3 | : | > 20 mm |

The maximum intensity of fever will be scored at GSK Biologicals as follows:

| 0 | : | < 37.5°C |
| --- | --- | --- |
| 1 | : | 37.5 – 38.0°C |
| 2 | : | > 38 – 39.0°C |
| 3 | : | > 39.0°C |

For all other adverse events, maximum intensity should be assigned to one of the following categories:

| 0 | = | No adverse event |
| --- | --- | --- |
| 1 | = | An adverse event which is easily tolerated by the subject, causing minimal discomfort and not interfering with everyday activities. |
| 2 | = | An adverse event which is sufficiently discomforting to interfere with normal everyday activities |
| 3 | = | An adverse event which prevents normal, everyday activities. (In a child, such an adverse event would, for example, prevent prevent attendance at school/ kindergarten/ a day-care center and would cause the parents/ guardians to seek medical advice). |

## Assessment of causality

Every effort should be made by the investigator to explain each adverse event and assess its causal relationship, if any, to administration of the study vaccine(s).

The degree of certainty with which an adverse event can be attributed to administration of the study vaccine(s) (or alternative causes, e.g. natural history of the underlying diseases, concomitant therapy, etc.) will be determined by how well the event can be understood in terms of one or more of the following:

- Reaction of similar nature having previously been observed with this type of vaccine and/or formulation.
- The event having often been reported in literature for similar types of vaccines.
- The event being temporally associated with vaccination or reproduced on re-vaccination.

All solicited local (injection site) reactions will be considered causally related to vaccination. Causality of all other adverse events should be assessed by the investigator using the following method:

In your opinion, did the vaccine(s) possibly contribute to the adverse event?

| NO | : | The adverse event is not causally related to administration of the study vaccine(s). There are other, more likely causes and administration of the study vaccine(s) is not suspected to have contributed to the adverse event. |
| --- | --- | --- |
| YES | : | There is a reasonable possibility that the vaccine contributed to the adverse event. |

Non-serious and serious adverse events will be evaluated as two distinct events given their different medical nature. If an event meets the criteria to be determined “serious” (see Section 8.1 for definition of serious adverse event), it will be examined by the investigator to the extent to be able to determine ALL contributing factors applicable to each serious adverse event.

Possible contributing factors include:

- Medical history
- Other medication
- Protocol required procedure
- Lack of efficacy of the vaccine
- Erroneous administration
- Other cause (specify)

## Following-up of adverse events and assessment of outcome

Investigators should follow-up subjects with serious adverse events until the event has resolved, subsided, stabilised, disappeared, the event is otherwise explained, or the subject is lost to follow-up; or, in the case of non-serious adverse events, the subject completes the study. Clinically significant laboratory abnormalities, as well as any adverse event, will be followed up until they have returned to normal, or a satisfactory explanation has been provided. Reports relative to the subsequent course of an adverse event noted for any subject must be submitted to the Study Monitor.

Outcome should be assessed as:

1 = Recovered

2 = Recovered with sequelae

3 = Ongoing at subject study conclusion

4 = Died

5 = Unknown

## Serious adverse events

### Definition of a serious adverse event

A serious adverse event is any untoward medical occurrence that results in death, is life threatening*, results in persistent or significant disability/incapacity† , requires in-patient hospitalisation‡ or prolongation of existing hospitalisation or is a congenital anomaly/birth defect in the offspring of a study subject. In addition, important medical events that may jeopardise the patient or may require intervention to prevent one of the other outcomes listed above should be considered serious. (Examples of such treatments are intensive treatment in an emergency room or at home for allergic bronchospasm; blood dyscrasias or convulsions that do not results in hospitalisation; or development of drug dependency or drug abuse.)

Although not considered as ‘serious adverse events’, cancers should be reported in the same way as serious adverse events.

*Life threatening—definition: An adverse event is life threatening if the subject was at risk of death at the time of the event; it does not refer to an event, which hypothetically might have caused death, if it were more severe.

^†^Disabling/incapacitating—definition: An adverse event is incapacitating or disabling if the event results in a substantial disruption of the subject's ability to carry out normal life functions. This definition is not intended to include experiences of relatively minor medical significance such as headache, nausea, vomiting, diarrhoea, influenza, and accidental trauma (e.g. sprained ankle).

‡Hospitalisation: In general, hospitalisation signifies that the subject has been detained (usually involving at least an overnight stay) at the hospital or emergency ward for treatment that would not have been appropriate in the physician’s office or out-patient setting.

Hospitalisation for either elective surgery related to a pre-existing condition which did not increase in severity or frequency following initiation of the study or for routine clinical procedures¶ (including hospitalisation for “social” reasons) that are not the result of an adverse event need not be considered as adverse events and are therefore not serious adverse events.

¶Routine Clinical Procedure—definition: One which is defined as a procedure which may take place during the study period and should not interfere with the study vaccine administration or any of the ongoing protocol specific procedures.

N.B. If anything untoward is reported during an elective procedure, that occurrence must be reported as an adverse event, either ‘serious’ or ‘non-serious’ according to the usual criteria.

When in doubt as to whether ‘hospitalisation’ occurred or was necessary, the adverse event should be considered serious.

### Reporting serious adverse events

Serious Adverse Events will be reported in a prospective manner during the period starting from the day of administration of the first dose of study vaccine to each subject and ending with the last study visit or performance of the last study procedure or within one month (minimum 30 days) following administration of the last dose of study vaccine for that subject, whatever is longer. Any serious adverse events occurring during this period, whether or not considered to be related to the study vaccine must be reported by the investigator to the appropriate Clinical Safety Group within 1 calendar day (i.e. 24 hours of initial receipt) within GSK Biologicals and MVI, and to the Local Safety Monitor or within 3 calendar days if this period includes a weekend or public holiday.

The investigator will document all available information regarding the serious adverse event on the Serious Adverse Event pages contained in the individual Case Report Form and fax to the GSK Biologicals and MVI Contacts for Serious Adverse Event Reporting. The investigator should not wait to receive additional information to fully document the event before notifying GSK Biologicals and MVI of a serious adverse event. This initial notification should include, as a minimum, sufficient information to permit identification of:

- the reporter
- the subject
- study vaccine(s)
- adverse event(s)
- date of onset

In the event of a death or a serious adverse event determined by the investigator to be related to vaccination, receipt of the fax must be confirmed by telephone call.

The initial fax report should be followed by a full summary utilising the GSK Biologicals Serious Adverse Event pages in the individual Case Report Form detailing relevant aspects of the adverse events in question. Where applicable, information from relevant hospital case records and autopsy reports should be obtained.

***A post-study AE/SAE is defined as any event that occurs outside of the AE/SAE surveillance period for occurrence of adverse events (defined in Section 8.1). Investigators are not obligated to actively seek AEs or SAEs in former study participants.***

***If the investigator learns of any SAE, including a death, at any time after a subject has been discharged from the study, and he/she considers the event reasonably related to a study vaccine, the investigator will promptly notify the Study Contact for Reporting SAEs.***

~~Instances of death, cancer or congenital abnormality in offspring if brought to the attention of the investigator AT ANY TIME after cessation of the~~ ~~study should be reported to the Study Contacts for Serious Adverse Event reporting.~~
**(Amended January 17, 2003)**

| **Study Contacts for Reporting Serious Adverse Events** | |
| --- | --- |
| **Clinical Study Management**  ~~Cornelia Bevilacqua~~  ***Sabine Corachan***  **GlaxoSmithKline Biologicals**  Rue de l'Institut, 89  B-1330 Rixensart – Belgium  Tel. office: ~~32 2 656 77 70~~  ***+32 2 656 92 33***  Fax: 32 2 656 61 60 **(Amended January 17, 2003)** | ***Local GSK Monitor***  ***Laurien Dodsworth***  ***C/o Reliable de Mozambique***  ***C. Postal 1920***  ***Maputo***  ***Mozambique***  ***Tel: +27 824 585 650***  ***Fax: +258 1 451050***  **(Amended January 17, 2003)** |
| **Local Safety Monitor and back-up** | |
| Dr Esperança Sevene  Faculdade de Medicina  Universidade Eduardo Mondlane  Tel: +258 8 2303506  Fax: +258 1 425255  E-mail: [esevene@health.uem.mz](mailto:esevene@health.uem.mz) | Back-up for local safety monitor:  Dr Alda do Rosario Elias Mariano  Faculdade de Medicina  Universidade Eduardo Mondlane  Tel. +258 82 328 053  E-mail: brigith@health.uem.mz |
| **Malaria Vaccine Initiative**  Dr. Regina Rabinovich (Back-up: Dr Filip Dubovsky)  6290 Montrose road, Rockville, Maryland 20852, USA  Tel. 1 301 770 5377; Fax. 1 301 770 53 22 | |
| **Back-up Study Contact at GSK Bio for Reporting Serious Adverse Events** | |
| **Manager Clinical Safety Vaccines** | |
| Dr. Marc Ceuppens | |
| Tel: +32 2 656 87 98 | |
| Fax: +32 2 656 80 09 | |
| Mobile phone for 7/7 day availability: 32 477 404 713 | |

The Principal Investigator will report all serious adverse events to the Local Safety Monitor in his/her capacity as a liaison to the DSMB (see section 5.1.4).

## Pregnancy

Not applicable.

## Treatment of adverse events

Treatment of any adverse event is at the sole discretion of the investigator and according to current Good Medical Practice. The applied measures should be recorded in the CRF of the vaccinee.

# Subject Completion and Drop-out

## Definition

From an analysis perspective, a 'drop-out' is any subject who did not come back for the concluding visit for the double-blind phase of the study (clinic visit 6 at month 8½, day 256). A subject who returns for this concluding visit is considered to have completed the study.

## Procedures for handling drop-outs

Investigators should make an attempt to contact those subjects who do not return for scheduled visits or follow-up. Information gathered should be described on the Study Conclusion page of the CRF and on Medication/Adverse event forms.

## Reasons for drop-out

It should be specified on the Study Conclusion page of the CRF, which of the following possible reasons were responsible for drop-out of the subject from the study:

- Serious adverse event
- Non-serious adverse event
- Protocol violation (specify)
- Consent withdrawal, not due to an adverse event
- Migrated/moved from the study area
- Lost to follow-up
- Other (specify)

# Data Evaluation: criteria for evaluation of objectives

A reporting and analysis plan (RAP) will be developed by CISM and GSK Biologicals in consultation with MVI and submitted to the DSMB for approval prior to breaking the study blind.

For the purposes of data analysis, there will be two distinct data lockpoints for the study, irrespective of cohort:

- Month 8½ (day 256): at the end of the surveillance period for the primary and secondary objectives, double-blind phase.
- Month 21 (day 630): at the end of the safety and efficacy follow-up, open phase. This data lockpoint will be used for the analysis of tertiary objectives.

## Clinical case definition of malaria

The primary case definition of clinical episodes of symptomatic *P.* *falciparum* malaria used for defining the primary endpoint is shown in Table 4. Other definitions of clinical episodes of symptomatic *P.* *falciparum* malaria of different degrees of malaria severity are also included and will be used as specified in the secondary and tertiary efficacy endpoints for the purposes of evaluating vaccine efficacy.

Table 4: Definitions of symptomatic P. falciparum malaria

| Primary Case Definition for malaria episodes | - the presence of *P.* *falciparum* asexual parasitaemia above 2500 per µl on Giemsa stained thick blood films AND - the presence of fever (axillary temperature ≥ 37.5°C) at the time of presentation AND - occurring in a child who is unwell and brought for treatment to a healthcare facility. |
| --- | --- |
| Secondary Case Definition 1 | - the presence of *P.* *falciparum* asexual parasitaemia (**any level of parasitaemia**) on Giemsa stained thick blood films AND - the presence of fever (axillary temperature ≥ 37.5°C) at the time of presentation AND - occurring in a child who is unwell and brought for treatment |
| Secondary Case Definition 2 | - the presence of *P.* *falciparum* asexual parasitaemia (any level of parasitaemia) on Giemsa stained thick blood films AND - **history of fever within 24 hours** or documented fever (axillary temperature ≥ 37.5°C) at the time of presentation AND - occurring in a child who is unwell and brought for treatment |
| Secondary Case Definition 3 | - the presence of ***P.* *falciparum* asexual parasitaemia above 15 000 per µl** on Giemsa stained thick blood films AND - the presence of fever (axillary temperature ≥ 37.5°C) at the time of presentation AND - occurring in a child who is unwell and brought for treatment to a healthcare facility. |
| Anaemia Case Definition | - haematocrit < 25% |

Notes: bold text indicates differences in each definition from the Primary Case Definition for malaria episodes

## Primary endpoint

***Efficacy***

The time to the first clinical episode of symptomatic *P falciparum* malaria (Primary Case Definition for malaria episodes as defined in section 10.1) detected by passive case detection over a 6 month observation period starting 14 days after dose 3 (day 90) in children (1-4 years of age at first vaccination) in Cohort 1 and who have received RTS,S/AS02A malaria vaccine or one of the control vaccine regimes (either hepatitis B vaccine or *Haemophilus influenzae* type b vaccine and 7-valent pneumococcal conjugate vaccine).

## Secondary endpoints

### Cohort 1

***Efficacy***

- The time to the first clinical episode of symptomatic *P falciparum* malaria (Secondary Case Definition 1 as defined in section 10.1) detected by passive case detection over a 6 month observation period starting 14 days after dose 3 (day 90) in children (1-4 years of age at first vaccination) who have received RTS,S/AS02A malaria vaccine or one of the control vaccine schedules (either hepatitis B vaccine or *Haemophilus influenzae* type b vaccine and 7-valent pneumococcal conjugate vaccine).
- The time to the first clinical episode of symptomatic *P falciparum* malaria (Secondary Case Definition 2 as defined in section 10.1) detected by passive case detection over a 6 month observation period starting 14 days after last vaccination (dose 3) in children (1-4 years of age at first vaccination) who have received RTS,S/AS02A malaria vaccine or one of the control vaccine schedules (either hepatitis B vaccine or *Haemophilus influenzae* type b vaccine and 7-valent pneumococcal conjugate vaccine).
- The time to the first clinical episode of symptomatic *P falciparum* malaria (Secondary Case Definition 3 as defined in section 10.1) detected by passive case detection over a 6 month observation period starting 14 days after last vaccination (dose 3) in children (1-4 years of age at first vaccination) who have received RTS,S/AS02A malaria vaccine or one of the control vaccine schedules (either hepatitis B vaccine or *Haemophilus influenzae* type b vaccine and 7-valent pneumococcal conjugate vaccine).
- The total number of clinical episodes of symptomatic *P falciparum* malaria (according to the Primary Case Definition for malaria episodes as defined in section 10.1) over the time at risk (as defined in section 10.7.2.1) during the 6 -month surveillance period (month 2½ to month 8½ inclusive).
- The time to the first presentation at Manhiça District Hospital with anaemia (Anaemia Case Definition as defined in section 10.1) over a 6 month observation period starting 14 days after last vaccination (dose 3) in children (1-4 years of age at first vaccination).
- Presence of anaemia in children (1-4 years of age at first vaccination) (Anaemia Case Definition as defined in section 10.1) at 6½ months after dose 3 (month 8½).
- The number of asexual stage falciparum parasites per µl of blood for each subject at 6½months after dose 3 (month 8½).

***Safety***

- Occurrence of solicited symptoms after each vaccination over a 4-day follow-up period (day of vaccination and 3 subsequent days).
- Occurrence of unsolicited symptoms after each vaccination over a 30-day follow-up period (day of vaccination and 29 subsequent days).
- Occurrence of serious adverse events from the time of first vaccination (month 0) until 6½months post dose 3 (month 8½).

***Immunogenicity***

- Titres of anti-CS antibodies at the following time points for which serology samples are analyzed: day 0, day 90 and month 8½.

### Cohort 2

***Efficacy***

- The time to first malaria infection (first recording of infection of asexual stage falciparum parasites detected by the active detection of infection surveillance or passive case detection, see section 5.2.6) over a period starting 14 days after last vaccination and until 6½months after last vaccination (month 8½).

***Safety***

- Occurrence of solicited symptoms after each vaccination over a 4-day follow-up period (day of vaccination and 3 subsequent days).
- Occurrence of unsolicited symptoms after each vaccination over a 30-day follow-up period (day of vaccination and 29 subsequent days).
- Occurrence of serious adverse events from the time of first vaccination (month 0) until 6½ months post dose 3 (month 8½).

***Immunogenicity***

- Titres of anti-CS antibodies at the following time points for which serology samples are analyzed: days 0, 60 and 90, month 8½.
- Titres of anti-HBs antibodies at the following time points for which serology samples are analyzed: days 0 and 90, month 8½.

## Tertiary Endpoints

### Cohort 1

***Efficacy***

- The total number of clinical episodes of symptomatic *P falciparum* malaria (according to the Primary Case Definition for malaria as defined in section 10.1) over the time at risk (as defined in section 10.7.2.1) during the open surveillance period (months 8½ to 21).
- The total number of clinical episodes of symptomatic *P falciparum* malaria (according to the Primary Case Definition for malaria as defined in section 10.1) over the time at risk (as defined in section 10.7.2.1) during the complete surveillance period (months 2½ to 21).
- Presence or absence of anaemia in children aged 1-4 years of age at first vaccination (Anaemia Case Definition as defined in section 10.1) at month 21 (day 630).
- The number of asexual stage falciparum parasites per µl of blood for each subject at 19 months after dose 3 (month 21).

***Safety***

- Occurrence of serious adverse events during the single-blind surveillance period (from month 10 to month 21 inclusive).

***Immunogenicity***

- Titres of anti-CS antibodies at the following time point for which serology samples are analysed: month 21.

### Cohort 2

***Safety***

- Occurrence of serious adverse events during the single-blind surveillance period (from month 8½ to month 21).

***Immunogenicity***

- Titres of anti-HBs and anti-CS antibodies at the following time points for which serology samples are analyzed: month 21.

## Study groups/data sets to be evaluated

Analyses will be performed on subjects in the Total and According to Protocol (ATP) Cohorts.

***Total cohort for analysis of efficacy***

The Total Cohort will include all subjects enrolled in the study who received at least one dose of the candidate malaria vaccine or control vaccine and for whom data concerning efficacy endpoint measures are available. Note that for the primary endpoint of the trial only those children in Cohort 1 will contribute.

***Intention-to-treat cohort for analysis of vaccine efficacy***

As for the Total Cohort for analysis of vaccine efficacy, the intention-to-treat cohort for analysis of vaccine efficacy will include all subjects enrolled in the study who received at least one dose of the candidate malaria vaccine or control vaccine and for whom data concerning efficacy endpoint measures are available. However, in contrast to the Total Cohort, this cohort will be used specifically for analysis of vaccine efficacy over the period of time at risk starting from first vaccination (day 0) (see Section 10.7.2.2.9).

***Protocol-defined cohort for analysis of efficacy (ATP)***

The protocol-defined cohort for analysis of efficacy will include all evaluable subjects (i.e., those meeting all eligibility criteria, complying with the procedures defined in the protocol, and fulfilling requirements for analysis defined in the RAP) for whom data concerning efficacy endpoint measures are available. Note that for the primary endpoint of the trial only those children in Cohort 1 will contribute.

***Total cohort analysis of safety***

The Total Cohort will include all subjects enrolled in the study for whom data concerning safety endpoint measures are available.

***Protocol-defined cohort for analysis of safety (ATP)***

All reactogenicity analyses will be performed for the Total Cohort.

***Total cohort analysis of immunogenicity***

The Total Cohort will include all subjects enrolled in the study for whom data concerning immunogenicity endpoint measures are available.

***Protocol-defined cohort of immunogenicity (ATP)***

The protocol-defined cohort for analysis of immunogenicity will include all evaluable subjects (i.e., those meeting all eligibility criteria, complying with the procedures defined in the protocol) for whom data concerning endpoint measures are available.

## Estimated sample size

### Cohort 1

The primary objective of this study is to assess the efficacy of the RTS,S/AS02A malaria candidate vaccine against clinical episodes of malaria fitting the primary case definition. 704 evaluable subjects per group are needed in order to have 80% power to detect a lower 95% confidence limit of vaccine efficacy of approximately 15%, assuming a likely *P. falciparum* attack rate during the surveillance period of 11% in the control group and vaccine efficacy of 50% (Table 5). Sample size calculation is done using a method proposed by BlackWelder[[34]](#endnote-35). Taking into account a level of 10% of non-evaluable subjects (e.g. protocol non-compliance, dropout during vaccination schedule), at least 782 subjects should be recruited in each group.

The attack rate estimate of 11% for the control group is derived from a demographic surveillance system in the CISM surveillance area and the morbidity surveillance system at Manhiça Hospital, which has been in operation since mid 1996. The estimate is the average of the attack rates over the last four years (yearly periods of 1998 to 2001). A sample size of 704 per group will provide strong statistical evidence of a treatment benefit (if it exists given the attack rate and vaccine efficacy estimates) at a p value level of 0.002. These sample size estimates were also reproduced using a simulation program that simulates the incidence of clinical malaria episodes based on the maximum likelihood estimate of the hazard assuming a Gompertz distribution of the survival time.

*Algorithm for Sample size program*

- Survival times assuming Gompertz distribution was found to provide the best fit to previously observed data (clinical malaria by passive case detection in children 1 to 4 years of age from 1998-2001). Thus survival in the control group is estimated as: h(t)=exp(λ)*exp(γt),
  where ***h(t) is the hazard function and*** λ and γ are the parameters of the distribution. These parameters are maximum likelihood estimators of the Gompertz fitted model.
  **(Amended January 17, 2003)**
- Based on the survival function:
  ~~S(t) = exp(-exp(λ)/γ*(exp(log(1-log(s)*g/exp(l))/g*t)-1)),~~
  ***S(t)=exp{-exp(λ)*[exp(γ*t)-1]/ γ }***
  random samples from the survival time are drawn taking random samples from the cumulative probability distribution F(t) = 1-S(t). Values from F(t) are simulated using an random uniform distribution between 0 and 1 and converted into survival times using the equation:
  ***t =*** log(1-log(S(t))*γ/exp(λ))/γ
  **(Amended January 17, 2003)**
- The model in the vaccinated group is estimated as:
  h(t)=exp(λ+log(1-VE))*exp(γt)
  where VE is the vaccine efficacy. The procedure to simulate survival times is similar to the method described for the control group above.
- For each simulated trial, an analysis using Cox regression models is made. For this analysis, survival times are censored at 180 days. The empirical power (EP15) is calculated as the proportion of trials where the lower limit of the 95% CI estimate of the VE is greater than 15%. Every set of conditions (N) is simulated 10 000 times to obtain a precision of empirical power.

A sample size of 700 will have 81.5% power to detect a lower confidence interval of the vaccine efficacy greater than 15%, assuming an average rate of clinical malaria (defined as axillary temperature ≥ 37.5°C plus parasitaemia greater than 2500 parasites per µl).

Table 5: Sample size to establish the lower limit of the 95% CI of vaccine efficacy in Cohort 1

| Vaccine efficacy (%) | Attack rate (%) | Lower limit of 95% CI of vaccine efficacy (%) | Number of subjects per group |
| --- | --- | --- | --- |
| 50 | 11 | 30 | 1752 |
|  |  | 25 | 1207 |
|  |  | 20 | 898 |
|  |  | 15 | 704 |
|  |  | 10 | 574 |
|  |  | 5 | 481 |
|  |  | 0.5 | 419 |

There is no plan to extend enrolment in the event that the attack rate is lower than predicted over the period during which the trial will be conducted. If the rate was equal to the lowest recorded in the averaged four yearly rates i.e. 7%, the trial would still be highly powered to detect a difference between groups (power = 80% at the 5% level of significance) but the precision of estimate of effect would be less.

The trial is designed to provide precision about the estimate of effect if the true vaccine efficacy is 50%. This level of efficacy is of prime interest for guiding future development decisions. In the event that the true vaccine efficacy is less, the trial remains sufficiently powered to detect a difference but not to define this with precision. For example, if true vaccine efficacy is 45% the trial will have a power of 90% to detect a difference at the 5% level of significance and similarly if true vaccine efficacy is 40% the trial will have power of 81% at the 5% level of significance.

In addition to the primary objective, data will be analyzed to determine the time to first clinical presentation of a haematocrit below 25%. The average attack rate for anaemia (as defined as a haematocrit of less than 25%) at the Manhiça District Hospital between 1998 to 2001 was 6% over 6 months. A sample size of 704 per group will achieve 68% power to detect a difference of 0.03 between the null hypothesis that both group proportions are 0.06 and the alternative hypothesis that the proportion in the RTS,S/AS02A group is 0.03 using a two-sided log rank test with a significance level of 0.05.

(Sample size calculations were performed using NCSS trial and Pass 2000).

### Cohort 2

The overall aims for Cohort 2 of the study are two fold:

- to assess the efficacy of the RTS,S/AS02A malaria candidate vaccine in delaying time to first asexual falciparum infection.
- to compare the humoral response for anti-HBs antibodies induced by the RTS,S/AS02A malaria candidate vaccine with the humoral response to Engerix-B™ (hepatitis B vaccine).

Table 6 presents the sample sizes required to detect a vaccine efficacy of 50% in the prevention of new infections. A study with a sample size of ***166*** ~~142~~ evaluable subjects per group will have an ***86*** ~~80~~% power to detect a lower confidence interval of 20%. The age distribution of Cohort 2 will reflect that of Cohort 1.
**(Amended January 17, 2003)**

Table 6: Sample size to establish the lower limit of the 95% CI of vaccine efficacy in Cohort 2

| **Number of malaria infected subjects in Control Vaccine Group** | **Number of malaria infected subjects in Malaria Vaccine Group** | **Lower limit of 95% CI for vaccine efficacy** | **Number of subjects per group** |
| --- | --- | --- | --- |
| ~~139~~ ***162*** | ~~69~~ ***81*** | 30% | ~~277~~ ***325*** |
| ~~95~~ ***112*** | ~~48~~ ***56*** | 25% | ~~191~~ ***224*** |
| ~~71~~ ***83*** | ~~36~~ ***42*** | 20% | ~~142~~ ***166*** |
| ~~45~~ ***53*** | ~~23~~ ***27*** | 10% | ~~91~~ ***106*** |
| ~~38~~ ***45*** | ~~19~~ ***22*** | 5% | ~~76~~ ***89*** |
| ~~36~~ ***42*** | ~~18~~ ***21*** | 3% | ~~71~~ ***84*** |
| ~~33~~ ***39*** | ~~17~~ ***19*** | 0.5% | ~~66~~ ***78*** |

**(Amended January 17, 2003)**

This study will compare the immunogenicity of a licensed hepatitis B vaccine (Engerix-B™) and RTS,S/AS02A. A non-inferiority approach will be used to calculate the power of the study for the comparison of seroprotection rates and geometric mean titres of anti-HBs antibodies. From census information gathered by CISM, approximately ***25%*** ~~12.5%~~ of the population will be less than ***24*** ~~18~~ months of age. Therefore the assessment of the anti-HBs antibody immune response will be based on approximately 248 evaluable subjects (124 subjects per group). This sample size will have ***89%*** ~~95%~~ power at a ***2.5%*** ~~5%~~ significance level using a one-sided equivalence test of proportions when the proportion that are seroprotected in the control group (subjects vaccinated with Engerix-B™) is at least 0.95 and the proportion in the RTS,S/AS02A group (subjects of ***24*** ~~18~~ months of age or more) is 0.95 and the maximum allowable difference between these proportions that still results in equivalence (the range of equivalence) is 0.10 (PASS 2000,one sided equivalence test of proportions, alpha = ***2.5%*** ~~5%~~).
**(Amended January 17, 2003)**

Similarly this sample size will provide 99% power to demonstrate at 10% significance level using an equivalence of means test, that the lower limit of a two sided 90% confidence interval on the GMT ratio of anti-HBs antibodies (RTS,S/AS02A vaccine group over Engerix-B™ vaccine group ) is greater than 0.5 (2 fold decrease). (PASS 2000, equivalence of means, alpha = 10%). The standard deviation used for this calculation was 0.591 ***(computed on log base 10 transformed tires)*** and was derived from a GSK Biologicals study (DTPw-HBV-033) in which three doses of vaccine were administered to each subject at 3 months, 4 months and 5 months of age[[35]](#endnote-36).
**(Amended January 17, 2003)**

The 248 subjects available for tests of non-inferiority will include both seronegative and seropositive subjects to the HBsAg at baseline. Table 7 shows the power to meet the non-inferiority criterion if the number of **seronegative only** subjects in the non-inferiority cohort varies. All parameters used for the initial calculations above remain constant except for sample size. All calculations used Pass 2000.

Table 7 : Power to achieve non-inferiority criterion for seroprotection and GMT ratio given the availability of seronegative only subjects in the non-inferiority cohort (124 per group)

| Number of seronegative subjects | Percentage of seronegative subjects in non-inferiority cohort^1^ | Power to achieve non-inferiority criterion for: | |
| --- | --- | --- | --- |
|  |  | Seroprotection | GMT ratio |
| 112 | 90 | 92 | 99 |
| 99 | 80 | 89 | 98 |
| 87 | 70 | 85 | 96 |

1: non-inferiority cohort = 124 subjects from cohort 2 ≥ ***24*** ~~18~~ months of age who receive either RTS,S/AS02A or Engerix-B™. Computed using PASS 2000. **(Amended January 17, 2003)**

Allowing for approximately 20% of subjects who may drop out or who may not be evaluable for analysis, ***416*** ~~356~~ subjects are needed for the analysis of this study cohort objective.
**(Amended January 17, 2003)**

## Final analyses

### Analysis of demographics

Demographic characteristics (age, sex, race) of each study cohort will be tabulated.

The mean age (plus range and standard deviation) by sex of the enrolled subjects, as a whole, and per group, will be calculated.

The distribution of the subjects enrolled among the study sites will be tabulated as a whole and per group.

### Analysis of efficacy

#### Calculation of time at risk

***First surveillance period*** *(Double-blind phase up to day 256, month 8½)*

For each subject in the ATP and Total cohorts, time at risk begins 14 days after the third vaccination while the time at risk for each subject in the ITT cohort begins on day of first vaccination (day 0).

For evaluation of the primary endpoint, end of the time at risk will be the first clinical malaria episode fitting the primary case definition, or on the last day of the surveillance period (day 256, month 8½) if no episode has occurred prior to this. A similarly approach will be used for the other “time to” analyses, which include the secondary endpoint for time to infection (with respect to Cohort 2) and secondary endpoints examining different case definitions of malaria (with respect to Cohort 1). The end of time at risk is determined in the same way for all cohorts (ATP, Total and ITT).

For the evaluation of efficacy against multiple attacks the time at risk will extend to month 8½ and be applied to all cohorts (ATP, Total and ITT).

Time at risk will be adjusted for absence from the study area and anti malaria drug therapy. The monthly home visits will be used to determine any period of travel outside the study area. Absences from the study area of 2 weeks or more will be recorded in multiples of 1 week. The date of departure will be documented. Similarly if treatment for malaria is administered, the subject will not be considered susceptible to malaria infection for a duration 28 days and this period of time will be deducted from the time at risk.

If a subject is lost to follow-up, withdraws or dies, the observations for that subject will be censored at date of withdrawal or death; or at the midpoint between two monthly home visits if the date of withdrawal or death are not available.

***Subjects will be removed from the risk set when they are not at risk by censoring the records and returned to the risk set at a later time. Commercially available software can fit a Cox model, assuming that, in the analysis data set, each subject is represented by one or more observations, each consisting of a time interval, disease and other covariate values of interest over the time interval.*(Amended January 17, 2003)**

***Second surveillance period*** *(Single-blind phase up to month 21)*

During the second surveillance period the time contributed by each individual to the time at risk for each endpoint will be determined in a similar way to the methodology given for that in the first surveillance period. For the ATP and Total cohorts two periods are examined for the endpoints: from month 8½ until month 21 and from month 2½ until month 21. These are specified for each endpoint in the section 10.4. ***For the ITT cohort, the period from day 0 (first vaccination) until month 21 will be examined for the endpoints corresponding to the second surveillance period.***
**(Amended January 17, 2003)**

#### Vaccine efficacy

##### Cohort 1: primary and secondary endpoints

Time to first symptomatic *P. falciparum* malaria over a 6 month period starting 14 days after dose 3 will be examined using Kaplan-Meier curves for both groups and these will be compared using the log-rank test or Wilcoxon test. Cox regression will be used to assess the significance of the group effect and the vaccine efficacy (defined as 1-R, where R is the hazard ratio [RTS,SAS02A:control vaccine]) will be estimated from the hazard ratio of the RTS,S/AS02A group versus the Control group (with 95% CI) adjusting for prognostic/stratifying factors (see section 10.7.2.2.7).

Cox regression assumes proportional hazards throughout the follow-up period. This assumption will be checked by plotting the log of the cumulative hazard against the log of time. Scaled Schonfield residuals may also be plotted to assess extent of non-proportionality of the hazard over time. If there is strong evidence that the hazard ratio is not constant over the surveillance period then alternative approaches to analyse the data will be examined:

- The 6 month surveillance period will be partitioned into three periods based on equal number of events and piecewise Cox regression models fitted to each.
- Cox regression model with time varying covariates.
- Alternative model formulations e.g. accelerated failure time models.

The other secondary endpoints for the evaluation of vaccine efficacy use distinct definitions of clinical episodes. The same procedures as described above will be used to evaluate these clinical case definitions.

##### Cohort 1: secondary endpoint for estimation of vaccine efficacy given multiple episodes per subject

One of the secondary endpoints is the total number of clinical episodes of symptomatic *P. falciparum* malaria over the time at risk during the 6 month surveillance period (months 2½ to 8½ inclusive) (see section 10.3.1).

The analysis of multiple episodes can be handled within the Cox Model framework available in many commercially-available software packages. The ordinary Cox regression estimate of variance for the coefficients treats each observation as independent. This assumption does not hold when a subject can present multiple episodes. An adjustment to the variance estimate to account for the inter-subject correlation has to be made. This can be achieved by using the jackknife to derive a robust variance estimate for the Cox model[[36]](#endnote-37). This is equivalent to the Lin and Wei sandwich estimator for the Cox model[[37]](#endnote-38). The basic procedure is to fit the ordinary Cox model - initially ignoring the correlation - followed by correcting the naïve variance estimate. This approach can fit within commercially available software.

##### Cohort 1: tertiary endpoints for cumulative incidence

The cumulative incidence (subjects with at least one episode of symptomatic *P. falciparum* malaria during the surveillance) over the periods from month 8½ to month 21 and from months 2½ to month 21 will be compared between both groups using Kaplan-Meier curves and Fisher’s Exact test (two-sided) at month 21.

##### Parasite density in Cohort 1: secondary and tertiary endpoints

Two analyses will be performed: Total Cohort and ATP Cohort.

The proportion of children with asexual *P. falciparum* parasitaemia in each group at each of the following time points: 6½ months post dose 3 (month 8½) and 19 months post dose 3 (month 21) will be calculated (with 95% CI). The 95% CI on the difference of the proportion between each group will also be calculated.

The geometric mean parasite density in children in each group that are positive for asexual *P. falciparum* parasitaemia, at the following time points: 6½ months post dose 3 (month 8½) and 19 months post dose 3 (month 21) will be calculated (with 95% CI). The 95% CI on the mean difference between each group will be calculated for 7 months post dose 3 (month 8½) and 19 months post dose 3 (month 21).

##### Analysis of haematocrit values in Cohort 1: secondary and tertiary endpoints

For each group, the number and proportion of children with a haematocrit below 25% at month 8½ or month 21 divided by the total number of children for which haematology samples are available at 6½ months post dose 3 (month 8½) or 19 months post dose 3 (month 21) will be calculated with 95% CI. The 95% CI on the difference of the proportion between each group will also be calculated.

##### Cohort 2: secondary endpoint

Time to first infection of asexual stage *P. falciparum* parasites will be examined using Kaplan-Meier curves for both groups and these will be compared using the log-rank test or Wilcoxon test. Cox regression will be used to assess the significance of the group effect and the vaccine efficacy (defined as 1-R, where R is the hazard ratio [RTS,S/AS02A:control vaccine]) will be estimated from the hazard ratio of the RTS,S/AS02A group versus the Control group (with 95% CI) adjusting for prognostic/stratifying factors (see section 10.7.2.2.7).

##### Adjustment for covariates

The risk for malaria infection and development of clinical disease depends on numerous factors related to the parasite, host and vector biology. Estimates of the vaccine efficacy will be made, firstly based on treatment group and secondly adjusted for:

- age: continuous variable.
- bednet use: dichotomous yes/no variable.
- geographical area (“barrios” local government areas): categorical variable.
- distance from health centre (in kilometers): continuous variable.
- IFAT score (anti-parasite antibody levels) at baseline: continuous variable.

An exploratory examination of the marginal effect each of the above covariates have on the treatment estimate may also be undertaken.

To date there has not been any evidence that the pre-vaccination anti-HBs serostatus of subjects impacts on the magnitude of CS response after vaccination with RTS,S/AS02A. However, in the event that anti-HBs serostatus prior to vaccination has an impact on magnitude of CS response then this will be explored as an explanatory variable of vaccine effect.

##### Comparability between study sites

The anti-parasite antibody levels measured by IFAT at screening will be assessed to determine the comparability between study sites in terms of malaria transmission intensity.

##### Exploratory analyses

*Intention-to treat analysis for vaccine efficacy*

***An intention-to-treat analysis will be conducted for all clinical efficacy endpoints incorporating a “time to” analysis (as defined in sections 10.2, 10.3 and Table 4) for the periods of time at risk as defined in section 10.7.2.1. The methodology to be used for this analysis is detailed in section 10.7.2.2.1.***
**(Amended January 17, 2003)**

*Analysis of spleen size*

Mean spleen size (size classified according to the Hackett scale, see section 5.7) will be calculated, with 95% CI, for each group at months 8½and 21. Little background data is available on this indicator of malaria exposure/disease in the study population therefore a descriptive analysis of the variable will be performed.

*~~Intention-to treat analysis for vaccine efficacy~~*

~~An intention-to-treat analysis for the primary efficacy endpoint (see section 10.2 for definition of the primary endpoint) will be prepared. The period of time at risk will be calculated as for the primary efficacy endpoint except that the period will start from first vaccination (day 0) and will last for 8½ months. The same analytical procedures as described in section 10.7.2.2.1 will be used for this analysis.~~ **(Amended January 17, 2003)**

*Analysis of parasite genotyping*

The strain specificity of the vaccine will be examined by calculating vaccine efficacy against time to first infection with the NF54 parasite strain and against time to first infection with all other strains. Confidence intervals will be put around the estimates of efficacy. The two estimates will be descriptive and the trial is not powered to detect a difference in effect.

### Analysis of safety

All safety and reactogenicity analyses will be performed for the Total Cohort.

The overall percentage of subjects reporting any adverse event after vaccination will be tabulated with exact 95% confidence intervals, by type of adverse event; by severity (any grade, grade 3 only); by relationship (No, Yes, respectively).

The overall percentage of doses for which any adverse event is reported after vaccination will be tabulated with exact 95% confidence intervals, by type of adverse event; by severity (any grade, grade 3 only); by relationship (No, Yes, respectively).

The proportion of subjects with at least one report of unsolicited adverse event, classified by MedRA, reported up to 30 days after vaccination, will be tabulated. The same tabulation will be performed for unsolicited adverse events with relationship to vaccination.

Serious adverse events and discontinuation due to adverse event(s) will be described in detail.

Serum ALT, bilirubin creatinine levels, hematocrit values and body weight that are outside of the reference ranges will be described.

### Analysis of immunogenicity

Two analyses will be performed: Total Cohort and ATP Cohort.

For each treatment group, the seropositivity rate for anti-CS antibodies (proportion of subjects with anti-CS antibody titres of ≥ 1 µg/ml) and its 95% CI will be tabulated by vaccine dose. Antibody titres will be summarized by geometric mean titres (GMTs) of CS repeat antibodies (anti R32LR) measured in µg/ml. 95% CI of GMTs will be calculated for all time points at which serology samples are taken. Reverse cumulative curves will be plotted for antibody titres measured at month8½. GMT calculations are performed by taking the anti-log of the mean of the log titre transformations ***(log base 10)***. Antibody titres of < 1µg/ml will be given an arbitrary value of 0.5 µg/ml for the purpose of GMT calculation.
**(Amended January 17, 2003)**

For each treatment group in Cohort 2, the seroprotection rate for anti-HBs antibodies (proportion of subjects with anti-HBs antibody titres of ≥ 10 mIU/ml) and its 95% CI will be tabulated by vaccine dose. GMTs for anti-HBs antibodies measured in mIU/ml with 95% CI will be calculated for each group at each time point when a serology sample is available. Reverse cumulative curves will be plotted for anti-HBs antibody titres measured at month 8½. GMTs for anti-RF1-like antibodies, measured in EL.U/ml, with 95% CI will be calculated for each group at each time point when serology sample is available. For GMT calculations, antibody titres below the cut-off of the assay (10 mIU/ml) will be given an arbitrary value of half the cut-off for the purpose of calculating the GMTs.

Number and percentage, with 95% CI, of children of 18 months of age or more at first vaccination with seroprotective levels of anti-HBs antibodies (seroprotective levels are defined as anti-HBs titres of 10 mIU/l or greater) at day 90 will be computed.

***This study will compare the immunogenicity of (Engerix-B™) and RTS,S/AS02A. A non-inferiority approach will be used to calculate the power of the study for the comparison of seroprotection rates and geometric mean titres of anti-HBs antibodies. From census information gathered by CISM, approximately 25% of the population will be less than 24 months of age. Therefore the assessment of the anti-HBs antibody immune response will be based on approximately 248 evaluable subjects (124 subjects per group). This sample size will have 89% power at a 2.5% significance level using a one-sided equivalence test of proportions when the proportion that are seroprotected in the control group (subjects vaccinated with Engerix-B™) is at least 0.95 and the proportion in the RTS,S/AS02A group (subjects of 24 months of age or more) is 0.95 and the maximum allowable difference between these proportions that still results in equivalence (the range of equivalence) is 0.10 (PASS 2000, one sided equivalence test of proportions, alpha = 2.5%).*** ~~The difference between seroprotection rates at month 4 for anti-HBs antibodies between both groups will be calculated as well as the 90% CI around these difference. If the lower limit of the two-sided 90% CI (corresponding to a one-sided 95% CI) is greater than –10%, non-inferiority of RTS,S/AS02A compared to Engerix-B™ will have been demonstrated in this study.~~
**(Amended January 17, 2003)**

GMTs for anti-HBs antibody titres at month 4 between both groups will be calculated as well as the 90% CI around the ratio of GMTs (RTS,S/AS02A / Engerix-B™). If the lower limit of the two-sided 90% CI (corresponding to a one-sided 95% CI) is greater than 0.5, then non-inferiority of RTS,S/AS02A compared to Engerix-B™ will have been demonstrated.

The assessment of non-inferiority for seroprotection rates and GMT ratios will be conducted on all subjects (regardless of whether subjects are seropositive or seronegative for HBsAg at baseline) and on HBsAg seronegative subjects only.

## Planned interim analysis

No interim analysis is planned.

# Administrative Matters

To comply with Good Clinical Practice important administrative obligations relating to investigator responsibilities, monitoring, archiving data, audits, confidentiality and publications must be fulfilled. See Appendix B for details.

# References

Appendix A: World Medical Association Declaration of Helsinki

**Recommendations guiding physicians**

**in biomedical research involving human subjects**

**Adopted by the 18th World Medical Assembly**

**Helsinki, Finland, June 1964**

**and amended by the**

**29th World Medical Assembly**

**Tokyo, Japan, October 1975**

**35th World Medical Assembly**

**Venice, Italy, October 1983**

**41st World Medical Assembly**

**Hong Kong, September 1989**

**and the**

**48th General Assembly**

**Somerset West, Republic of South Africa, October 1996**

**INTRODUCTION**

**It is the mission of the physician to safeguard the health of the people. His or her knowledge and conscience are dedicated to the fulfillment of this mission.**

**The Declaration of Geneva of the World Medical Association binds the physician with the words, "The health of my patient will be my first consideration," and the International Code of Medical Ethics declares that, "A physician shall act only in the patient's interest when providing medical care which might have the effect of weakening the physical and mental condition of the patient."**

**The purpose of biomedical research involving human subjects must be to improve diagnostic, therapeutic and prophylactic procedures and the understanding of the etiology and pathogenesis of disease.**

**In current medical practice most diagnostic, therapeutic or prophylactic procedures involve hazards. This applies especially to biomedical research.**

**Medical progress is based on research which ultimately must rest in part on experimentation involving human subjects.**

**In the field of biomedical research a fundamental distinction must be recognized between medical research in which the aim is essentially diagnostic or therapeutic** **for a patient, and medical research, the essential object of which is purely scientific and without implying direct diagnostic or therapeutic value to the person subjected to the research**.

**Special caution must be exercised in the conduct of research which may affect the environment, and the welfare of animals used for research must be respected.**

**Because it is essential that the results of laboratory experiments be applied to human beings to further scientific knowledge and to help suffering humanity, the World Medical Association has prepared the following recommendations as a guide to every physician in biomedical research involving human subjects. They should be kept under review in the future. It must be stressed that the standards as drafted are only a guide to physicians all over the world. Physicians are not relieved from criminal, civil and ethical responsibilities under the laws of their own countries.**

**I. BASIC PRINCIPLES**

1. **Biomedical research involving human subjects must conform to generally accepted scientific principles and should be based on adequately performed laboratory and animal experimentation and on a thorough knowledge of the scientific literature.**
2. **The design and performance of each experimental procedure involving human subjects should be clearly formulated in an experimental protocol which should be transmitted for consideration, comment and guidance to a specially appointed committee independent of the investigator and the sponsor provided** **that this independent committee is in conformity with the laws and regulations of the country in which the research experiment is performed.**
3. **Biomedical research involving human subjects should be conducted only by scientifically qualified persons and under the supervision of a clinically competent medical person. The responsibility for the human subject must always rest with a medically qualified person and never rest on the subject of research, even though the subject has given his or her consent.**
4. **Biomedical research involving human subjects cannot legitimately be carried out unless the importance of the objective is in proportion to the inherent risk to the subject.**
5. **Every biomedical research project involving human subjects should be preceded by careful assessment of predictable risks in comparison with foreseeable benefits to the subject or to others. Concern for the interests of the subject must always prevail over the interests of science and society.**
6. **The right of the research subject to safeguard his or her integrity must always be respected. Every precaution should be taken to respect the privacy of the subject and to minimize the impact of the study on the subject's physical and mental integrity and on the personality of the subject.**
7. **Physicians should abstain from engaging in research projects involving human subjects unless they are satisfied that the hazards involved are believed to be predictable. Physicians should cease any investigation if the hazards are found to outweigh the potential benefits.**
8. **In publication of the results of his or her research, the physician is obliged to preserve the accuracy of the results. Reports of experimentation not in accordance with the principles laid down in this Declaration should not be accepted for publication.**
9. **In any research on human beings, each potential subject must be adequately informed of the aims, methods, anticipated benefits and potential hazards of the study and the discomfort it may entail. He or she should be informed that he or she is at liberty to abstain from participation in the study and that he or she is free to withdraw his or her consent to participation at any time. The physician should then obtain the subject's freely‑given informed consent, preferably in writing.**
10. **When obtaining informed consent for the research project the physician should be particularly cautious if the subject is in a dependent relationship to him or her or may consent under duress. In that case the informed consent should be obtained by a physician who is not engaged in the investigation and who is completely independent of this official relationship.**
11. **In case of legal incompetence, informed consent should be obtained from the legal guardian in accordance with national legislation. Where physical or mental incapacity makes it impossible to obtain informed consent, or when the subject is a minor, permission from the responsible relative replaces that of the subject in accordance with national legislation.**

**Whenever the minor child is in fact able to give a consent, the minor's consent must be obtained in addition to the consent of the minor's legal guardian.**

1. **The research protocol should always contain a statement of the ethical considerations involved and should indicate that the principles enunciated in the present Declaration are complied with.**

**II. MEDICAL RESEARCH COMBINED WITH PROFESSIONAL CARE
(Clinical research)**

1. **In the treatment of the sick person, the physician must be free to use a new diagnostic and therapeutic measure, if in his or her judgement it offers hope of saving life, re-establishing health or alleviating suffering.**
2. **The potential benefits, hazards and discomfort of a new method should be weighed against the advantages of the best current diagnostic and therapeutic methods.**
3. **In any medical study, every patient - including those of a control group, if any - should be assured of the best proven diagnostic and therapeutic method. This does not exclude the use of inert placebo in studies where no proven diagnostic or therapeutic method exists.**
4. **The refusal of the patient to participate in a study must never interfere with the physician–patient relationship.**
5. **If the physician considers it essential not to obtain informed consent, the specific reasons for this proposal should be stated in the experimental protocol for transmission to the independent committee (I, 2).**
6. **The Physician can combine medical research with professional care, the objective being the acquisition of new medical knowledge, only to the extent** **that medical research is justified by its potential diagnostic or therapeutic value for the patient.**

**III. NON-THERAPEUTIC BIOMEDICAL RESEARCH INVOLVING HUMAN SUBJECTS
(Non-clinical biomedical research)**

1. **In the purely scientific application of medical research carried out on a human being, it is the duty of the physician to remain the protector of the life and health of that person on whom biomedical research is being carried out.**
2. **The subjects should be volunteers ‑ either healthy persons or patients for whom the experimental design is not related to the patient's illness.**
3. **The investigator or the investigating team should discontinue the research if in his/her or their judgement it may, if continued, be harmful to the individual.**
4. **In research on man, the interest of science and society should never take precedence over considerations related to the well being of the subject**.

Appendix B: Administrative Matters

**I. Responsibilities of the Investigator**

- To ensure that he/she has sufficient time to conduct and complete the study and has adequate staff and appropriate facilities which are available for the duration of the study and to ensure that other studies do not divert essential subjects or facilities away from the study at hand.
- To submit an up-to-date curriculum vitae and other credentials (e.g. medical license number in the United States) to the sponsor and—where required—to relevant authorities.
- To acquire the normal ranges for laboratory tests performed locally and, if required by local regulations, obtain the Laboratory License or Certification.
- To retain no clinical samples (including serum samples) on site without the approval of GlaxoSmithKline Biologicals and the express written informed consent of the subject and/or the subject’s legally authorized representative.
- To perform no other biological assays at the investigator site except those described in the protocol or its amendment(s).
- To prepare and maintain adequate case histories designed to record observations and other data pertinent to the study.
- To conduct the study in compliance with the protocol and appendices.
- To cooperate with a representative of GlaxoSmithKline Biologicals in the monitoring process of the study and in resolution of queries about the data.

**II. Protocol Amendments and modifications**

- No changes to the study protocol will be allowed unless discussed in detail with the GlaxoSmithKline Biologicals' Clinical Project Manager and filed as an amendment/modification to this protocol.
- Any amendment/modification to the protocol will be adhered to by the participating center(s) and will apply to all subjects. Written IRB/IEC approval of protocol amendments is required prior to implementation; modifications are submitted to IRBs/IECs for information only.

**III. Sponsor’s Termination of Study**

GlaxoSmithKline Biologicals reserves the right to discontinue the clinical study at any time for medical or administrative reasons. When feasible, a 30-day written notification will be tendered.

**IV. Remote Data Entry Instructions**

Study data for each subject will be recorded on Remote Data Entry screens.

**Remote Data Entry**

Remote Data Entry (RDE) will be used as the method for data collection, which means that subject information will be entered into a computer at the investigational site. The site will be capable of modifying the data to assure accuracy with source documentation. All new/updated information will be reviewed and verified by a sponsor’s representative. This information will finally be stored in a central database maintained by the sponsor. At the conclusion of the study, the sponsor will archive the study data in accordance with internal procedures. In addition, the investigator will be provided with a CD-ROM of the final version of the data generated at the investigational site.

Specific instructions for use of RDE will be included in the training material provided to the investigational site.

**V. Monitoring by GlaxoSmithKline Biologicals** **(i.e. the Sponsor)**

Monitoring visits by a professional representative of the sponsor will be scheduled to take place before entry of the first subject, during the study at appropriate intervals and after the last subject has completed. It is anticipated that monitoring visits will occur at a minimum frequency of once every eight weeks.

These visits are for the purpose of confirming that GlaxoSmithKline Biologicals sponsored studies are being conducted in compliance with the relevant Good Clinical Practice regulations/ guidelines, verifying adherence to the protocol and the completeness and exactness of data entered on the CRF/RDE screens and Vaccine Inventory Forms. The monitor will verify CRF/RDE entries by comparing them with the source data/documents that will be made available by the investigator for this purpose. Data to be recorded directly into the CRF/RDE screens will be specified in the source documentation agreement form that is contained in both the monitor’s and investigator’s study file. In cases of RDE, the monitor will mark completed and approved screens at each visit. At the end of the monitoring visit, the monitor will transmit the electronic data. The investigator must ensure provision of reasonable time, space and adequate qualified personnel for monitoring visits.

All data, information and results arising out of the study will be held in strict confidence by the authorised staff at the Investigator’s site and will only be used for the purposes of the study. Any personally identifiable information at the investigator’s site will be held and processed under secure conditions with access limited to appropriate sponsor’s staff or other authorised agents, having a requirement to maintain the confidentiality of the information.

At the end of the study, only authorised individuals employed by the Investigator will be able to identify study subjects and relate any data, information or results to a specific study subject. Study subjects will not be disclosed to any person, except for the purposes as described in the protocol and in the event of a medical emergency or if required by law. Following the conclusion of the study, GSK Biologicals, all other designated collaborators and drug regulatory or official agencies (e.g. FDA) will have access to study subject data but all such study subject data will remain anonymous.

The subject or his/her guardian may be entitled under law to access their data and to have any justifiable corrections made. If they wish to do so, this should be requested from the investigator conducting the study.

**VI. Archiving of Data**

The investigator/ institution should maintain all study documentation until at least 2 years after the last approval of a marketing application in an ICH region and until there are no pending or contemplated marketing applications in an ICH region or at least 2 years have elapsed since the formal discontinuation of the clinical development of the investigational product. These documents should be retained for a longer period however if required by the applicable regulatory requirements or by an agreement with the sponsor. It is the responsibility of the sponsor to inform the investigator/institution as to when these documents no longer need to be retained. The investigator/ institution should take measures to prevent accidental or premature destruction of these documents. In studies which employ Remote Data Entry, these data will be will be in the CD Rom format.

Similarly, the sponsor-specific study documentation should be retained until at least 2 years after the last approval of a marketing application in an ICH region and until there are no pending or contemplated marketing applications in an ICH region or at least 2 years have elapsed since the formal discontinuation of clinical development of the investigational product. These documents should be retained for a longer period however if required by the applicable regulatory requirements or if needed by the sponsor. The sponsor should inform the investigator/institution in writing of the need for record retention and should notify the investigator/institution in writing when the study-related records are no longer needed.

**VII. Audits**

For the purpose of compliance with Good Clinical Practice and Regulatory Agency Guidelines it may be necessary for GlaxoSmithKline Biologicals or a Drug Regulatory Agency to conduct a site audit. This may occur at any time from start to after conclusion of the study.

When an investigator signs the protocol, he agrees to permit Drug Regulatory Agency and GlaxoSmithKline Biologicals' audits, providing direct access to source data/ documents. Furthermore, if an investigator refuses an inspection, his data will not be accepted in support of a New Drug Registration and/or Application.

GlaxoSmithKline Biologicals has a substantial investment in clinical studies. Having the highest quality data and studies are essential aspects of vaccine development. GlaxoSmithKline Biologicals has a Regulatory Compliance staff who audit investigational sites. Regulatory Compliance assesses the quality of data with regard to accuracy, adequacy and consistency. In addition, Regulatory Compliance assures that GlaxoSmithKline Biologicals sponsored studies are in accordance with the Good Clinical Practices and that relevant regulations/guidelines are being followed.

To accomplish these functions, Regulatory Compliance selects investigational sites to audit. These audits usually take 1 to 2 days. The GlaxoSmithKline Biologicals’ audits entail review of source documents supporting the adequacy and accuracy of CRFs, review of documentation required to be maintained, and checks on vaccine accountability. The GlaxoSmithKline Biologicals’ audit therefore helps prepare an investigator for a possible regulatory agency inspection as well as assuring GlaxoSmithKline Biologicals of the validity of the database across investigational sites.

The Inspector will be especially interested in the following items:

- Log of visits from the sponsor's representatives
- IRB/IEC approval
- Vaccine accountability
- Approved study protocol and amendments
- Informed consent of the subjects (written or witnessed oral consent)
- Medical records supportive of CRF data
- Reports to the IRB/IEC and the sponsor
- Record retention

GlaxoSmithKline Biologicals will gladly help investigators prepare for an inspection.

**VIII. Confidentiality and Publication**

You agree that all information communicated to you by GlaxoSmithKline Biologicals is the exclusive property of GlaxoSmithKline Biologicals and you will ensure that the same shall be kept strictly confidential by you or any other person connected with the work and shall not be disclosed, either orally or in written form, by you or such person to any third party without the prior written consent of GlaxoSmithKline Biologicals. You shall communicate the results of the work promptly to GlaxoSmithKline Biologicals.

We agree that you shall have the right to publish or permit the publication of any information or material relating to or arising out of the work after prior submission to us provided that if we shall so request you will delay publication for a maximum of six months to enable us to protect our rights in such information or material. Any proposed publication or presentation (e.g. manuscript, abstract or poster) for submission to a journal or scientific meeting, should be sent to GSK Bio and Malaria Vaccine Initiative (MVI) no less than 30 days prior to submission/presentation, together with confirmation that any other author(s) has seen and agreed the proposed publication/ presentation. GlaxoSmithKline Biologicals and MVI will undertake to comment on such documents within four weeks.

Any publication or presentation shall state the following in an appropriate location: ”Funded in part by the Malaria Vaccine Initiative at PATH.”

All rights and interests worldwide in any inventions, know-how or other intellectual or industrial property rights which arise during the course of and/or as a result of the clinical study which is the subject of this Protocol or which otherwise arise from the information or materials supplied under this Agreement, shall be assigned to, vest in and remain the property of GlaxoSmithKline plc.

Appendix C: Overview of the recruitment plan

Four months prior to enrolment a specific information policy towards the targeted population will be implemented. This will consist of a step by step approach, starting with the administrative and senior leaders of the community and the Organization of Mozambican Women (OMW) as well as health personnel from the Manhiça District. The sessions will explain the problem of malaria to this community, the current strategies for its control, as well as the limitations of these strategies. The need and the difficulties of developing a vaccine against malaria will be discussed, as well as an outline of the proposed trials, including the rationale, the background data available and the study objectives. Particular attention will be paid to study procedures including immunization and blood collection. In that respect a full discussion on the purpose of blood collection and the associated risks will be carried out.

Following the above information procedures, two lists (one for each cohort) of the eligible children aged 1 to 4 at the expected day of first immunization will be produced from our census database. The list for Cohort 1 will start with children coming from the “bairos” (local government areas) with known high malaria attack rates and closest to the hospital. Once the targeted number of children have been listed, individual invitations will be delivered by CISM field assistants to each child, requesting them to attend in groups of 10 on a specified date at a designated area of their “bairo” or Ilha Josina Health Facility.

Specially trained senior field assistants will inform the parents/guardians again about the study objectives and procedures including immunization and blood collection and they will be encouraged to ask and clarify questions about the trial. Parents/guardians will then be invited to attend 2-3 days later at a specifically designated area of Manhiça District Hospital or Ilha Josina Health Facility for screening. It is expected that all children will be recruited from areas that are within walking distance of the Manhiça District Hospital or Ilha Josina Health Facility. Should any child require transportation, this will be arranged and provided by CISM. On arrival to Manhiça District Hospital or Ilha Josina Health Facility for screening, an attendance record will be taken for each child. Senior assistants will seek individual informed consent for each child from the parents/guardians in privacy. The parents/guardians’ understanding of the Informed Consent form will be verified by use of an oral assessment questionnaire (outlined in CISM SOPs). The parents/guardians and the witness will sign the Informed Consent form.

Appendix D: Handling of Biological Samples Collected by the Investigator

**Instructions for Handling of Serum or Plasma Samples**

When materials are provided by GlaxoSmithKline Biologicals, it is mandatory that all clinical samples (including serum samples) be collected using exclusively those materials in the appropriate manner. The use of other materials could result in the exclusion of the subject from analysis. The investigator must ensure that his/her personnel and the laboratory(ies) under his/her supervision comply with this requirement.

1. **Collection**

The whole blood (by capillary or venous route) should be collected observing appropriate aseptic conditions. It is recommended that Microtainer® tubes with integrated serum separator (e.g. Becton-Dickinson Microtainer®) be used so as to minimise the risk of haemolysis and to avoid blood cell contamination of the serum when transferring to standard tubes.

**2. Serum separation**

These guidelines aim to ensure high quality samples by minimising the risk of haemolysis, blood cell contamination of the sample or serum adverse cell toxicity at testing.

- For separation of serum using Microtainer® tubes, the instructions provided by the manufacturer should be followed. Siliconised tubes should never be used (cell toxicity). Often the manufacturer’s instruction states that the relative centrifugal acceleration known also as “G” must be “between 1000 and 1300 G” with tubes spinning for 10 minutes. Error in calculation of centrifuge speed can occur when laboratory personnel confuse “G” acceleration with “RPM” (revolutions per minute). The speed of centrifugation must be calculated using the “G” rate provided in the manufacturer’s instructions and the radius of the centrifuge head. After measuring the radius of the centrifuge machine, a speed/acceleration normograph must be employed to determine the centrifuge speed in “RPM”.
- Following separation, the serum should be aseptically transferred to the appropriate standard tubes using a sterile disposable pipette. The serum should be transferred as gently as possible to avoid blood cell contamination.
- The tube should not be overfilled (max. 3/4 of the total volume) to allow room for expansion upon freezing.
- The tube should be identified by the appropriate, provided label (see point 3).

**3. Labelling**

- The standard labels provided by GlaxoSmithKline Biologicals should be used to label each serum/plasma sample.
- If necessary, any hand-written additions to the labels should be made using indelible ink.
- The label should be attached to the tube as follows (see diagram):
- first attach the paper part of the label to the tube
- then wrap the label around the tube so that the transparent, plastic part of the label overlaps with the label text and bar code and shields them.

This will ensure optimal label attachment.

- Labels should not be attached to caps.

4. Sorting and storage

- Tubes should be placed in the GlaxoSmithKline Biologicals’ carton or cardboard box in numerical order from left to right, starting from the lower left hand corner, beginning with the pre vaccination samples series, then with the post vaccination sample series.
- The tubes of serum should be stored in a vertical position at a temperature between ‑20°C and ‑70°C until shipment to GlaxoSmithKline Biologicals. Wherever possible, a backup facility for storage of serum/plasma samples should be available.
- A standard Serum Listing Form, specifying the samples being shipped for individual subjects at each timepoint, should be prepared for each shipment. A copy of this list should be retained at the study site, while the original should be sealed in a plastic envelope and shipped with the serum samples. Once flight details are known, a standard Specimen Transfer Form must be completed and faxed to GlaxoSmithKline Biologicals to the number provided below. A copy of the Specimen Transfer Form must be in the parcel.[[38]](#footnote-2)

GLAXOSMITHKLINE BIOLOGICALS

Attn. Mr. Emmanuel Dael/Mr. Philippe Hoebregts

Clinical Immunology

R & D Department/Building 44

Rue de l'Institut, 89

B-1330 Rixensart - Belgium

Telephone: 32-2-656 8949/32-2-656 9718

Fax: 32-2-656 6052

The central study monitor, ***Sabine Corachan*** ~~Cornelia Bevilacqua~~, should be informed 2 days before any shipment (phone office: ***32 2 656 92 33*** ~~32 2 656 77 70~~ or fax 32 2 656 61 60).

Appendix E: Shipment of Biological Samples

**Instructions for Shipment of Serum Samples**

Serum samples should be sent to the sponsor at regular intervals. The frequency of shipment of samples should be decided upon by the Site Monitor, Clinical Study Management and the investigator prior to the study start.

Serum samples should always be sent by air, preferably on a Monday, Tuesday or Wednesday, unless otherwise requested by the sponsor.

Serum samples must be placed with dry ice (‑20°C) in a container complying with IATA requirements. The completed standard serum listing form should always accompany the shipment.

The container must be clearly identified with the labels provided by GlaxoSmithKline Biologicals specifying the shipment address and the storage temperature (‑20°C).

The airway bill should contain the instruction for storage of samples at ‑20°C.

A "proforma" invoice, stating a value for customs purposes only, should be prepared and attached to the container. This document should contain the instruction for storage of samples at ‑20°C.

Details of the shipment, including: * airway bill number

* flight number

* flight departure and arrival times

should be sent by fax, two days before shipment, to:

GLAXOSMITHKLINE BIOLOGICALS

Attn. Mr. Emmanuel Dael/Mr. Philippe Hoebregts

Clinical Immunology

R & D Department/Building 44

Rue de l'Institut, 89

B-1330 Rixensart - Belgium

Telephone: 32-2-656 8949/32-2-656 9718

Fax: 32-2-656 6052

The central study monitor, ***Sabine Corachan*** ~~Cornelia Bevilacqua~~, should be informed 2 days before any shipment (phone office: ***32 2 656 92 33*** ~~32 2 656 77 70~~ or fax 32 2 656 61 60).

Appendix F: Laboratory Assays

***Serology testing***

Serological responses will be measured principally by evaluating antibody responses to HBsAg and to CSP repeats (anti R32LR). Serum for antibody determination will be collected at the timepoints defined in the flowcharts in sections 5.3 and 5.5.

Antibody levels against anti-CS will be measured at WRAIR by standard ELISA methodology using plate adsorbed R32LR antigen with a standard reference antibody as a control according to SOPs from the WRAIR. Results will be reported in µg/ml.

Antibody to hepatitis B surface antigen will be measured at GSK Bio using a commercially available ELISA immunoassay (AUSAB EIA test kit from Abbott) according to the manufacturer’s recommendations. Results will be reported in mIU/ml.

HBsAg will be measured using a commercially available ELISA (ETI-MAK-4 DIASORIN®) and according to the manufacturer’s recommendations The tests will be performed at the Microbiology Service at Hospital Clinic, Barcelona, Spain, following laboratory SOPs.

Antibodies against asexual falciparum parasites will be measured at CISM using previously published IFAT methods^22,23^ and following laboratory SOPs. Results will be reported as presence or absence of anti-parasite antibodies.

Antibody to RF1-like antibodies will be measured at GSK Bio using an in-house competitive ELISA:

- *Principle of the assay:* Two-fold dilutions of the test samples and the reference serum are performed in a buffer and mixed 1:1 with a fixed amount of RF1 mAb. Mixtures are then incubated for 1.5 hours at 37°C on HBsAg-coated plates. After a washing step, biotin-conjugated sheep anti-mouse Ig is added and incubated for 30 min at 37°C. After an amplification step with streptavidin-POD for 30 minutes at 37°C, plates are then washed and incubated with a solution of tetramethylbenzidine substrate for 10 minutes at room temperature. The reaction is stopped with 0.5 N sulfuric acid and optical density is measured at 450 nm.
- *Calculation method:* The amount of antibody competing with RF1 mAb for binding to HBsAg is quantified by comparison with the reference serum using Softmax Pro (4 parameters equation). The results are expressed in Elisa Units per millilitre (EL.U/ml).

***Determination of parasitaemia***

Estimates of asexual falciparum parasite density will be made at CISM according to laboratory SOPs. Two slides will be air dried, stained with Giemsa and read on a light microscope with a x50 oil immersion lens and x10 eyepieces. Parasite density will be assessed by counting the number of asexual stage parasites per 200 leukocytes. Slides will be declared negative only after 200 leukocytes have been read. Parasite numbers will be converted to a count/µl by assuming a standard leukocyte count of 8000/µl. All slides will be read twice independently, and a third time if the ratio of densities from the first two is greater than 1.5 or smaller than 0.67 or if there is a discrepancy in positivity. If less than 30 parasites are counted a third reading by a different reader will be done if the difference in the number of parasites is greater than 10. The definitive result will be based on the majority verdict for positivity and the geometric mean of the positive densities for positive slides.

***Determination of parasite genotyping by polymerase chain reaction***

Each time a malaria smear is obtained ***during the surveillance period for primary and secondary endpoints (day 90 up to and including day 256)***, whether for routine collections or to evaluate clinical symptoms, a few drops of blood will be collected onto filter paper for PCR analysis according to the SOP for this procedure. This collection will be noted on the CRF appropriate to the situation. Samples will be collected, stored individually in plastic bags according to SOP, and transferred to a separate laboratory where PCR analysis will be performed. DNA will be extracted subsequently using standard methods. To determine the effect of RTS,S on parasite subpopulations, the distribution of variable alleles of vaccine and non-vaccine type in the CSP gene will be studied in parasites obtained from subjects vaccinated with the RTS,S vaccine or control vaccine using PCR and specific oligonucleotide or sequencing techniques.
**(Amended January 17, 2003)**

***Biochemical and haematological analyses***

Haematological and biochemical testing will be done at CISM in Mozambique, following laboratory SOPs.

Appendix G: Vaccine Supplies, Packaging and Accountability

It is under no circumstances permitted to use supplies for purposes other than those specified in the protocol.

1. Vaccine supplies

The investigator will be supplied by the sponsor the following amounts of numbered doses of study vaccines, sufficient to administer 3 dose(s) to all subjects as described in the present protocol:

- ***3300*** ~~3150~~ doses of the RTS,S/AS02A malaria candidate vaccine
  RTS,S/AS02A will be supplied such that the reconstituted vaccine volume will be 0.5 ml per vial. The contents of each vial will be used to prepare one paediatric dose (0.25 ml dose volume) only.
- ***2400*** ~~2700~~ doses of the Hepatitis B vaccine (Engerix-B™)
- ***300*** ~~150~~ doses of *Haemophilus influenzae* type b vaccine (Hiberix™)
- ***600*** ~~300~~ doses of 7-valent pneumococcal conjugate vaccine (Prevnar®)

**(Amended January 17, 2003)**

These figures include over-randomisation and to which an additional 5% of their respective amounts will be supplied for replacement in case of breakage or bad storage conditions.

All vials and syringes need to be accounted for on the form provided.

Additional doses of Prevnar® and Hiberix™ (commercial presentations) ***and Engerix–B™ (only if required, see section 5.2.12)*** will be provided for cross-over immunisation at the end of the study. Cross-over immunisation will not be considered as a study procedure and therefore will not be documented in the CRFs.
**(Amended January 17, 2003)**

2. Vaccine packaging

The vaccines will be packed in labeled boxes. The box label will contain the following information: study number, subject number, lot numbers, instructions for vaccine administration.

3. Vaccine accountability

The investigator or pharmacist must sign a statement that he/she has received the clinical supplies for the study*.* At any time the figures on supplied, used and remaining vaccine doses should match. At the end of the study, it must be possible to reconcile delivery records with those of used and unused stocks. Account must be given of any discrepancies.

After approval from GlaxoSmithKline, both used and unused vaccine vials should be destroyed at the study site using locally approved biosafety procedures and documentation unless otherwise described in the protocol. If no adequate biosafety procedures are available at the study site, the vaccine vials should be destroyed at an appropriate GlaxoSmithKline site in accordance with SOP CD-3.1 and SOP CD-7.1.

4. Labels for sample identification

The investigator will receive labels to identify samples taken from each subject at each timepoint. The label will contain the following information: study number, subject number, sampling timepoint, timing. Samples will be collected at the following time points:

***Screening -90 to –7***

Pre Day 0

Post III Day 90

Post III Month 8½

Post III Month 21
**(Amended January 17, 2003)**

5. Other supplies provided by the sponsor

In addition to the vaccines, the study documentation and the sample labels, the investigator will receive the following supplies:

- tubes for blood sampling
- tubes with screw caps for serum samples
- racks for the tubes of serum
- syringes and needles for injection

Appendix H: Amendments/Modifications to the Study Protocol

***Protocol Amendment Approval***

***Study Drug***

GlaxoSmithKline Biologicals' candidate *Plasmodium falciparum* malaria RTS,S/AS02 vaccine

***Protocol Number***

257049/026 (Malaria-026)

| Protocol Title: A double-blind randomised controlled phase IIb study to evaluate the safety, immunogenicity and efficacy of GlaxoSmithKline Biologicals' candidate malaria vaccine RTS,S/AS02A, administered IM according to a 0, 1 and 2 month vaccination schedule in toddlers and children aged 1 to 4 years in a malaria-endemic region of Mozambique. |
| --- |

**Final version of protocol: May 8, 2002 (Final version)**

**Amendment 1: August 13, 2002 (Final version)**

| **Coordinating Author: Nicholas Perombelon**  **Rationale/background for changes:**  The protocol has been reviewed by the Institutional Review Board (IRB) of the Malaria Vaccine Initiative at PATH (Human Subjects Protection Committee, HSPC) and the IRB at Barcelona University Hospital, Barcelona, Spain. It has still to be reviewed by the Joint Mozambican Government Ethics Review Committee. The IRB at Barcelona University Hospital requested that the Parent/Guardian Information Sheet and Consent Form for Cohort 2 specify the drugs to be used for malaria treatment and their potential benefits and risks. A statement to this effect was submitted and approved by this IRB on July 17, 2002.  As there were further changes recommended by the IRBs and others requested by the Principal Investigator, this amendment reflects these changes as well as the statement referring to the drugs to be used for malaria treatment and their potential benefits and risks:  Protocol:   - Section 4.4 Exclusion Criteria For Enrolment (review the incorporation of HIV status in the exclusion criteria as it is unknown whether vaccination with RTS,S/AS02A presents an additional risk to HIV positive children) |
| --- |
| Parent/Guardian Information Sheets for Cohorts 1 and 2:   - Section “Risks and Benefits” (clarification that the side effects listed apply to both Hiberix™ and Prevnar®). - Section “Parent/Guardian’s Responsibility” (clarification that if a child is unwell and brought to the health centre, the child will receive treatment for malaria or for any condition that the health post is able to treat). - Section “Leaving the Study” (revision of the study contacts for voluntary removal of a child from the study by the parent/guardian).   Parent/Guardian Consent Forms for Cohorts 1 and 2:   - Paragraph 6 of the Consent form (inclusion of language to reiterate that parents should continue to use normal preventive measures against malaria throughout the study).   Parent/Guardian Information Sheet and Consent Form for Cohort 2:   - Sections “Purpose,” “Risks and Benefits” of Information Sheet and paragraph 3 of the Consent Form (reference is made to malaria treatment to remove all parasites without referring to the specific drugs to be used and their potential benefits and risks).   In addition to these comments from the IRBs, there have been ongoing discussions for the parasite genotyping analysis. To have consistency in sampling for blood slides taken for parasitaemia and for parasite genotyping, blood samples will be kept from the cross-sectional survey at month 8½ (Clinic visit 6) in Cohort 1. This will not entail any additional blood sampling from subjects. There will be sufficient blood taken at Clinic Visit 6 for the preparation of the blood spot necessary for the PCR analysis of the parasite genotype. These samples will be stored and analysed as the other samples taken for parasite genotyping in Cohort 1 and 2.  An inaccuracy in the background information provided on the prevalence of HBV infection has been detected in Section 1.2.1.1 (Concurrent protection against Hepatitis B) and this has been corrected.  The exclusion criterion for severe malnutrition will be determined based on weight for height ≤ -3z scores instead of weight for age (Section 4.4 Exclusion criteria for enrolment).  On request of the Principal Investigator, the provision of study numbers will be from 101 to 2200 instead of 1 to 2100, i.e. study numbers 101 to 400 will be provided for children less than 18 months old and study numbers 401 to 2200 will be provided for children older than 18 months. This change will thus avoid any overlap of study numbers for this study (Malaria-026) and the earlier phase I study (Malaria-025) being performed within the same catchment area as Cohort 1. |

| The instructions for the vaccination of subjects omitted to specify the needle size (25G with length of 1 inch [25 mm]) to be used for all study vaccinations. This has now been corrected.  Several minor errors have been detected in the References in the Study Protocol. These errors have been corrected  Similarly, the Parent/Guardian Information Sheets and Consent Forms inconsistently refer to the health centres to that the parents/guardians should refer their children to. It is now consistently specified that children in Cohort 1should be brought to Manhiça Health Centre and children in Cohort 2 should be brought to Ilha Josina Health Centre.  On request of the Principal Investigator, the text in the Parent/Guardian Information Sheets for Cohorts 1 and 2 has been shortened but without any loss of information, to facilitate the understanding of the parents/guardians receiving these documents.  The Consent Form for Cohort 1 has a paragraph missing concerning the confidentiality of information about the parent/guardian’s child. This has been added to the document.  The Consent Forms for Cohorts 1 and 2 have been reformatted to allow the parents/guardians to date their signatures, if they can sign the document.  The paragraphs in the title pages of the protocol for the approval signatures of GlaxoSmithKline Biologicals and the Principal Investigator have been deleted. |
| --- |

| **Amended text in *bold italics* has been included in the following sections:**  **Study Protocol:**  **Title pages**  **Section 1.2.1.1 Concurrent protection against Hepatitis B**  **Section 1.2.2.2 Rationale for parasite genotyping**  **Section 4.4 Exclusion Criteria For Enrolment**  **Section 5.2.8 Cross sectional surveys**  **Section 5.3 Outline of study procedures for Cohort 1**  **Section 5.4 Detailed description of study stages/visits for Cohort 1**  **Section 5.5 Outline of study procedures for Cohort 2**  **Section 5.6 Detailed description of study stages/visits for Cohort 2**  **Section 5.8.3.1 Laboratory testing for Cohort 1**  **Section 6.2 Dosage and administration**  **Section 6.4 Treatment allocation and randomization**  **References**  **Parent/Guardian Information Sheets and Informed Consent forms for Cohorts 1 and 2** |
| --- |

1. Breman JG. The ears of the hippopotamus: manifestations, determinants and estimates of the malaria burden. Am J Trop Med Hyg 2001; 64 (1,2)S: 1-11. [↑](#endnote-ref-2)
2. Breman JG, Egan A, Keusch GT. Introduction and Summary. The intolerable burden of malaria: a new look at the numbers. Am J Trop Med Hyg 2001; 64 (1,2)S: iv-vii. [↑](#endnote-ref-3)
3. ***Stoute JA, Slaoui M, Heppner DG et al. A preliminary evaluation of a recombinant circumsporozoite protein vaccine against plasmodium falciparum malaria. N Eng J Med 1997;336: 86-91.*** [↑](#endnote-ref-4)
4. Doherty JF, Pinder M, Tornieporth N, *et al.* A phase I safety and immunogenicity trial with the candidate malaria vaccine RTS,S/SBAS02 in semi-immune adults in The Gambia. J Am Soc Trop Med Hyg 1999; 61 (6): 865-68. [↑](#endnote-ref-5)
5. GSK Data on File: Investigator Brochure for SmithKline Beecham Biologicals’ Candidate Vaccine Against Plasmodium Falciparum Malaria (RTS,S/AS02 malaria vaccine CPMS 257049). First edition March 13, 2001. Data on file at GlaxoSmithKline Biologicals. [↑](#endnote-ref-6)
6. Stoute JA, Kester KE, Krzych U, et al. Long-term Efficacy and Immune Responses following Immunization with the RTS,S Malaria Vaccine. Journal of Infectious Diseases 1998; 178: 1139-44. [↑](#endnote-ref-7)
7. Bojang KA, Milligan PJM, Pinder M, et al. Efficacy of RTS,S/AS02 malaria vaccine against *Plasmodium falciparum* infection in semi-immune adult men in The Gambia: a randomised trial. The Lancet 2001; 358:1927-34. [↑](#endnote-ref-8)
8. GSK Final Study Report on file: Phase I/IIa Safety, Immunogenicity, and Preliminary Efficacy of Two Short-Course Schedules of SmithKline Beecham Biologicals’ Candidate Malaria Vaccine RTS,S in Non-Immune Adult Volunteers. Protocol 257049/012 (Malaria-012) February 28, 2002. Data on File at GlaxoSmithKline Biologicals. [↑](#endnote-ref-9)
9. GSK Final Study Report on file: A phase I double blind, randomised controlled, staggered study to evaluate the safety, reactogenicity and immunogenicity of 1/5^th^, ½, and full doses of GlaxoSmithKline Biologicals RTS,S/AS02 candidate malaria vaccine, administered IM according to a 0,1,3-month vaccination schedule in semi-immune children aged 6 to 11 years in The Gambia, a malaria endemic region. Protocol 257049/015 (Malaria-015) April 17, 2002. Data on File at GlaxoSmithKline Biologicals. [↑](#endnote-ref-10)
10. GSK Final Statistical Report on file: A phase I, double-blind, randomized, controlled, staggered study to evaluate the safety, reactogenicity and immunogenicity of 1/5, 1/2, and full doses of GlaxoSmithKline Biologicals RTS,S/AS02 candidate malaria vaccine, administered IM according to a 0,1,3-month vaccination schedule in semi-immune children aged 1 to 5 years in The Gambia, a malaria endemic region. Protocol 257049/020 (Malaria-020) April 18, 2002. Data on File at GlaxoSmithKline Biologicals. [↑](#endnote-ref-11)
11. GSK Data on file: Summary report on the safety of SB AS02-containing vaccines: A summary of safety data from prophylactic and therapeutic studies of SB AS02-containing vaccines May 18, 2001. Data on File at GlaxoSmithKline Biologicals. [↑](#endnote-ref-12)
12. Manhica DSS, Mozambique. In Population and Health in Developing Countries. Volume 1: Population, health, and survival at INDEPTH sites. Canada: International Development Research Centre, 2002:189-195. [↑](#endnote-ref-13)
13. The working group on mother to child transmission of HIV. Rates of mother-to-child transmission of HIV 1 in Africa, America and Europe: results from 13 perinatal studies. Journal of Acquired Immune Deficiency Syndrome Human Retrovirolgy 1995; 8: 506-10. [↑](#endnote-ref-14)
14. Kester KE, McKinney DA, Tornieporth N, et al. Efficacy of recombinant circumsporozoite protein vaccine regimens against experimental Plasmodium falciparum malaria. J Infect Dis 2001; 183(4): 640-7. [↑](#endnote-ref-15)
15. ***Purcell RH. Hepatitis viruses: Changing patterns of human disease. Proc Natl Acad Sci USA 1994; 91: 2401-2406.*** [↑](#endnote-ref-16)
16. Safary A, Andre F. Over a decade of experience with a yeast recombinant hepatitis B vaccine. Vaccine 2000; 18: 57-67. [↑](#endnote-ref-17)
17. ***Smith T Armstrong Schellenberg J Attributable fraction estimates and case definitions for malaria in endemic areas. Stats in Med 1994 13:2345-2358.*** [↑](#endnote-ref-18)
18. Management of Severe Malaria, A Practical handbook. WHO, Geneva; Second edition 2000. [↑](#endnote-ref-19)
19. GSK Document on file: Master Data Sheet for Hiberix™, June 26, 2000. Document on File at GlaxoSmithKline Biologicals. [↑](#endnote-ref-20)
20. Wyeth LederleVaccines’s Prescribing Information Sheet for Prevnar®, Pneumococcal 7-valent Conjugate Vaccine (Diphtheria CRM_197_ Protein). [↑](#endnote-ref-21)
21. Leowski J. Mortality from acute respiratory infection in children under five years of age: global estimates. World Health Statistics Quarterly 1986 39 138-144 [↑](#endnote-ref-22)
22. Greenwood B. The epidemiology of pneumococcal infection in children in the developing world. Phil Trans R Soc Lond 1999; 354: 777-785 [↑](#endnote-ref-23)
23. Hausdorff W, Chang I, Eby R. The epidemiology of invasive pneumococcal disease: implications for conjugate vaccine development (abst). First International Symposium on Pneumococci and Pneumococcal Diseases, Helsingor, June 13-17 1998. [↑](#endnote-ref-24)
24. Goetghebuer T, West TE, Wermenbol V, et al. Outcome of meningitis caused by *Streptococcus pneumoniae* and *Haemophilus influenzae* type b in children in The Gambia Trop Med Int Health 2000; 5(3):207-13 [↑](#endnote-ref-25)
25. ***Bulletin of the WHO, 1998; 76 (suppl 2): 131-132; 113*** [↑](#endnote-ref-26)
26. Hargreaves R, Slack M. Changing patterns of invasive *Haemophilus influenzae* disease in England and Wales after introduction of the Hib vaccination programme. BMJ 1996; 312:160-161 [↑](#endnote-ref-27)
27. Iwarson S et al. Neutralisation of hepatitis B virus infectivity by a murine monoclonal antibody: An experimental study in the chimpanzee. Journal of Medical Virology 1985; 16: 89-96. [↑](#endnote-ref-28)
28. Shann F. Nutritional Indices: Z centile, or percent? Lancet 1993; 341: 526-527 [↑](#endnote-ref-29)
29. GSK Document on file: Terms of Reference (Operating Procedures): Data Safety Monitoring Board, Malaria Vaccine Studies, GSK Biologicals, October 26 2001. Document on File at GlaxoSmithKline Biologicals. [↑](#endnote-ref-30)
30. ***Smith PG, Morrow RH. Methods for Field Trials of Interventions against Tropical Diseases: A Toolbox. Oxford University Press 1991: 152-155; 168-175.*** [↑](#endnote-ref-31)
31. WHO, 1963 Terminology of Malaria [↑](#endnote-ref-32)
32. Teuscher T, Armstrong Schellenberg JRM, Bastos de Azevedo I, et al. SPf66, a chemically synthesized subunit malaria vaccine, is safe and immunogenic in Tanzanians exposed to intense malaria transmission. Vaccine 1994; 12:328-336. [↑](#endnote-ref-33)
33. Trager W, Jensen JB. Human malaria parasites in continuous culture. Science 1976; 193: 673-675. [↑](#endnote-ref-34)
34. Blackwelder WC Sample size and power for prospective analysis of relative risk. Statistics in Medicine 1993; 12:691-698. [↑](#endnote-ref-35)
35. GSK Final Study Report on file: Double-blind, randomized study to evaluate the immunogenicity and reactogenicity of three lots of SmithKline Beecham Biologicals' combined tetravalent diphtheria, tetanus, whole cell B. pertussis, 10 µg HBsAg hepatitis B vaccine (DTPwHBV) in healthy infants vaccinated at 3, 4 and 5 months of age. Protocol 208139/033 (DTPwHBV-033) March 21, 1994. Data on File at GlaxoSmithKline Biologicals. [↑](#endnote-ref-36)
36. ***Therneau TM, Grambsch PM. Modeling survival data. Multiple events per subject. In: Therneau TM, Grambsch PM. Modeling survival data. Extending the Cox model. New York: Springer , 2000.*** [↑](#endnote-ref-37)
37. Lin DY, Wei LJ. The robust inference for the Cox proportional hazards model. J. Amer. Stat. Assoc. 1989; 84: 1074-78.

    **(Amended July 23, 2002)** [↑](#endnote-ref-38)
38. The Serum Listing Form and the Specimen Transfer Form are standard documents used in GlaxoSmithKline Biologicals’ clinical trials. These documents can be found in the binder entitled ‘Standard Forms in GlaxoSmithKline Biologicals’ Clinical Trials’ under ‘Logistics’. [↑](#footnote-ref-2)
